# Natural Allelic Diversity, Genetic Structure and Linkage Disequilibrium Pattern in Wild Chickpea

**DOI:** 10.1371/journal.pone.0107484

**Published:** 2014-09-15

**Authors:** Maneesha S. Saxena, Deepak Bajaj, Alice Kujur, Shouvik Das, Saurabh Badoni, Vinod Kumar, Mohar Singh, Kailash C. Bansal, Akhilesh K. Tyagi, Swarup K. Parida

**Affiliations:** 1 National Institute of Plant Genome Research (NIPGR), Aruna Asaf Ali Marg, New Delhi, India; 2 National Research Centre on Plant Biotechnology (NRCPB), New Delhi, India; 3 National Bureau of Plant Genetic Resources (NBPGR), New Delhi, India; University of Delhi South Campus, India

## Abstract

Characterization of natural allelic diversity and understanding the genetic structure and linkage disequilibrium (LD) pattern in wild germplasm accessions by large-scale genotyping of informative microsatellite and single nucleotide polymorphism (SNP) markers is requisite to facilitate chickpea genetic improvement. Large-scale validation and high-throughput genotyping of genome-wide physically mapped 478 genic and genomic microsatellite markers and 380 transcription factor gene-derived SNP markers using gel-based assay, fluorescent dye-labelled automated fragment analyser and matrix-assisted laser desorption ionization-time of flight (MALDI-TOF) mass array have been performed. Outcome revealed their high genotyping success rate (97.5%) and existence of a high level of natural allelic diversity among 94 wild and cultivated *Cicer* accessions. High intra- and inter-specific polymorphic potential and wider molecular diversity (11–94%) along with a broader genetic base (13–78%) specifically in the functional genic regions of wild accessions was assayed by mapped markers. It suggested their utility in monitoring introgression and transferring target trait-specific genomic (gene) regions from wild to cultivated gene pool for the genetic enhancement. Distinct species/gene pool-wise differentiation, admixed domestication pattern, and differential genome-wide recombination and LD estimates/decay observed in a six structured population of wild and cultivated accessions using mapped markers further signifies their usefulness in chickpea genetics, genomics and breeding.

## Introduction

Chickpea [*Cicer arietinum* (L.)] is one of the most important food legume crops in the world and serves as an important source of protein with essential amino acids in human diet. To improve the productivity and sustainability of agriculture, the development of high-yielding durable stress tolerant (climate resilient) cultivars is the prime objective in current chickpea genomics and molecular breeding research. The systematic and extensive breeding efforts of chickpea have resulted in the development of a number of high-yielding stress tolerant cultivars through intra- and inter-specific hybridization of a few selected germplasm accessions. Therefore, the genetic base of modern chickpea cultivars is narrow which in turn limits the yield enhancement and increased stress tolerance. Furthermore, the earlier archaeological, phenotypic and molecular phylogeny studies inferred that chickpea domestication might have led through maximum adaptation-based selection pressure, followed by punctuation by a series of four sequential evolutionary bottlenecks during their evolutionary transition (∼10,000 years) from wild *C. reticulatum*, which progressively narrows down the genetic base in cultivated gene pool [1]–[5]. Chickpea belonging to the genus *Cicer* includes many annual and perennial wild species that represent a valuable and potential source of diverse gene pool for improving yield and abiotic/biotic stress tolerance in cultivated chickpea [6]–[11]. The genetic improvement of chickpea thus necessitates the identification and exploitation of its genetically diverse wild relatives with beneficial traits to broaden the genetic base of modern cultivars though inter-specific hybridization. The significance of inter-specific hybridization by introducing the desirable target traits of agricultural importance specifically from wild species of primary gene pool to cultivated varieties for enhancing their seed and pod yield and stress tolerance have been demonstrated in chickpea [7], [8], [10], [12]. However, producing these successful inter-specific hybrids relied upon combinations of accessions selected from each of the cultivated and wild species for cross-pollination and their level of genetic variability to ascertain suitable cross-compatibility.

The genetic diversity and evolutionary relationships among different annual and perennial *Cicer* species have been studied based on agro-morphological traits, inter-specific hybridization, karyotyping, seed storage protein and isozyme profiling [13]–[24]. Additionally, the utility of DNA-based markers for unbiased estimation of molecular diversity and establishing precise phylogenetic relationships among cultivated and wild *Cicer* species as compared to morphological, cytological and biochemical markers have been well understood [25]–[31]. The PCR-based random molecular markers like RAPD (random amplified polymorphic DNA) and AFLP (amplified fragment length polymorphism) have also been utilized to understand the genetic diversity pattern and phylogenetic relationships among annual and perennial *Cicer* species [25]–[31]. All these diversity studies have a general consensus regarding classification of annual and perennial *Cicer* species into three crossability groups comprising of primary, secondary and tertiary gene pools with certain controversies on phylogenetic origination of *C. echinospermum*, *C. bijugum* and *C. pinnatifidum* from these gene pools. Moreover, these studies documented narrow genetic variation within cultivated and wild *Cicer* species due to the inclusion of limited number of accessions per species and use of less informative random marker systems.

The desirable genetic attributes (co-dominant inheritance, reproducibility, bi-/multi-allelic nature and abundant genomic distribution) of sequence-based robust microsatellite/simple sequence repeat (SSR) and single nucleotide polymorphism (SNP) markers have encouraged their utilization for many applications of chickpea genetics, genomics and breeding including cultivar identification, genetic diversity analysis and establishing phylogenetic relationships among cultivated and wild species [32]–[40]. However, all these studies have utilized a limited number of genomic and genic microsatellite and SNP markers (∼200 markers) with no prior information on their chromosomal distribution for assaying a smaller set of accessions (at most 20) specifically belonging to three different wild annual *Cicer* species (*C. reticulatum*, *C. echinospermum* and *C. bijugum*) of the primary gene pool. Therefore, such studies are not able to infer realistic estimate of genetic variability existing among the accessions within individual wild and cultivated *Cicer* species. Rather, they produced ambiguous molecular diversity and phylogenetic information with no clear differentiation among evolutionarily closely related wild *Cicer* accessions belonging mainly to the primary gene pool, which has higher cross-compatibility with cultivated species. An understanding of the extent of natural diversity and phylogenetic relationships among accessions belonging to each of the primary, secondary and tertiary gene pools is essential to develop novel breeding strategies for chickpea genetic improvement. Accordingly, a large number of genic and genomic microsatellite and SNP markers showing genome-wide physical distribution (on a bp-scale) as well as representing LD (linkage disequilibrium) and haplotype blocks on the eight chromosomes of chickpea are required for monitoring introgression and broadening the genetic base of chickpea cultivars through inter-specific hybridization. It could thus expedite the process of transferring novel genes/alleles of agronomic importance from wild to cultivated chickpea species for their genetic improvement.

The recent released draft genome sequences of *desi* and *kabuli* chickpea cultivars [41], [42] could help to select a large number of genome-wide microsatellite and SNP markers from pseudomolecules and LD/haplotype blocks of eight chromosomes based on their physical positions (bp) for realistic estimation of molecular diversity and phylogenetic relationships among cultivated and wild *Cicer* accessions. Recently, thousands of SNPs discovered by RAD (restriction site-associated DNA) sequencing of four *C. reticulatum* and one *C. echinospermum* wild accessions distributed over eight chickpea chromosomes have also been utilized to understand their population genetic structure and diversity pattern as compared with cultivated *C. arietinum* accessions [42]. However, this study could not detect species-wise differentiation/groupings of cultivated and wild accessions possibly due to use of few selected (five) wild accessions for genotyping and diversity analysis. Moreover, no systematic efforts has yet been made to understand the existing molecular diversity pattern, population genetic structure and phylogenetic relationships among a larger and diverse set of wild *Cicer* accessions based on functionally relevant genic regions of their genome that are more prone to phenotypic selection during chickpea domestication. Mining of novel allelic variants from wild accessions and their large-scale validation through high-throughput genotyping of suitable informative microsatellite and SNP markers is also imperative, which will facilitate identification of diverse wild germplasm with beneficial traits to broaden the genetic base of cultivars and enhancing the genetic potential of chickpea globally.

Keeping all above in view, the present study was undertaken to validate and genotype genome-wide well distributed genomic and gene-derived (transcription factor) microsatellite and SNP markers that are physically mapped (bp) across eight chickpea chromosomes in 94 wild and cultivated *Cicer* accessions using gel-based assay, fluorescent dye-labelled automated fragment analyzer and 34-plex MALDI-TOF (matrix-assisted laser desorption ionization-time of flight) mass array. The genotyping information of validated markers was further utilized for understanding the functional molecular diversity pattern, population genetic structure and phylogenetics, and determining the genome-wide and population-specific LD (linkage disequilibrium) patterns specifically in wild *Cicer* accessions.

## Materials and Methods

### Plant resources and genomic DNA extraction

A total of 94 different *Cicer* accessions belonging to one annual cultivated *C. arietinum* (12 accessions), five annual wild species, namely *C. reticulatum* (16), *C. echinospermum* (8), *C. judaicum* (22), *C. bijugum* (19) and *C. pinnatifidum* (16) and one perennial species *C. microphyllum* (**Table 1** **,** **Table S1**) were used for large-scale validation and genotyping of microsatellite and SNP markers. The genomic DNA was isolated from the young leaf samples of 94 accessions using QIAGEN DNeasy 96 Plant Kit (QIAGEN, USA) following the manufacturer's instructions. Despite of wide availability of wild chickpea accessions in different Genebanks, poor viability, germination and regeneration efficiency largely limits their utilization. Henceforth, we were able to use only 82 accessions belonging to six annual and perennial wild chickpea species for understanding their diversity and domestication patterns.

**Table 1:** Some passport information on global wild and cultivated annual *Cicer* accessions used in the present study.

S. No.	Species	Biological status	Breeding cycle	Gene pool	Inbreeding	Geographical origin	Natural habitats
1	*C. arietinum*	Cultivated (*desi* and *kabuli*)	Annual	Primary	Inbreeder	India	Terrestrial (upland and non-aquatic)
2	*C. reticulatum*	Wild	Annual	Primary	Inbreeder	Turkey	Limestone hills, oak scrub and vineyards
3	*C. echinospermum*	Wild	Annual	Primary	Inbreeder	Turkey and Syrian Arab Republic	Rocky slopes, orchards and fields
4	*C. judaicum*	Wild	Annual	Secondary/tertiary	Outbreeder	India, Lebanon, JordanSyrian Arab Republic and Israel	Rocky slopes and fields
5	*C. bijugum*	Wild	Annual	Secondary/tertiary	Outbreeder	Turkey and Syrian Arab Republic	Orchards and fields
6	*C. pinnatifidum*	Wild	Annual	Secondary/tertiary	Outbreeder	Syrian Arab Republic and Turkey	Rocky slopes, vineyards and parent rock (igneous and limestone)
7	*C. microphyllum*	Wild	Perennial	Tertiary	Outbreeder	India and Turkey	Near glaciers in the Hindu Kush Himalayas

### Large-scale validation and genotyping of microsatellite markers

A set of 343 microsatellite markers designed from gene sequences including transcription factors (TFs) of *C. arietinum desi* ICC 4958 [41] and *C. arietinum kabuli* CDC Frontier [42] and 153 genomic microsatellite markers were selected based on their genome-wide physical distribution on eight chromosomes. These designed markers were PCR amplified using genomic DNA of 94 accessions (**Table 1**, ****). The constituents and cycling conditions used for PCR amplification were described by Jhanwar et al. [43] and Agarwal et al. [44]. The amplified PCR fragments were resolved in 3.5% metaphor agarose gel and automated fragment analyzer. For performing automated fragment analysis, the fluorescent dye-labelled microsatellite markers were resolved in ABI3730xl DNA analyzer following the procedure of Kujur et al. [45]. The electrophoregram containing trace files generated for each microsatellite marker were analysed in GeneMapper V4.0 (Applied Biosystems, USA) with optimized parameters and automatic allele calling was performed. The actual allele size (bp) and fragment length polymorphism detected by microsatellite markers in 94 accessions based on gel-based assay and automated fragment analysis were obtained.

### Discovery and high-throughput genotyping of TF gene-derived SNPs

A set of 500 TF-encoding genes of ICC 4958 and CDC Frontier were selected based on their genome-wide physical localization (bp) on eight chickpea chromosomes. The forward and reverse primers targeting the functional domain and upstream regulatory regions of TF genes with amplification product size of 500 to 700 bp were designed employing Batch Primer3 (http://probes.pw.usda.gov/batchprimer3). The synthesized 500 gene-based markers were amplified using the genomic DNA of ICC 4958 and ICC 17160 and resolved in 1.5% agarose gel. The amplified PCR product of 479 markers showing clear, reproducible and monomorphic amplification between two chickpea accessions (ICC 4958 and ICC17160) were purified and sequenced in both forward and reverse directions at least two times on ABI (Applied Biosystems) 3730 xl automated DNA sequencer using the target gene-based primers. The high-quality consensus sequences with high *phred* (≥30) and *phrap* (≥80) scores thus obtained were compared among two accessions and SNPs were detected in the TF genes employing the integrated software tool *polyphred* with ≥99% accuracy score. The SNPs detected through *polyphred* were further manually inspected and confirmed by aligning the high quality FASTA sequences between two chickpea accessions through CLUSTALW multiple alignment and graphical interface tools of BIOEDIT (http://www.mbio.ncsu.edu/BioEdit/bioedit.html). Based on these analyses, finally a set of high-quality 384 SNP loci (one in each TF genes) differentiating ICC 4958 and ICC 17160 was selected.

These TF gene-derived 384 SNPs were further genotyped in the genomic DNA of 94 cultivated and wild *Cicer* accessions (**Table S1**) using Sequenom MALDI-TOF MassARRAY system (http://www.sequenom.com). Reproducibility of SNP genotyping assays were evaluated using seven samples (accessions) as biological replicates, one each representing the wild and cultivated *Cicer* species. The target SNP loci along with their flanking 100 bp sequences were curated for individual genes. The Sequenom MassARRAY multiplex iPLEX assay including pre-amplification primary forward and reverse PCR primers and one unextended primer (UEP) [act as single nucleotide extension primers (EP)] for each 384 SNP loci were designed using MassARRAY Assay Design software v3.1. All other default parameters except for the optimal amplicon size containing the SNP sites of 80–120 bp were set in the software for analysis. The EP and UEP were optimized for 34-plex assays and procured oligos from IDT, USA. A 10-mer tag (5′-ACGTTGGATG-3′) was added to the 5′-end of each forward and reverse pre-amplification primers to improve the PCR performance and mass spectrometry. The amplification of synthesized pre-amplification primers with multiplex PCR assay, shrimp alkaline phosphate (SAP) incubation for neutralizing unincorporated dNTPs, primer extension, resin cleanup and mass spectrometry were performed following the manufacturer's instructions of Sequenom iPLEX Gold amplification kit (http://www.sequenom.com). The SNP genotyping results obtained in iPLEX spectrochip bio-arrays were analyzed using MassARRAY Typer 3.4 software tool. The different SNP alleles among 94 accessions were visualized and documented based on allele-specific differences in mass between extension products.

### Assessment of polymorphic potential, molecular diversity and population genetic structure

The genotyping information of validated 478 microsatellite markers and 380 TF gene-derived SNP markers was analysed in PowerMarker v3.51 [46] to estimate the polymorphic allele number/locus, major allele frequency, per cent polymorphism, PIC (polymorphism information content), gene diversity [measured as expected heterozygosity (He)] and heterozygosity. Intra-specific polymorphic potential of markers within individual species was estimated as proportion of total number of alleles amplified by microsatellite and SNP markers showing polymorphism among accessions belonging to each of seven wild and cultivated *Cicer* species [45], [47]. The molecular basis of microsatellite marker fragment length polymorphism across wild and cultivated species based on expansion/contraction of repeat-units at their target locus and/or marker size homoplasy (as expected particularly in case of diverse inter-species comparison) was inferred. For this, the PCR products of 50 randomly selected genic and genomic microsatellite markers amplified from one accession representing each of seven different wild and cultivated species were cloned and sequenced twice in both forward and reverse direction through a capillary-based automated DNA sequencer and the derived high quality sequences were aligned and compared among accessions following the methods of Kujur et al. [45].

The genotyping data of 858 markers (including 478 microsatellite and 380 SNP markers) physically mapped across eight chromosomes of chickpea were used to determine functional molecular diversity and population structure and for establishing genetic relationships among 94 cultivated and wild *Cicer* accessions. The cluster analysis among accessions was performed based on Nei's genetic distance [48] employing the neighbour-joining (NJ) method (with 1,000 bootstrap replicates) in PowerMarker v3.51 and unrooted phylogenetic tree was visualized using MEGA v4.0 [49]. For deriving significant correlation between microsatellite and SNP markers based on their potential to detect molecular diversity, the genetic distance estimated for all pair-wise combinations of 94 accessions using these markers was plotted individually against each other and compared using the Mantel test [50].

The genome-wide 858 marker genotyping data generated from 94 wild and cultivated *Cicer* accessions were used in a model based program STRUCTURE v2.3 [51] employing the admixture and correlated allele frequency with a burn-in of 100,000 iterations and run length of 100,000. Analysis of population genetic structure among accessions was carried out using the Bayesian clustering algorithm of STRUCTURE with varying levels of K (number of populations)  = 1 to 10. At the most, we expected up to 10 populations (K = 10) could be due to clustering of 94 accessions into six distinct population groups based on our preliminary genetic distance-based phylogenetic tree analysis. The average of likelihood value, Ln P(D) against each K across 20 independent replications was estimated and plotted. The optimal value of K was determined according to *ad hoc* method of Pritchard et al. [51] and delta K procedure of Evanno et al. [52]. Using the optimum K, the population structure model representing better relationships among 94 cultivated and wild *Cicer* accessions was constructed. Various population genetic parameters including the efficiency of markers for estimating genetic divergence (F_ST_) and degree of admixture among different population groups was estimated. Analysis of molecular variance (AMOVA) among accessions and populations, and within populations and accessions was performed using both GenALEX 6.4 [53] and PowerMarker [50].

### Estimation of LD patterns

The correlation in frequency among pairs of alleles across a pair of 858 marker loci that are physically mapped on eight chromosomes were analysed using TASSEL v2.1 [54] to determine genome-wide LD patterns as r^2^ (average correlation coefficient) among 94 cultivated and wild *Cicer* accessions belonging to diverse population groups (as inferred by population structure). The significance (*P*-value) of LD estimates was determined at 100,000 permutations. The marker loci mapped on the same and different chromosomes were considered as linked and unlinked markers, respectively. The LD was estimated using the genotyping information of linked, unlinked and global (both linked and unlinked) markers. To determine the LD occurrence due to physical linkage of marker loci, 99^th^ percentile of r^2^ distribution for unlinked markers was considered as background level of LD [55]. The decay of LD with the physical distance was measured by combining the r^2^ values of marker-pairs located in the physical intervals of 0–200, 200–400, 400–600, 600–800, 800–1000 and 1000–1200 kb across eight chromosomes of chickpea. The graph was plotted between pooled r^2^ and physical distance (kb) based on nonlinear regression model considering the r^2^ value  = 1 at marker physical distance of 0 kb [55], [56] and finally the trend of LD decay was estimated in both cultivated and wild *Cicer* accessions. The statistical significance test comparing r^2^ values of LD across population groups was performed using ANOVA (analysis of variance) interface tool of SPSS v17.0 (http://www.spss.com/statsistics, IBM SPSS Inc. for window, Chicago, USA).

## Results

### Large-scale validation and high-throughput genotyping of microsatellite and SNP markers

**Figure 1:**
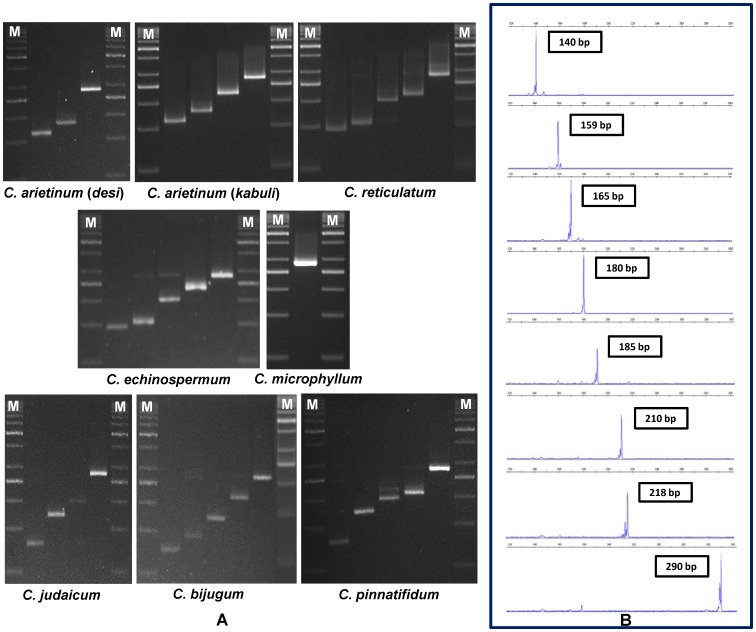
Microsatellite marker-based genotypic variation among wild and cultivated chickpea. Allelic variation detected among a representative set of cultivated and wild accessions belonging to seven *Cicer* species using normal unlabeled and fluorescent dye-labelled genic and genomic microsatellite markers by gel-based assay (A) and automated fragment analyzer (B), respectively. A maximum of 13 polymorphic alleles were amplified by markers among 94 accessions using the gel-based assay and automated fragment analyzer. The fragment sizes (bp) for all the alleles are indicated. M: 50 bp DNA size standard.

**Figure 2:**
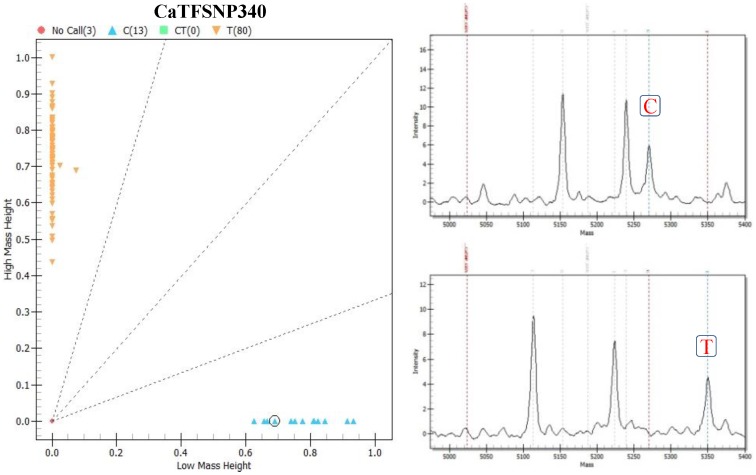
SNP marker-based genotypic variation among wild and cultivated chickpea. Call cluster plots for two representative SNP loci (C/T) demonstrating the genotyping information of all 94 cultivated and wild *Cicer* accessions assayed with MALDI-TOF mass array. Distinct differentiation of homozygous and heterozygous SNPs based on mass differences of corresponding alleles are evident.

A set of 496 (including 343 genic and 153 genomic microsatellite markers) microsatellite markers distributed (physically mapped) across eight chickpea chromosomes showing reproducible amplification were genotyped in 94 cultivated and wild *Cicer* accessions **(** **Table 1** **)** using the agarose gel-based assay and fluorescent dye-labelled automated fragment analyser **(** **Figure 1** **)**. Four hundred seventy-eight (including 334 genic and 144 genomic microsatellite markers) of 496 microsatellite markers (96%) showed polymorphism among 94 *Cicer* accessions **(Table S2)**. Moreover, we discovered 384 novel TF gene-derived high-quality SNPs **(Table S3)** showing differentiation between ICC 4958 and ICC 17160 based on amplicon sequencing. These identified SNPs showing genome-wide physical distribution (bp) on eight chickpea chromosomes were genotyped on 94 cultivated and wild *Cicer* accessions using a maximum of 34-plex **(Figure S1)** high-throughput MALDI-TOF Sequenom mass array genotyping assay. The optimization and genotyping of these multiplex assays on 94 accessions validated 380 SNPs with high degree of reproducibility (100%) and genotyping success rate (98.9%). For each SNP locus, a call cluster plot was generated depicting the low and high mass homozygote and heterozygous yields **(** **Figure 2** **)**. The derived genotype calls of each accessions were automatically called and compared the masses of the peaks with the pre-determined masses estimated during pre-PCR amplification and single nucleotide extension primers designing. The multiplex assay of 384 SNPs was overall successful in discriminating homozygous and heterozygous alleles for 380 SNP loci **(** **Figure 2** **)**, which produced 35720 genotype calls showing polymorphism among 94 accessions. The remaining four SNPs with 376 genotyping data were identified either as monomorphic or without genotype calls. The functional annotation of experimentally validated informative 334 microsatellite markers- and 380 SNP markers-containing genes revealed their maximum correspondence with TF gene families like bZIP (basic leucine zipper), bHLH (basic helix-loop-helix) and AP2-EREBP (APETALA2 ethylene-responsive element binding proteins) (525, 73.5%), followed by growth and metabolism-related enzymes (170, 23.8%) and expressed proteins (19, 2.7%) **(Figure S2)**.

### Intra-/Inter-specific polymorphic potential of microsatellite and SNP markers

The large-scale validation and genotyping of 880 comprising of 496 microsatellite and 384 SNP markers identified 858 (97.5%) markers (including 478 microsatellite and 380 SNP markers) as informative showing polymorphism among 94 cultivated and wild *Cicer* accessions **(Figure S1)**. These polymorphic markers amplified a total of 3703 alleles which varied from 2 to 13 with an average of 4.26 alleles per marker locus **(Table S4)**. The PIC and gene diversity estimated for the informative microsatellite and SNP markers ranged from 0.12 to 0.98 and 0.32 to 0.99 with a mean of 0.56 and 0.60, respectively. Even though the extent of polymorphism detected by microsatellite (96.4%) and SNP (98.9%) markers among 94 accessions was comparable with each other, the microsatellite markers, however revealed higher allele numbers (2–13; mean: 6), PIC (0.18–0.98; mean: 0.75) and gene diversity (0.18–0.99; mean: 0.80) in contrast to that estimated using SNP markers (allele number: 1–2; mean: 1.9, PIC: 0.02–0.47; mean: 0.31, gene diversity: 0.22–0.60; mean: 0.35) (**Table S4**). The heterozygosity and major allele frequency among accessions detected by both microsatellite and SNP markers varied from 0.01 to 0.32 and 0.20 to 1.0 with an average of 0.02 and 0.70, respectively **(Table S4)**. Maximum heterozygosity and major allele frequency among accessions were detected by SNP markers (0.04, 0.83) rather than by microsatellite markers (0.0007, 0.51). Inter-specific polymorphism between cultivated and wild species (467, 94.2% polymorphism; allele numbers: 2–11 with mean: 5, mean PIC: 0.52 and mean gene diversity: 0.55) was higher in contrast to intra-specific polymorphism within cultivated (*desi* and *kabuli*) and wild species (158, 18% polymorphism; allele number: 1–4 with mean: 2.0, mean PIC: 0.25 and mean gene diversity: 0.26). Intra-specific polymorphic potential detected by markers was maximum in *C. reticulatum* (90.2% polymorphism, allele numbers: 2 to 8, PIC: 0.47 and gene diversity: 0.52), followed by *C. judaicum* (71.4%, 2 to 7, 0.42, 0.45) and minimum in *C. echinospermum* (49.3%, 2 to 5, 0.34, 0.36) **(Table S4)**. Maximum heterozygosity of markers was observed in wild *C. judaicum* (0.035), while it was completely absent in the cultivated species. In spite of detecting low PIC, gene diversity and allele numbers by SNP markers as compared to microsatellite markers, the trend of intra- and inter-specific polymorphism observed using combined analysis of microsatellite and SNP markers among 94 *Cicer* accessions remained alike with that of individual marker assays. The degree of polymorphism detected by markers among the species/accessions belonging to secondary gene pool (58.4% polymorphism, mean PIC: 0.42) was higher compared to primary gene pool (47.6%, 0.31). We classified the 94 cultivated and wild accessions under study into two groups as Fertile Crescent (Turkey, Syrian Arab Republic, Jordan, Lebanon and Israel) and Central Asia (India) based on their corresponding geographical origin. Remarkably, the markers revealed much higher polymorphic potential among the species/accessions originating from Fertile Crescent (79.5% polymorphism, mean PIC: 0.50) in contrast to those from Central Asia (35.8%, 0.32).

The cloning and sequencing of size variant amplicons of a selected 50 microsatellite marker sets from the *Cicer* accessions revealed exact correspondence of fragment length polymorphism (bp) detected by markers among seven diverse wild and cultivated *Cicer* accessions with expansion and contraction of their repeats (**Figure S3**). However, substitutions of single nucleotides in the sequences flanking the microsatellite repeat-motifs across seven wild and cultivated *Cicer* accessions (**Figure S3**) was evident.

### Molecular diversity, population genetic structure, species/gene-pool-wise domestication pattern and phylogenetics in chickpea

The pair-wise distance matrix among 94 wild and cultivated accessions belonging to seven different *Cicer* species utilizing the genotyping information of genome-wide well distributed (physically mapped on eight chromosomes) 858 markers (including 478 genic and genomic microsatellite markers and 380 TF gene-based SNP markers) was estimated. The microsatellite marker-based genetic distance among 94 accessions varied from 0.11 to 0.94, while SNP marker-based genetic distance in these accessions ranged from 0.10 to 0.42. A strong correlation (R^2^ = 0.77) between genetic distance estimated by two marker types (microsatellite and SNP markers) based on allele sharing for all pairs of accessions was evident (**Figure S4**). The use of both microsatellite and SNP markers revealed a broad range of genetic distance from 0.11 (*kabuli* cv. ICCV96329 - *kabuli* cv. BG2024) to 0.94 (*C. reticulatum* cv. ICC17160 - *C. judaicum* cv. ILWC283) with an average of 0.62. Remarkably, we observed a wider range of genetic distance (0.13 to 0.78 with a mean: 0.58) among 94 accessions using genic microsatellite and SNP markers. The mean genetic distance between cultivated and wild species varied from 0.44 (*C. arietinum* - *C. microphyllum*) to 0.70 (*C. arietinum* - *C. judaicum*), while between wild species it ranged from 0.54 (*C. echinospermum* - *C. bijugum*) to 0.68 (*C. reticulatum* - *C. judaicum*) **(Table S5)**. Maximum average genetic distance was observed among the accessions within *C. reticulatum* (0.52), followed by *C. judaicum* (0.46) and minimum within *C. bijugum* (0.35). The cultivated *desi* and *kabuli* accessions belonging to *C. arietinum* had least genetic diversity as evident from their pair-wise genetic distance (0.26) **(Table S5)**. The species/accessions included under secondary gene pool (mean genetic distance: 0.46) had higher molecular diversity compared to primary gene pool (0.37). A greater genetic diversity among the accessions/species originated from Fertile Crescent (mean genetic distance: 0.47) in contrast to those from Central Asia (0.23) was evident.

**Figure 3:**
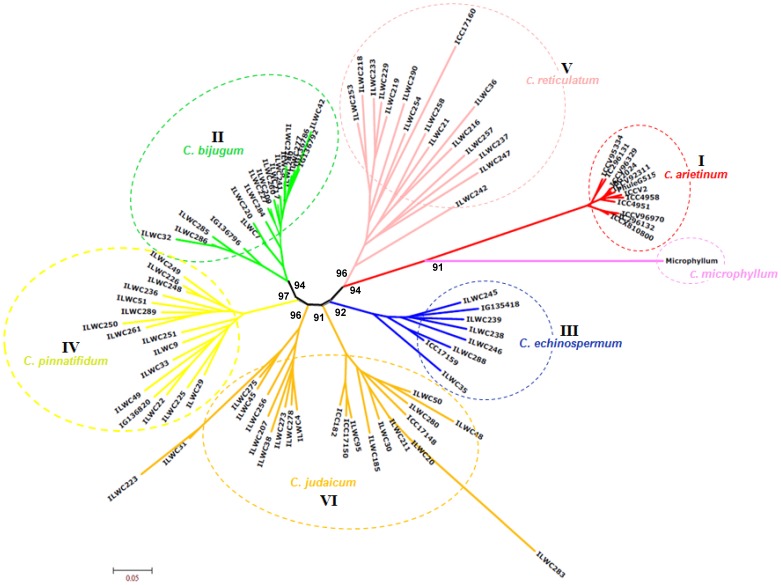
Microsatellite and SNP marker-based genetic diversity and phylogeny among wild and cultivated chickpea. Unrooted phylogenetic tree depicting the genetic relationships among 94 cultivated and wild accessions belonging to seven *Cicer* species based on Nei's genetic distance using 334 genic and 144 genomic microsatellite markers and 380 TF gene-derived SNP markers. Molecular classification clearly differentiated 94 accessions into six different clusters, which corresponds to their species and gene pools of origination. Percentages of confidence obtained in bootstrap analysis are indicated at the corresponding node for each cluster.

The genetic relationships among 94 *Cicer* accessions were depicted in a neighbour-joining unrooted phylogram **(** **Figure 3** **)**. The microsatellite and SNP markers clearly differentiated all the accessions from each other and resulted in a definite grouping of six clusters (I–VI) according to their species and gene pools of origination with high boot strap values (91–97). However, no distinct differentiation among accessions was observed by use of only TF gene-based SNP markers **(Figure S5)**. Even though microsatellite markers could differentiate all 94 accessions from each other, they clustered such accessions randomly into only six different groups with much deviation from their species/gene pools of origination **(Figure S6)**. Interestingly, we identified a minimum of two genomic and five genic microsatellite markers and two TF gene-derived SNP marker combinations as most informative (higher PIC 0.79 to 0.96 and allele numbers 8 to 13), which could discriminate all the 94 accessions representing seven different *Cicer* species from each other **(Table S2)**. The cultivated species *C. arietinum* was highly divergent from other five wild species grouped in separate clusters with 91% occurrences, but was more closer to *C. reticulatum* and *C. echinospermum* and one Indian originated accession of *C. microphyllum*.

**Figure 4:**
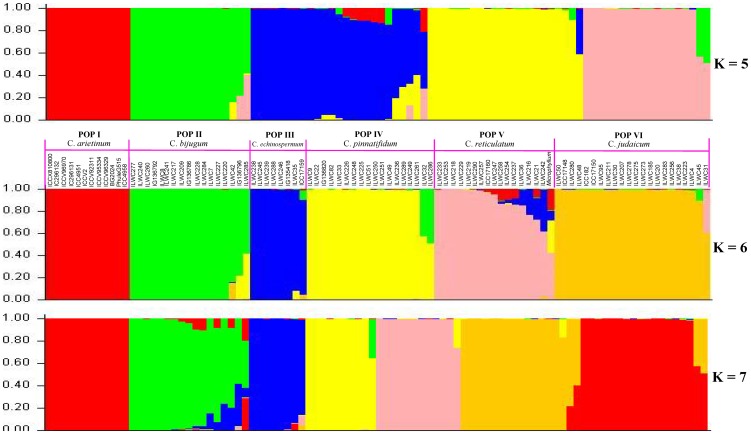
Microsatellite and SNP marker-based assessment of population genetic structure among wild and cultivated chickpea. Optimization of population structure with varying population number K = 5 to K = 7 and their inferred best possible population genetic structure for 94 cultivated and wild *Cicer* accessions using 334 genic and 144 genomic microsatellite markers and 380 TF gene-derived SNP markers physically mapped across eight chromosomes. These mapped markers assigned 94 accessions into six population that majorly grouped accordingly by their species and gene pools of origination. The accessions represented by vertical bars along the horizontal axis were classified into K colour segments based on their estimated membership fraction in each K cluster. Six diverse colours represent different population groups based on optimal population number K = 6.

The population genetic structure among 94 cultivated and wild *Cicer* accessions was determined using genome-wide well distributed (physically mapped on eight chickpea chromosomes) 858 markers employing STRUCTURE. The admixture model-based STRUCTURE simulations were conducted at varying levels of possible population numbers (K = 1 to 10) with 20 replications. The average LnP(D) (log-likelihood) value increased continuously with the increase in K from 1 to 10, however its most apparent inflection was obtained at one of the best replicate of K = 6 **(Figure S7A)**. The results of population numbers (K) were further confirmed using the second order statistics of STRUCTURE by ΔK estimation.^77^ A sharp peak with a maximum value of ΔK was obtained at K = 6 **(Figure S7B)**, which confirms the classification of 94 accessions into six genetically distinct population groups (POP I–VI) with high resolution population structure (**Figure 4**). The POP I contained 12 cultivated *desi* and *kabuli* accessions of *C. arietinum* and one accession of *C. microphyllum*. The POP II, III, IV, V and VI included accessions from *C. bijugum* (19 accessions), *C. echinospermum* (8), *C. pinnatifidum* (16), *C. reticulatum* (16) and *C. judaicum* (22), respectively. The STRUCTURE-based six population group assignment of 94 accessions was further comparable to their clustering patterns, species and gene pools of origination and phylogenetic relationships **(** **Figure 4**) as obtained by the neighbour-joining tree analysis using pair-wise genetic distances. A strong correlation between allele sharing-derived genetic distances estimated individually by microsatellite and SNP markers among six population groups was clearly evident based on Mantel test (0.81). The estimation of polymorphic potential of microsatellite and SNP markers among six population groups revealed their maximum polymorphism in population group, POP V (allele numbers: 2–8 with mean: 3.3 and PIC: 0.05–0.86 with mean: 0.37), followed by POP VI and minimum in POP I **(** **Table 2** **)**. The molecular genetic variation among and within six populations using 858 markers showed a wider level of significant quantitative genetic differentiation based on pair-wise genetic distance and F_ST_ estimation **(Table S5 and S6)** suggesting the existence of broader population differentiation in 94 *Cicer* accessions. The genetic distance varied from 0.31 between POP II and POP III to 0.86 between POP I and POP VI. Similarly, the pair-wise F_ST_ (P<0.001) ranged from 0.23 between POP II and POP III to 0.89 between POP I and POP VI (**Table S6**). Notably, the trends of significant genetic divergence detected by F_ST_ among six population groups was consistent with that of genetic distance **(Table S6)**. The population differentiation based on AMOVA revealed that 7.5%, 4.5% and 2.6% (P<0.01) of total molecular variation in the six population groups was attributed to genetic differentiation among populations, within populations (among accessions) and within accessions, respectively. Based on the proportion of F_ST_ estimation, divergence between population groups (59%) was higher compared to that estimated among accessions within populations (41%). Among the five wild population groups, the divergence was maximum between POP V and POP VI (F_ST_ = 0.80) and minimum between POP II and POP III (0.23). Higher genetic differentiation between population groups than that within populations based on AMOVA and F_ST_ in a self-pollinated crop species like chickpea is quite expected.

**Table 2:** Polymorphic potential (represented by diverse statistical measures) detected in six model-based population groups (inferred by population genetic structure analysis) using 478 genic and genomic microsatellite markers and 380 TF gene-derived SNP markers.

Population groups	*Cicer* species	Number of accessions	Genotype number/locus	Allele number/locus	PIC (polymorphism information content)	Gene diversity
**POP I**	*C. arietinum*	12	4.19	2–4 (2.2)	0.14–0.68 (0.15)	0.15–0.75 (0.16)
**POP II**	*C. bijugum*	19	4.3	2–6 (2.6)	0.05–0.78 (0.25)	0.05–0.83 (0.27)
**POP III**	*C. echinospermum*	8	5.06	2–5 (2.5)	0.11–0.77 (0.24)	0.12–0.82 (0.26)
**POP IV**	*C. pinnatifidum*	16	4.54	2–6 (2.6)	0.06–0.78 (0.26)	0.06–0.83 (0.29)
**POP V**	*C. reticulatum*	16	5.48	2–8 (3.3)	0.05–0.86 (0.37)	0.06–0.89 (0.42)
	*C. microphyllum*	1				
**POP VI**	*C. judaicum*	22	4.5	2–7 (3.0)	0.04–0.82 (0.32)	0.05–0.87 (0.35)
**Total**		**94**	**4.45**	**2–13 (4.3)**	**0.02–0.98 (0.46)**	**0.22–0.99 (0.50)**

All the 94 cultivated and wild *Cicer* accessions used in this study clearly belonged to a structured population in which about 95% of their inferred ancestry was derived from one of the model-based population and remaining ∼5% contained admixed ancestry **(Table S1)**. Maximum admixed ancestry (∼13%) was observed in POP V, followed by POP II (∼10%) and minimum in POP I (∼0.1%). The POP V had highest admixed ancestry because of their maximum allelic admixtures with POP III (∼6.8%) and POP I (∼3.7%) **(** **Figure 3** **)**. Likewise, we observed maximum admixtures of POP IV (∼9.1%) with POP II and thus its higher admixed ancestry in POP II is expected.

### Estimation of genome-wide and population-specific LD patterns in wild *Cicer* species

**Figure 5:**
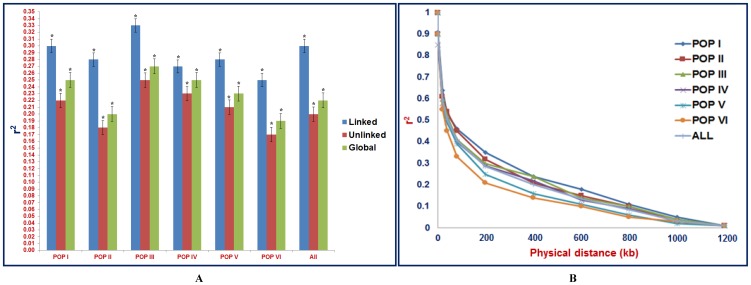
Microsatellite and SNP marker-based estimation of genome-wide and population-specific LD patterns in wild chickpea. (A) Estimates of LD (mean r^2^) for linked, unlinked and global markers (478 genic and genomic microsatellite markers and 380 TF gene-based SNP markers). The bar indicates the standard error. *ANOVA significance at p<0.01. (B) LD decay (mean r^2^) in six population groups as defined by population genetic structure. For LD decay, the r^2^ value of marker physical distance of 0 kb is considered as 1. The marked dots indicate the mean r^2^ values for marker intervals of 0–200, 200–400, 400–600, 600–800, 800–1000 and 1000–1200 kb, respectively. The curve was drawn across the dots using non-linear regression model. “All” includes the LD estimates and decay across entire six population groups.

The LD estimates (r^2^) and extent of LD decay using all possible pair-combination of genome-wide well distributed (physically mapped) 858 markers on eight chickpea chromosomes was determined among 94 *Cicer* accessions belonging to six population groups. In the association panel and whole population groups, average r^2^ of global markers varied from 0.19 in POP VI to 0.27 in POP III (mean: 0.22) **(** **Figure 5A** **)** and 17% global marker-pairs exhibited significant LD (P<0.001) **(Table S7)**, indicating the existence of moderate LD level in the panel of cultivated and wild accessions. Remarkably, 43% and 14% of the linked and unlinked marker-pairs revealed significant LD (P<0.001) and average r^2^ of linked and unlinked markers were estimated as 0.30 and 0.20, respectively. However, the trend of LD estimates and significant LD (%) observed among six population groups using global markers remained alike with that of linked and unlinked marker-pairs. The determination of LD estimates and significant LD (%) using linked marker-pairs that are physically mapped on eight chickpea chromosomes indicated maximum estimates of average r^2^ on chromosome 7 (0.40) and significant LD on chromosome 4 (99.4%) **(Table S7)**. In contrast to unlinked markers, the linked markers detected higher LD estimates and proportion of significant LD in all six population groups (94 accessions) and also across eight chickpea chromosomes. Further, the LD decay of 858 marker-pairs by pooling the r^2^ estimates across eight chromosomes and plotting their average r^2^ against the physical distance of equal intervals (100 kb) up to 1200 kb was determined across six population groups. The non-linear regression curve exhibited a decreasing trend of LD decay with increase in the physical distance (kb) in all the six populations **(** **Figure 5B** **)**. Overall, all the population groups sustained a significant level of LD up to a physical distance of 600 kb. In population groups POP I, POP II and POP III, the LD did not decay below r^2^ = 0.1 up to 800 kb physical distance. However, the genetically more diverse population groups POP IV, POP V and POP VI showed faster LD decay (500–600 kb) than that of less diverse populations POP I, POP II and POP III **(** **Figure 5B** **)**.

## Discussion

Large-scale validation and high-throughput genotyping of sequence-based microsatellite and SNP markers and their utilization in realistic estimate of genetic diversity pattern, population genetic structure and phylogenetic relationships among wild and cultivated *Cicer* accessions is essential for enhancing the genetic potential of cultivated chickpea. Natural genetic variation scanned in the wild *Cicer*, which is expected to be much wider than cultivated species [10], can be transferred from the diverse gene pools of wild accessions to cultivated species to facilitate their genetic improvement through marker-assisted introgression breeding. To accomplish these, microsatellite markers distributed over eight linkage groups of chickpea based on their genetic positions (cM) have been utilized previously for determining the genetic structure and molecular diversity particularly in wild *Cicer* accessions [40]. However, the diversity analysis by selecting markers based on their genetic positions (cM) on population-specific genetic linkage maps usually suffers from poor genome saturation primarily because of low representation of such markers in the chromosomal regions with low recombinant frequency and their population-specific variation of recombination rates. To overcome this constraint, the marker sets showing genome-wide distribution based on physical positions (bp) rather than genetic positions (cM) on entire chromosomes have been utilized for large-scale genotyping applications including genetic diversity studies and genome mapping in crop plants [57]. The genome-wide non-uniform recombination rate is mostly dependent upon the magnitude and characteristics of accessions included under a diversity panel and their level of molecular diversity. It thus infers that the markers selection from constituted LD and haplotype blocks in contrast to physical map of chromosomes is more relevant and informative concerning the efficient marker-based genetic analysis in crop plants [58], [59]. Recently, the implication of selecting markers from LD and haplotype blocks of chromosomes and their effective use in identification of untapped allelic variants and genetic combinations of complex traits in a larger natural population along with capturing a wide spectrum of allelic diversity at whole genome-scale have been well demonstrated in HapMap projects of many crop plants, including maize, rice, soybean and *Medicago* (http://www.medicagohapmap.org) [58], [60], [61]. In this context, we utilized genome-wide well distributed 343 genic and 153 genomic microsatellite markers and 384 TF gene-based SNP markers that are physically mapped on eight chickpea chromosomes (possibly constituting LD/haplotype blocks) for their large-scale validation and high-throughput genotyping in 94 cultivated and wild *Cicer* accessions (belonging to six annual and one perennial *Cicer* species) using gel-based assay, fluorescent dye-labelled automated fragment analyser and MALDI-TOF Sequenom mass array.

The genotyping success rate and intra- and inter-specific polymorphic potential (96.4%) detected by genic and genomic microsatellite markers among 94 *Cicer* accessions is much higher than that estimated earlier in annual and perennial wild accessions (∼40 accessions) using a smaller set of genomic microsatellite markers (30–40% [33], [34], 63% [35], 71% [36], 88.9% [38], 47.6% [39]). The genotyping success rate (98.9%) obtained using MALDI-TOF mass array is higher as compared to that estimated in chickpea using KASPar (competitive allele-specific PCR) (81%) [62] and Illumina GoldenGate (91%) [63] assays, but comparable to Illumina VeraCode-based BeadXpress assay (98%) [64]. Even though the MALDI-TOF assay showed comparable SNP genotyping success rate, its major limitation of lower marker multiplexing potential (∼40 SNP-plex) in contrast to other available high-throughput SNP genotyping assays like GoldenGate and KASPar (hundreds to millions of SNPs multiplexing) has impeded the usage of MALDI-TOF assay in large-scale validation and high-throughput genotyping of SNP loci in crop plants. With regard to small-scale (about hundreds) validation and genotyping of SNPs in a limited number of accessions, however the MALDI-TOF mass array has certain advantages over other high-throughput SNP genotyping assays in terms of cost and time required for its multiplex primer (UEP and EP) designing/synthesis and assaying. These validated microsatellite and SNP markers being derived mostly from the diverse functionally relevant sequence components of genes (TFs) could be useful as functional markers for large-scale genotyping applications including identification of genes/QTLs for many qualitative and quantitative traits of agricultural importance specifically in wild chickpea. The utility of TF genes for regulating diverse growth and developmental processes and stress responses in plant species and legumes have been well studied [65]–[67].

The inter- and intra-specific polymorphism detected by validated 478 microsatellite and 380 SNP markers (total alleles numbers: 3703, average 4.26 alleles/locus, PIC: 0.56 and gene diversity: 0.60) among 94 *Cicer* accessions is higher to that obtained earlier using genomic microsatellite markers (average 5 alleles/locus with PIC: 0.26 [35], 3.3 alleles/locus and PIC 0.48 [39], 6 alleles/locus with PIC: 0.68, [38] 5 alleles/locus with PIC: 0.47 [45]) and genic microsatellite, intron-spanning and SNP markers (PIC: 0.34 [37]). Higher heterozygosity (0.02) among 82 accessions belonging to six wild *Cicer* species than that of cultivated species (0.0) and positive correlation of both PIC and gene diversity with number of alleles/locus are in line with the findings of Upadhyaya et al. [40]. The cloning and sequencing of size variant amplicons of selected microsatellite marker sets from the *Cicer* accessions confirms that the allelic variation estimated in this study across diverse wild and cultivated species was due to variation in their microsatellite repeat-length, but not because of size homoplasy (insertions/deletions in the sequences flanking the repeat-motifs) of microsatellite markers as expected during inter-specific polymorphism [68], [69]. A larger set of 478 genic and genomic microsatellite markers (showing no significant homoplasy at inter-species level) and 380 TF gene-based SNP markers covering the whole genome selected in this study through their validation and genotyping in 94 cultivated and wild *Cicer* accessions would thus have relevance for assessing the polymorphic potential and understanding the diversity pattern, population genetic structure and phylogenetics in chickpea.

A wide range (11 to 94%, mean: 62%) of genetic diversity observed in this study among 94 cultivated and wild *Cicer* accessions using genome-wide physically mapped 478 genic and genomic microsatellite markers and 380 TF-gene-based SNP markers is higher than the molecular diversity level detected previously with the EST-derived (3 to 49% [68]) and genomic (37 to 80% [34], 32 to 80% [36], 6 to 84% [39] and 34 to 88% [38]) microsatellite markers. In spite of high mutation rate of microsatellite markers in contrast to SNP markers across diverse species, the high linear tendency correlation (80%) between genetic distances detected by both these markers suggests no saturation of microsatellite markers at which the genetic distances are being measured. Therefore, the combined use of microsatellite and SNP markers in estimation of efficient and precise molecular diversity in 94 *Cicer* accessions is relevant. The genic microsatellite and SNP markers showing high inter-specific polymorphism (98.2%) and wider genetic diversity (13 to 78%, mean: 58%) specifically among wild and cultivated accessions could be utilized for precise identification of true inter-specific hybrids. Besides, such markers could also have utility in monitoring the introgression breeding to access the transfer of target genic regions controlling the traits of agricultural importance including abiotic and biotic stress tolerance from wild relatives to the backgrounds of cultivated gene pool for their genetic improvement. Henceforth, a wider genetic base and functional molecular diversity detected by genic markers in the expressed sequence component of genome among cultivated and wild accessions would be of significance in selection of useful diverse parental cultivated and wild accessions in cross-breeding program for the perspective of chickpea varietal improvement. Remarkably, seven microsatellite and two SNP markers were identified as most informative that could differentiate all 94 accessions representing seven cultivated and wild *Cicer* species from each other. Therefore, these smaller set of microsatellite and SNP markers selected by us might assay more relevant regions of chickpea genome for establishing distinctness among accessions.

The present study have made use of unrooted tree to decipher the phylogenetic relationships among 94 cultivated and wild chickpea accessions. The use of unrooted tree as a reliable approach for explaining evolutionary relationships among diverse crop genotypes has extensively been reported [60], [61]. Correspondence of genetic relationships established among 94 cultivated and wild *Cicer* accessions using both microsatellite and SNP markers with their species and gene pools of origination than that of individual microsatellite and SNP markers suggested that the functional genetic diversity assayed in our study by combining both kinds of sequence-based markers is realistic and thus would be useful in chickpea genomics and breeding when applied to a larger set of contrasting accessions. The converse genetic attributes of microsatellite and SNP markers specifically in terms of their high/low mutation rate, multi-/bi-allelic nature and abundance in genomic distribution might have complemented the level of polymorphic potential detected by each of these two marker system in 94 accessions with each other to produce a realistic estimate of molecular diversity in the chickpea genome. It is clearly evident from high degree of genetic distance-based linear correlation (∼80%) between microsatellite and SNP markers as also have been observed in previous studies of many crop plants including maize and rye [70], [71].

The inclusion of accessions belonging to *C. arietinum*, *C. reticulatum* and *C. echinospermum* species into three different population groups (determined by population genetic structure) as members of primary gene pool and accessions representing from *C. bijugum*, *C. pinnatifidum* and *C. judaicum* species under secondary gene pool as three diverse populations was clearly evident. These observation are fairly comparable with earlier genetic diversity, population structure and phylogenetic relationship studies on the cultivated and wild *Cicer* accessions using morphological traits, inter-specific hybridization, karyotyping, seed storage protein and isozyme profiling [13]–[24], [72] as well as random and sequence-based molecular markers [25]–[34], [36]–[40], [42], [73], [74], but with some remarkable deviations. The mean F_ST_ among three wild species accessions of primary gene pool was estimated to be 0.55, whereas it was 0.60 between primary and secondary gene pools. Thus, the evaluated F_ST_ is in line with expected evolutionary relationships among the cultivated and wild species accessions belonging to primary and secondary gene pools. In most of these studies, no distinct differentiation/groupings among the accessions belonging to *Cicer* species (*C. arietinum*, *C. reticulatum* and *C. echinospermum*) of primary gene pools and also no collinear conclusive findings on origination of accessions representing *C. bijugum* and *C. pinnatifidum* either from primary or secondary gene pools was observed. In this context, our observation on distinct differentiation of all accessions included under six different annual/perennial *Cicer* species into six population groups based on their species/gene pools of origination is relevant. It could be due to combined use of genome-wide well distributed genic and genomic microsatellite and SNP markers that are physically mapped on eight chickpea chromosomes and thus might provide realistic estimate of diversity pattern and population structure among wild and cultivated *Cicer* accessions.

The existence of about 5% admix ancestry among six population groups suggests that evolutionary divergence of all six cultivated and wild chickpea was from a common ancestor. It further concurs with the earlier reports on origination, diversification and domestication of early Neolithic chickpea along with founder crops in the Fertile Crescent (eastern Mediterranean region) about 10,000–12,000 years ago [1], [75]–[77]. Subsequently, complicated domestication pattern and complex breeding history involving inter-crossing/introgression coupled with different strong adaptive selection pressure among wild and cultivated chickpea accessions have led to diverse admixture frequency across six population groups. Maximum admixtures among the *Cicer* species belonging to POP I (*C. arietinum*), POP III (*C. echinospermum*) and POP V (*C. reticulatum*) as members of primary gene pools indicate that their evolutionary closeness could be due to their domestication at the archaeological sites of South Eastern Turkey about 10,000 years ago [1], [72]–[74]. It is supported well with commonly accepted presumptions of previous molecular studies [13], [29], [30], [33], [34], [36]–[39], [40], [42]. In spite of close phylogenetic relationship of more diverse *C. reticulatum* accessions under POP V with accessions of *C. echinospermum* (POP III) and *C. arietinum* (POP I), we observed low genetic variability among cultivated *desi* and *kabuli* accessions of *C. arientinum*. It could be due to maximum adaptation-based selection pressure during the domestication of cultivated *C. arientinum* from wild *C. reticulatum*, followed by four sequential evolutionary bottlenecks which in turn narrow down the genetic base in cultivated chickpea [1], [2], [5]. A very limited eco-geographic distribution and narrow adaptation of wild progenitor accessions belonging to primary and secondary gene pools in south-eastern Turkey [78] and combined impacts of many factors associated with their domestication pattern, including founder effect in the ancient Neolithic era, separation of reproductive phases during early Bronze age, adaption-based selection (against vernalization response) for a wide-range of agro-ecological niches and modern breeding efforts might have caused low introgressive gene flow from wild to cultivated gene pools [3]. It ultimately resulted in narrow genetic base in cultivated *desi* and *kabuli* population as compared to other wild population groups. The estimation of higher genetic diversity particularly among the wild species/accessions originated from Fertile Crescent than that of cultivated accessions from Central Asia supported these findings. A maximum admixture (∼9.1%) between POP II (*C. bijugum*) and POP IV (*C. pinnatifidum*) is expected as they both belong to secondary gene pools. Remarkably, population genetic structure of a perennial wild accession *C. microphyllum* included under POP V (*C. reticulatum*) contained more admixed ancestry from *C. arietinum* (POP I), *C. reticulatum* (POP V), *C. echinospermum* (POP III) of primary gene pools rather than *C. pinnatifidum* (POP IV) of secondary gene pools. The perennial *C. microphyllum* accession used in our study is of Indian origin and thus showed higher admixtures with Indian originated POP I (*C. arietinum*) and its two other evolutionarily closely related population groups (POP III and POP V) of primary gene pools. The diversity, population genetic structure, domestication and phylogenetics-related information generated in this study for 94 wild and cultivated *Cicer* accessions have implications for many genotyping applications including association analysis and genetic/QTL mapping targeting different qualitative and quantitative traits of agricultural importance in chickpea.

Higher LD estimates and proportion of significant LD obtained in all six population groups (comprising of 94 accessions) and also across eight chickpea chromosomes using the linked markers in contrast to unlinked markers reflected the direct correlation of LD patterning with physical linkage of markers on chromosomes and marker density required to cover the genomic regions. The extensive LD estimates and extended chromosomal LD decay (∼500–600 kb) determined in the six population groups using the microsatellite and SNP markers were much higher than that reported previously in cross-pollinated [58], [79]–[83] and non-domesticated [84], [85] self-pollinated plant species, but comparable to domesticated selfing crop species like barley [86], [87]. The extensive LD estimates and extended LD decay in the domesticated self-pollinated crop species like chickpea in contrast to other domesticated self-pollinated crops like rice and soybean could be due to extensive contribution of four sequential bottlenecks during the domestication of chickpea [1], [3]–[5], which resulted in the reduction of genetic diversity in cultivated chickpea than that of other domesticated selfing crop plant species. Slower LD decay of more diverse population groups (POP II–VI) as compared to POP I representing the cultivated *desi* and *kabuli* accessions of *C. arietinum* is expected because of four sequential evolutionary bottlenecks occurring during their evolutionary divergence and domestication with *C. reticulatum* crop wild relative [4], [5], [77]. Remarkably, a varied LD decay was observed in six population groups even by combined use of higher number of genic and genomic microsatellite and SNP markers distributed across eight chromosomes. It suggests the effect of factors other than marker-density including genetic diversity and population genetic structure on shaping the LD patterns [86] specifically in wild *Cicer* accessions. Overall, we inferred the population-specific LD patterns by considering the genome-wide recombination rates in 94 cultivated and wild *Cicer* accessions. It gave us an insight about the emerging strategies that have been utilized in HapMap projects of many crop plants [58], [60], [61] for precise natural allelic diversity estimation as well as efficient trait association analysis and genetic/QTL mapping.

## Conclusions

High genotyping success rate and intra-/and inter-specific polymorphic potential (97.5%) was detected by large-scale validation of genome-wide physically mapped 478 genic and genomic microsatellite markers and 380 TF gene-derived SNP markers in 94 annual/perennial *Cicer* species using gel-based assay, fluorescent dye labelled automated fragment analyser and MALDI-TOF mass array. A wider genetic base (13–78%) and thus a higher natural allelic diversity specifically in the functionally relevant genic regions of wild chickpea was unraveled suggesting their utility as potential resource for trait enhancement of cultivated gene pool. The informative 858 mapped markers thus have potential for selecting divergent cultivated and wild accessions and identifying true inter-specific hybrids for successful introgression breeding program, which will then facilitate the transfer of target genomic/gene regions controlling important agronomic traits from wild to cultivated chickpea for their genetic improvement. Wider genetic base, distinct species/gene pool-wise differentiation, admixed domestication pattern, differential genome-wide recombination, extended LD decay (500–600 kb) and extensive LD estimates (0.19–0.27) in a six structured wild and cultivated population were observed. The molecular diversity and LD-based information generated in our study considering the genome-wide recombination rate in six diverse wild and cultivated population gave clues to select informative markers for precise allelic diversity estimation and marker-based large-scale genotyping applications in chickpea. It will further help us to identify natural targets for chickpea genomics-assisted breeding by deriving the causal impact of natural selection on adaptation of diverse wild and cultivated chickpea populations that are domesticated across various agro-climatic regions.

## Supporting Information

Figure S1 **A representative 34-plex extension mass spectra for 94 cultivated and wild** ***Cicer*** **accessions obtained through MALDI-TOF mass array genotyping assay.** The SNP IDs and their corresponding SNP loci are indicated on the top of each spectra.
(PDF)

Figure S2 **Functional annotation of informative 334 microsatellite and 380 SNP markers validated in the genes showed maximum correspondence to transcription factor gene families (73.5%), followed by genes controlling growth and metabolism enzymes (23.8%) and expressed proteins (2.7%).**
(PDF)

Figure S3 **The sequencing of cloned amplicons from a microsatellite marker showing fragment length polymorphism (156 and 162 bp) among cultivated and wild accessions, validated the presence of expected repeat-motifs (TTG)_n_ and further corresponded with their expansion/contraction [(TTG)_9_/(TTG)_11_] of number of repeat-units.**
(PDF)

Figure S4 **Correlation between genetic distances detected by microsatellite and SNP markers among 94 accessions belonging to seven wild and cultivated species.** Each blue colored dot represents the genetic distance between a pair of accessions based on allele sharing of microsatellite (y-axis) and SNP markers (x-axis).
(PDF)

Figure S5 **Unrooted phylogenetic tree depicting the genetic relationships among 94 cultivated and wild accessions belonging to seven** ***Cicer*** **species based on Nei's genetic distance using 380 TF gene-derived SNP markers.** Molecular classification is not able to differentiate accessions into six different clusters as expected based on their species and gene pools of origination.
(PDF)

Figure S6 **Unrooted phylogenetic tree depicting the genetic relationships among 94 cultivated and wild accessions belonging to seven** ***Cicer*** **species based on Nei's genetic distance using 478 microsatellite markers (including 334 genic and 144 genomic microsatellite markers).** Molecular classification clearly differentiated 94 accessions into six different clusters, which corresponds to their species and gene pools of origination with deviations.
(PDF)

Figure S7 **Optimization of number of populations (K value) varying from K = 1 to 10 to determine best possible population number for 94 cultivated and wild** ***Cicer*** **accessions using the** ***ad hoc*** **procedure (A) of STRUCTURE documented by Pritchard et al. (2000) and second order statistics (delta K) (B) of Evanno et al. (2005).**
(PDF)

Table S1 **Wild and cultivated** ***Cicer*** **accessions used in the study and their inferred ancestry coefficients in population genetic structure analysis.**
(PDF)

Table S2 **Summary of 496 including 343 genic and 153 genomic microsatellite markers genotyped in 94 annual/perennial cultivated and wild** ***Cicer*** **accessions using gel-based assay and fluorescent dye labelled automated fragment analyzer.**
(PDF)

Table S3 **Summary of 384 TF gene-derived SNPs genotyped in 94 annual/perennial cultivated and wild** ***Cicer*** **accessions using high-throughput MALDI-TOF mass array.**
(PDF)

Table S4 **Polymorphic potential (represented by diverse statistical measures) detected by 478 genic and genomic microsatellite markers and 380 TF gene-derived SNP markers among 94 cultivated and wild** ***Cicer*** **accessions.**
(PDF)

Table S5 **Molecular diversity (represented by different diversity statistical measures) detected by 478 genic and genomic microsatellite markers and 380 TF gene-derived SNP markers among 94 cultivated and wild accessions within and between** ***Cicer*** **species.**
(PDF)

Table S6 **Pair-wise estimates of genetic divergence (F_ST_) and genetic distance among six model-based population groups.**
(PDF)

Table S7 **LD estimates in six model-based individual population groups and entire population as well as across eight chromosomes.**
(PDF)
