# Supplementary material for: Natural Allelic Diversity, Genetic Structure and Linkage Disequilibrium Pattern in Wild Chickpea
Source: PLoS One. 2014 Sep 15;9(9):e107484. doi: 10.1371/journal.pone.0107484 (PMC4164632; doi:10.1371/journal.pone.0107484)
Supplement: Figure S1 — A representative 34-plex extension mass spectra for 94 cultivated and wild Cicer accessions obtained through MALDI-TOF mass array genotyping assay. The SNP IDs and their corresponding SNP loci are indicated on the top of each spectra. (PDF) [file pone.0107484.s001.pdf]

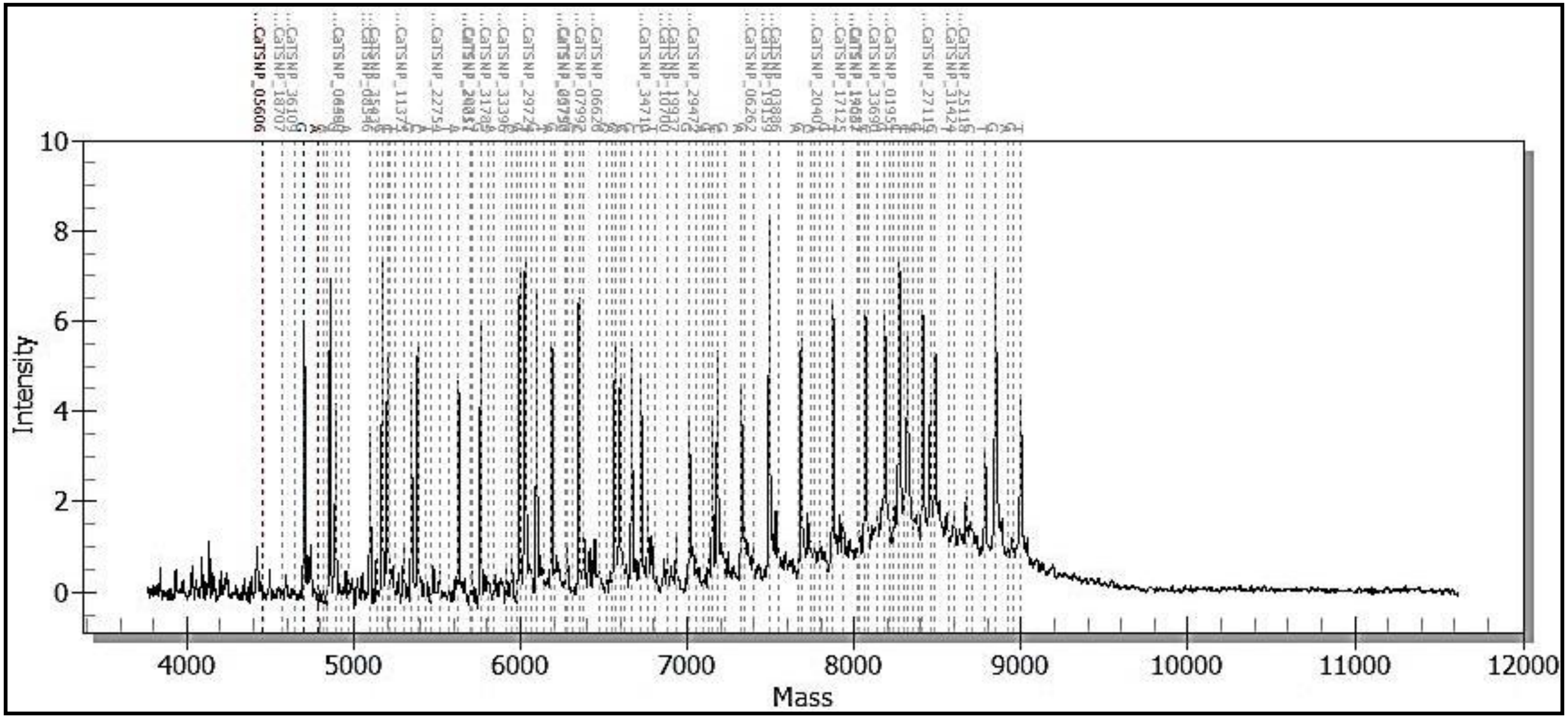

Figure S1: A representative 34-plex extension mass spectra for 94 cultivated and wild *Cicer* accessions obtained through MALDI-TOF mass array genotyping assay. The SNP IDs and their corresponding SNP loci are indicated on the top of each spectra.
