# Supplementary material for: Natural Allelic Diversity, Genetic Structure and Linkage Disequilibrium Pattern in Wild Chickpea
Source: PLoS One. 2014 Sep 15;9(9):e107484. doi: 10.1371/journal.pone.0107484 (PMC4164632; doi:10.1371/journal.pone.0107484)
Supplement: Figure S2 — Functional annotation of informative 334 microsatellite and 380 SNP markers validated in the genes showed maximum correspondence to transcription factor gene families (73.5%), followed by genes controlling growth and metabolism enzymes (23.8%) and expressed proteins (2.7%). (PDF) [file pone.0107484.s002.pdf]

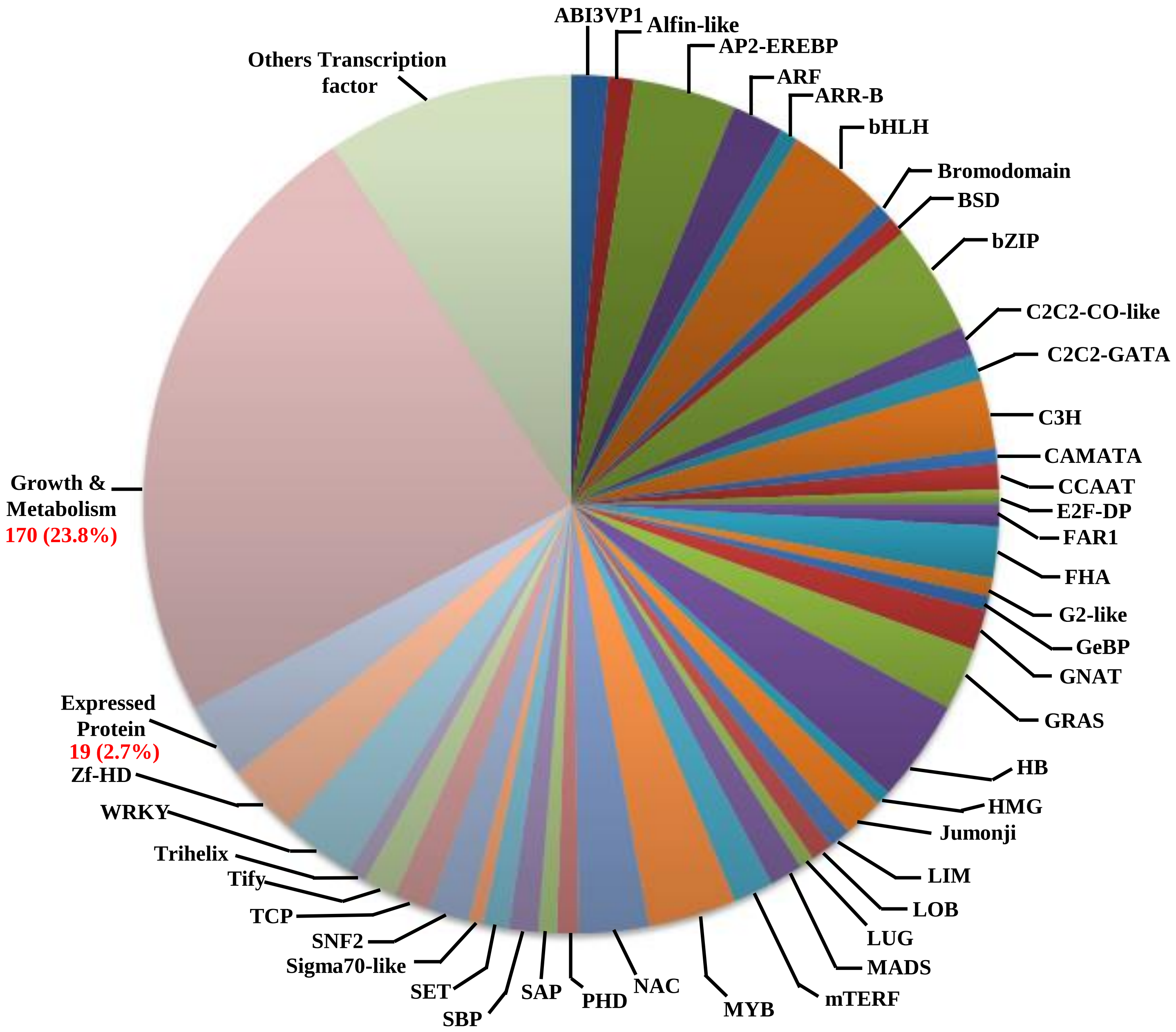

Figure S2: Functional annotation of informative 334 microsatellite and 380 SNP markers validated in the genes showed maximum correspondence to transcription factor gene families (73.5%), followed by genes controlling growth and metabolism enzymes (23.8%) and expressed proteins (2.7%)
