# Supplementary figures and images for: Natural Allelic Diversity, Genetic Structure and Linkage Disequilibrium Pattern in Wild Chickpea
Source: PLoS One. 2014 Sep 15;9(9):e107484. doi: 10.1371/journal.pone.0107484 (PMC4164632; doi:10.1371/journal.pone.0107484)

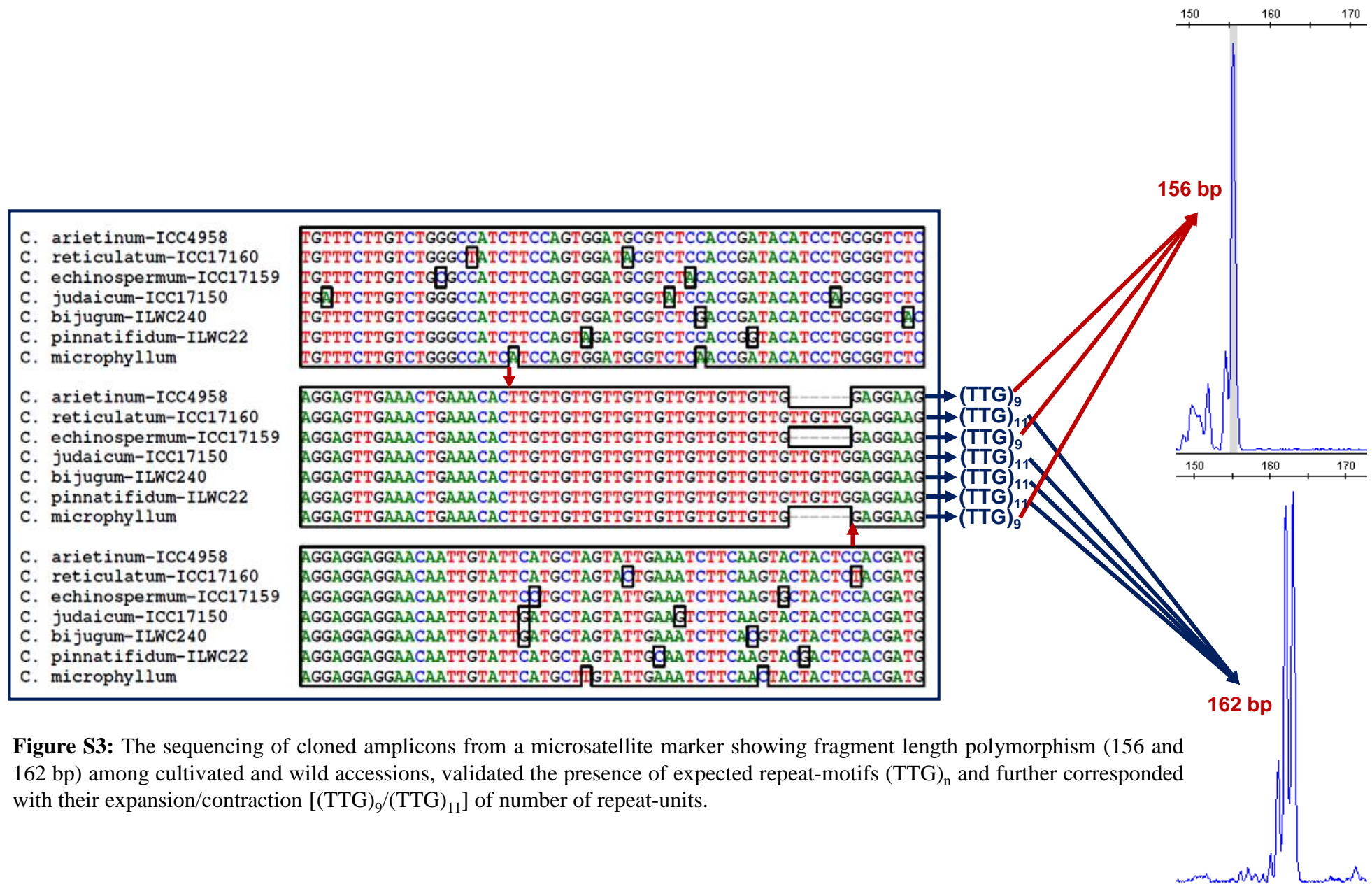

Supplement: Figure S3 — The sequencing of cloned amplicons from a microsatellite marker showing fragment length polymorphism (156 and 162 bp) among cultivated and wild accessions, validated the presence of expected repeat-motifs (TTG)n and further corresponded with their expansion/contraction [(TTG)9/(TTG)11] of number of repeat-units. (PDF) [file pone.0107484.s003.pdf]
