# Supplementary material for: Natural Allelic Diversity, Genetic Structure and Linkage Disequilibrium Pattern in Wild Chickpea
Source: PLoS One. 2014 Sep 15;9(9):e107484. doi: 10.1371/journal.pone.0107484 (PMC4164632; doi:10.1371/journal.pone.0107484)
Supplement: Figure S4 — Correlation between genetic distances detected by microsatellite and SNP markers among 94 accessions belonging to seven wild and cultivated species. Each blue colored dot represents the genetic distance between a pair of accessions based on allele sharing of microsatellite (y-axis) and SNP markers (x-axis). (PDF) [file pone.0107484.s004.pdf]

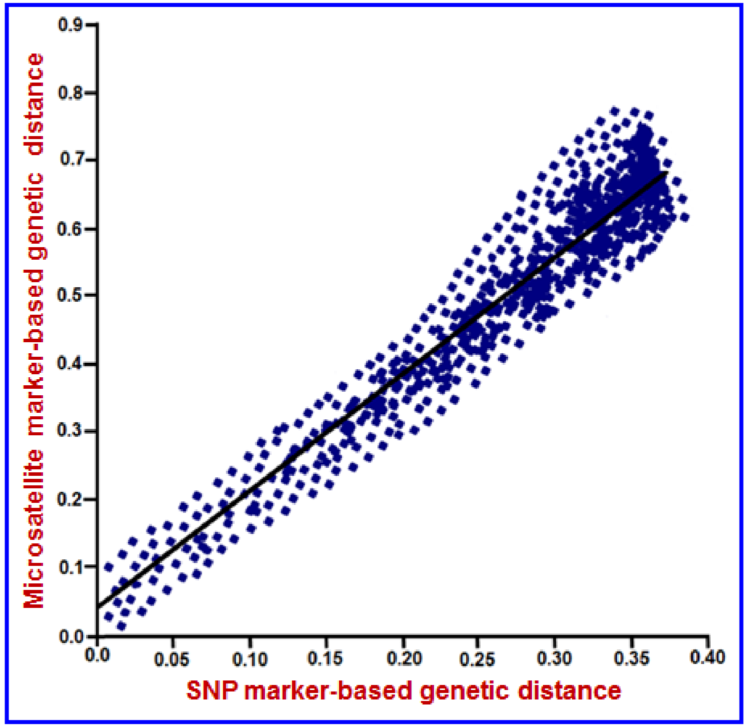

Figure S4: Correlation between genetic distances detected by microsatellite and SNP markers among 94 accessions belonging to seven wild and cultivated species. Each blue colored dot represents the genetic distance between a pair of accessions based on allele sharing of microsatellite (y-axis) and SNP markers (x-axis).
