# Supplementary material for: Natural Allelic Diversity, Genetic Structure and Linkage Disequilibrium Pattern in Wild Chickpea
Source: PLoS One. 2014 Sep 15;9(9):e107484. doi: 10.1371/journal.pone.0107484 (PMC4164632; doi:10.1371/journal.pone.0107484)
Supplement: Figure S5 — Unrooted phylogenetic tree depicting the genetic relationships among 94 cultivated and wild accessions belonging to seven Cicer species based on Nei's genetic distance using 380 TF gene-derived SNP markers. Molecular classification is not able to differentiate accessions into six different clusters as expected based on their species and gene pools of origination. (PDF) [file pone.0107484.s005.pdf]

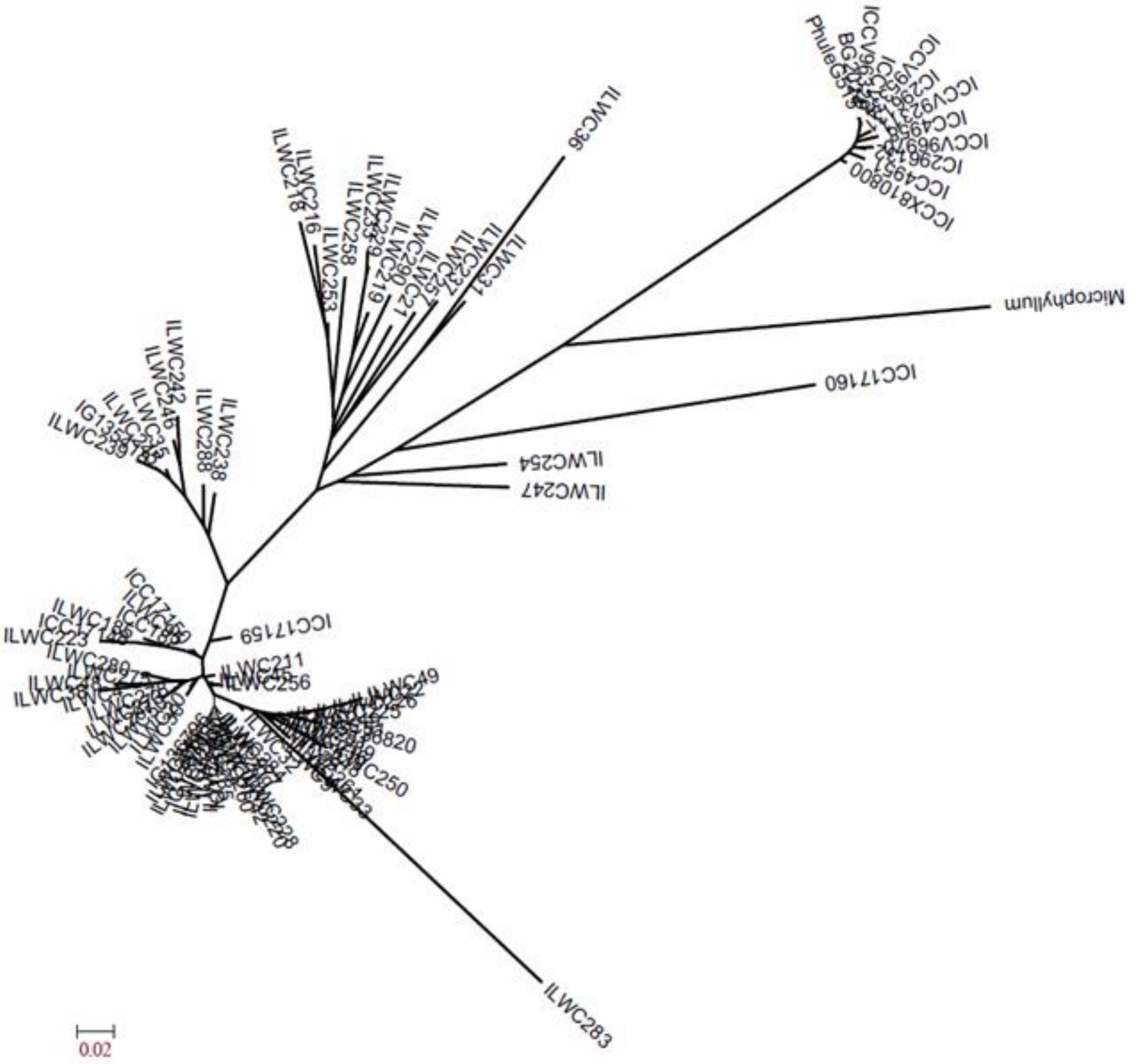

Figure S5: Unrooted phylogenetic tree depicting the genetic relationships among 94 cultivated and wild accessions belonging to seven *Cicer* species based on Nei's genetic distance using 380 TF gene-derived SNP markers. Molecular classification is not able to differentiate accessions into six different clusters as expected based on their species and gene pools of origination.
