# Supplementary material for: Natural Allelic Diversity, Genetic Structure and Linkage Disequilibrium Pattern in Wild Chickpea
Source: PLoS One. 2014 Sep 15;9(9):e107484. doi: 10.1371/journal.pone.0107484 (PMC4164632; doi:10.1371/journal.pone.0107484)
Supplement: Figure S7 — Optimization of number of populations (K value) varying from K = 1 to 10 to determine best possible population number for 94 cultivated and wild Cicer accessions using the ad hoc procedure (A) of STRUCTURE documented by Pritchard et al. (2000) and second order statistics (delta K) (B) of Evanno et al. (2005). (PDF) [file pone.0107484.s007.pdf]

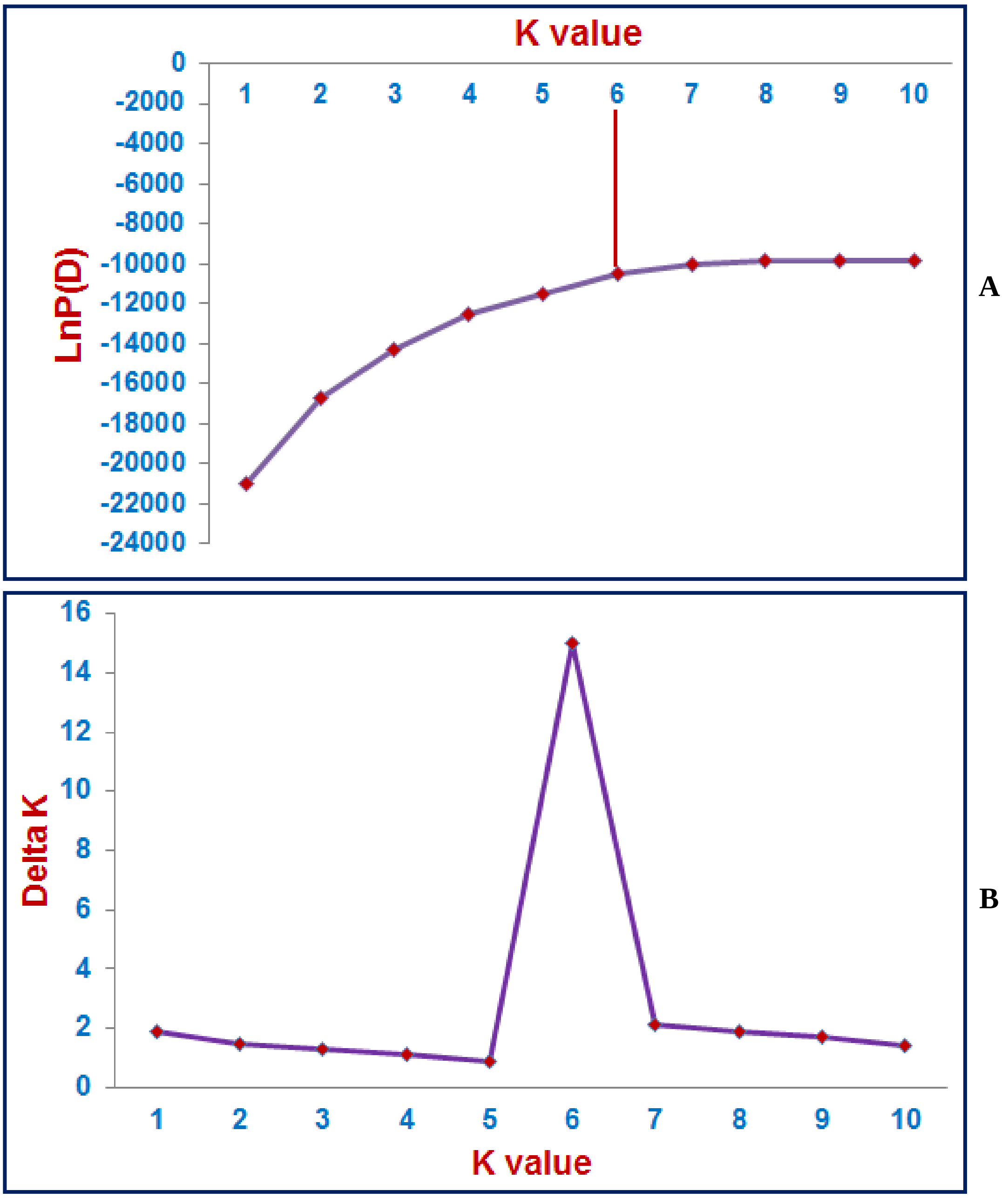

Figure S7: Optimization of number of populations (K value) varying from K = 1 to 10 to determine best possible population number for 94 cultivated and wild *Cicer* accessions using the *ad hoc* procedure (A) of STRUCTURE documented by Pritchard et al. (2000) and second order statistics (delta K) (B) of Evanno et al. (2005).
