# Supplementary material for: Natural Allelic Diversity, Genetic Structure and Linkage Disequilibrium Pattern in Wild Chickpea
Source: PLoS One. 2014 Sep 15;9(9):e107484. doi: 10.1371/journal.pone.0107484 (PMC4164632; doi:10.1371/journal.pone.0107484)
Supplement: Table S1 — Wild and cultivated Cicer accessions used in the study and their inferred ancestry coefficients in population genetic structure analysis. (PDF) [file pone.0107484.s008.pdf]

Table S1: Wild and cultivated *Cicer* accessions used in the study and their inferred ancestry coefficients in population genetic structure analysis

Sl. No.	*Cicer* species	Accession No.	Cultivar types	Geographical origin	Inferred ancestry among *Cicer* accessions					
					Population I (POP I)	Population II (POP II)	Population III (POP III)	Population IV (POP IV)	Population V (POP V)	Population VI (POP VI)
1	***C. arietinum* (annual cultivated)**	ICCX810800	*desi*	India	**0.999**	0	0	0	0	0
2		IC296132	*desi*	India	**0.999**	0	0	0	0	0
3		ICCV96970	*desi*	India	**0.999**	0	0	0	0	0
4		IC296131	*desi*	India	**0.999**	0	0	0	0	0
5		ICC4958	*desi*	India	**0.998**	0	0	0	0.001	0
6		ICC4951	*desi*	India	**0.999**	0	0	0	0	0
7		ICCV2	*kabuli*	India	**0.999**	0	0	0	0	0
8		ICCV92311	*kabuli*	India	**0.999**	0	0	0	0	0
9		ICCV95334	*kabuli*	India	**0.999**	0	0	0	0	0
10		ICCV96329	*kabuli*	India	**0.999**	0	0	0	0	0
11		BG2024	*kabuli*	India	**0.999**	0	0	0	0	0
12		PhuleG515	*kabuli*	India	**0.999**	0	0	0	0	0
13	***C. reticulatum* (annual wild)**	ICC17160	Wild	Turkey	0.025	0	0	0.008	**0.966**	0
14		ILWC290	Wild	Turkey	0.001	0.001	0.005	0.001	**0.99**	0.001
15		ILWC233	Wild	Turkey	0.001	0	0.001	0	**0.998**	0
16		ILWC253	Wild	Turkey	0	0	0	0	**0.998**	0
17		ILWC254	Wild	Turkey	0.098	0.001	0.028	0.009	**0.859**	0.005
18		ILWC237	Wild	Turkey	0.084	0	0.053	0.002	**0.859**	0.001
19		ILWC257	Wild	Turkey	0.001	0.001	0.011	0.001	**0.985**	0.001
20		ILWC218	Wild	Turkey	0	0	0	0	**0.998**	0
21		ILWC247	Wild	Turkey	0.044	0.001	0.001	0.001	**0.947**	0.005
22		ILWC229	Wild	Turkey	0	0	0	0	**0.998**	0
23		ILWC219	Wild	Turkey	0	0	0	0.001	**0.998**	0
24		ILWC242	Wild	Turkey	0	0.002	0.379	0.003	**0.582**	0.033
25		ILWC258	Wild	Turkey	0.107	0.001	0.022	0	**0.867**	0.003
26		ILWC21	Wild	Turkey	0.078	0.001	0.196	0.001	**0.722**	0.001
27		ILWC36	Wild	Turkey	0	0.003	0.141	0.002	**0.853**	0.001
28		ILWC216	Wild	Turkey	0	0	0.235	0	**0.764**	0.001

29	***C. echinospermum* (annual wild)**	ICC17159	Wild	Turkey	0.008	0.09	**0.858**	0.001	0.001	0.042
30		ILWC35	Wild	Turkey	0	0.001	**0.914**	0.083	0	0.001
31		IG135418	Wild	Syrian Arab Republic	0.001	0.006	**0.991**	0.001	0.001	0.001
32		ILWC239	Wild	Turkey	0.001	0	**0.997**	0.001	0	0
33		ILWC245	Wild	Turkey	0	0	**0.998**	0	0	0
34		ILWC238	Wild	Turkey	0	0	**0.999**	0	0	0
35		ILWC246	Wild	Turkey	0	0.003	**0.995**	0	0	0
36		ILWC288	Wild	Turkey	0	0	**0.998**	0	0	0.001
37	***C. judaicum* (annual wild)**	ICC182	Wild	ICRISAT, India	0	0.001	0.001	0.001	0	**0.997**
38		ICC17150	Wild	Lebanon	0	0.001	0.001	0.001	0	**0.997**
39		ILWC95	Wild	ICRISAT, India	0	0.001	0.001	0	0	**0.997**
40		ILWC31	Wild	Jordan	0.001	0.002	0.001	0.001	0.395	**0.601**
41		ILWC185	Wild	ICRISAT, India	0.001	0	0.004	0.001	0	**0.994**
42		ICC17148	Wild	Lebanon	0	0	0.001	0	0	**0.998**
43		ILWC280	Wild	Syrian Arab Republic	0	0	0.001	0	0	**0.998**
44		ILWC283	Wild	Syrian Arab Republic	0.005	0.001	0.001	0.001	0.001	**0.991**
45		ILWC20	Wild	Israel	0	0	0.004	0	0	**0.994**
46		ILWC211	Wild	Syrian Arab Republic	0	0	0.001	0.001	0	**0.997**
47		ILWC30	Wild	Israel	0	0	0.001	0.001	0.001	**0.997**
48		ILWC48	Wild	Syrian Arab Republic	0	0.001	0.001	0.001	0	**0.998**
49		ILWC50	Wild	Syrian Arab Republic	0	0	0	0	0	**0.999**
50		ILWC207	Wild	Syrian Arab Republic	0	0.001	0.001	0.001	0	**0.997**
51		ILWC45	Wild	Syrian Arab Republic	0	0.103	0	0.001	0	**0.895**
52		ILWC38	Wild	Lebanon	0.001	0.001	0	0.001	0.01	**0.986**
53		ILWC278	Wild	Syrian Arab Republic	0	0.001	0	0.001	0	**0.997**
54		ILWC275	Wild	Lebanon	0	0.001	0	0.001	0	**0.997**
55		ILWC223	Wild	Lebanon	0.001	0.01	0.001	0.001	0.001	**0.986**
56		ILWC256	Wild	Jordan	0	0.005	0.001	0.006	0.001	**0.987**
57		ILWC273	Wild	Lebanon	0	0.002	0.001	0.001	0.001	**0.996**
58		ILWC4	Wild	Lebanon	0.001	0.018	0	0.004	0.001	**0.976**

59	***C. bijugum***	ILWC42	Wild	Syrian Arab Republic	0	**0.842**	0.001	0.002	0.001	0.154
60	**(annual wild)**	IG136792	Wild	Syrian Arab Republic	0	**0.998**	0.001	0.001	0	0
61		IG136786	Wild	Syrian Arab Republic	0	**0.997**	0	0.002	0	0
62		ILWC277	Wild	Syrian Arab Republic	0	**0.999**	0	0	0	0
63		ILWC8	Wild	Turkey	0	**0.998**	0	0	0	0
64		ILWC228	Wild	Turkey	0	**0.997**	0	0.001	0.001	0.001
65		ILWC240	Wild	Turkey	0	**0.999**	0	0	0	0
66		ILWC241	Wild	Turkey	0	**0.998**	0	0	0	0
67		ILWC260	Wild	Turkey	0	**0.999**	0	0	0	0
68		ILWC217	Wild	Turkey	0	**0.998**	0	0	0	0
69		ILWC209	Wild	Syrian Arab Republic	0	**0.998**	0	0.001	0	0
70		ILWC227	Wild	Turkey	0	**0.996**	0.002	0.001	0	0.001
71		ILWC284	Wild	Turkey	0	**0.997**	0.001	0.001	0	0.001
72		ILWC7	Wild	Turkey	0	**0.997**	0	0.002	0	0.001
73		ILWC220	Wild	Turkey	0.001	**0.996**	0.001	0.001	0	0.001
74		IG136796	Wild	Syrian Arab Republic	0	**0.78**	0.001	0.218	0	0.001
75		ILWC285	Wild	Turkey	0	**0.579**	0.001	0.407	0	0.012
76		ILWC286	Wild	Turkey	0	**0.486**	0	0.512	0	0.001
77		ILWC32	Wild	Turkey	0	**0.423**	0	0.575	0.001	0.001
78	***C. pinnatifidum***	ILWC9	Wild	Turkey	0	0.001	0	**0.998**	0	0
79	**(annual wild)**	ILWC22	Wild	Turkey	0	0	0	**0.998**	0	0
80		IG136820	Wild	Syrian Arab Republic	0	0	0	**0.998**	0	0
81		ILWC251	Wild	Turkey	0	0.001	0	**0.997**	0.001	0.001
82		ILWC29	Wild	Turkey	0	0.001	0	**0.998**	0	0
83		ILWC33	Wild	Turkey	0	0	0	**0.998**	0	0
84		ILWC49	Wild	Syrian Arab Republic	0	0	0.001	**0.997**	0	0.001
85		ILWC226	Wild	Turkey	0.001	0	0	**0.998**	0	0
86		ILWC248	Wild	Turkey	0	0.001	0.001	**0.998**	0	0.001
87		ILWC249	Wild	Turkey	0	0.009	0.001	**0.99**	0	0
88		ILWC236	Wild	Turkey	0.001	0.001	0	**0.997**	0	0.001
89		ILWC225	Wild	Turkey	0	0	0	**0.998**	0	0
90		ILWC51	Wild	Turkey	0	0	0	**0.998**	0	0.001
91		ILWC250	Wild	Turkey	0	0	0.001	**0.998**	0	0
92		ILWC261	Wild	Syrian Arab Republic	0	0	0.003	**0.985**	0.008	0.003
93		ILWC289	Wild	Turkey	0.001	0.001	0.001	**0.996**	0.001	0
94	***C. microphyllum*** **(perennial wild)**	*microphyllum*	Wild	India	0.192	0.001	0.086	0.294	**0.425**	0.001

Yellow highlights indicate the maximum inferred ancestry coefficient of 94 *Cicer* accessions that led to their correspondence with one model-based population as determined by STRUCTURE
