# Supplementary material for: Natural Allelic Diversity, Genetic Structure and Linkage Disequilibrium Pattern in Wild Chickpea
Source: PLoS One. 2014 Sep 15;9(9):e107484. doi: 10.1371/journal.pone.0107484 (PMC4164632; doi:10.1371/journal.pone.0107484)
Supplement: Table S2 — Summary of 496 including 343 genic and 153 genomic microsatellite markers genotyped in 94 annual/perennial cultivated and wild Cicer accessions using gel-based assay and fluorescent dye labelled automated fragment analyzer. (PDF) [file pone.0107484.s009.pdf]

Table S2: Summary of 496 including 343 genic and 153 genomic microsatellite markers genotyped in 94 annual/perennial cultivated and wild *Cicer* accessions using gel-based assay and fluroscent dye labelled automated fragment analyser

Marker IDs*	Microsatellite repeat-motifs	Forward Primer sequences (5'-3')	Reverse Primer sequences (5'-3')	Actual annealing temperature (0C)	Size (bp) of alleles amplified	Types of markers	Chromosomes	Physical positions (bp)	Putative functions
CaSSR1	(TTA)5	CCTCGCCAAATAATCTCAGG	CCGAAGAGCAGAGGAAGATG	60.1	259	Genic	Ca1	203295	Zinc finger C-x8-C-x5-C-x3-H type
CaSSR2	(TTA)8	TGATGATTATTTCGTTTCCTC	GCAACTAGAAGACGCTGATAA	54.4	152	Genic	Ca1	203312	Zinc finger C-x8-C-x5-C-x3-H type family protein
CaSSR3	(TCT)7	ACGGTCAGTGAGCTGCTTCT	TCCCAATCCTAGCTACCACG	60.1	151	Genic	Ca1	317669	Pseudouridine synthase family protein
CaSSR4	(TCTCAT)4	GGTGCTACTGATTTCAATGTT	AGGAGCATCTTCTTCACTTTC	54.4	156	Genic	Ca1	389097	TCP family transcription factor
CaSSR5	(ATTCTC)5	CAACAACAACAACAAGAATCA	GTTTGAAGAAGGTTGTTGGAT	54.7	153	Genic	Ca1	390435	TCP family transcription factor
CaSSR6	(GA)11	TCAAAGAACCAAAATTCGAACA	CGGTTGATTGATAATTCTCGTG	59.6	153	Genomic	Ca1	472339	
CaSSR7	(CAT)5	AACCTCTTCTTTCACACCTTGC	GGGGATATGATCCAGCCTTT	60.1	279	Genic	Ca1	505905	KT2, a photosynthate- and light-dependent inward rectifying potassium channel
CaSSR8	(ATAAA)9	TGCTGCCAACAAACTTAGCA	GGCACCTTTCCTTGTACTGG	60.6	202	Genomic	Ca1	1145423	
CaSSR9	(AT)8	GGCTGTGTTTGGTTGTGTTG	TTGCATGCTTTTACCAAACC	58.7	180	Genic	Ca1	2046119	Putative secretory carrier membrane protein (SC3).
CaSSR10	(AAC)6	TGGGTTTGAGTTCTAAACAAG	CATTTCACAGCTTCTGATTGT	54.6	151	Genic	Ca1	2346126	Dof zinc finger protein adof2.
CaSSR11	(GAG)6	GGAAGCAGAAACAGTAGAGGT	TGTTCTTCCTCCTCTTTAACC	55.3	165	Genic	Ca1	2631745	zinc finger domain containing protein
CaSSR12	(TCT)6	GAAGCATCGTCTTCCCTGAG	AATCGTTGGCGTTGTTTCTC	60.1	192	Genic	Ca1	2994601	RNA helicase family protein
CaSSR13	(GGAAGT)3	ATAATGGTTACTTTCGGAGGA	TCTTCTTCGTGTTCATGTTCT	55.4	151	Genic	Ca1	3235738	sequence-specific DNA binding transcription factors
CaSSR14	(AT)9	TTGAACCCCAAATTCTCAGC	AAAGATGGTCCATAGTGAAGCAC	60.1	237	Genomic	Ca1	3688143	
CaSSR15	(GA)18	GTTAGGGGCCCATTTTGAAC	GATGGCTTCACGACCTTGTT	60.5	210	Genomic	Ca1	4097905	
CaSSR16	(CTGATT)4	CGCAGATTATATCTCACGGTA	CTATACCGTTTCAAATCCAAA	55.6	148	Genic	Ca1	4865629	BSD domain-containing protein
CaSSR17	(TTG)4	AAAAACAAGGAACCAAAGAAG	ATCTCCTCTATCATTGGTGGT	55.3	126	Genic	Ca1	5666044	nuclear coiled-coil protein related to the carrot peripheral nuclear protein NMCP1
CaSSR18	(TCT)6	TACCCAGATGAGATCTACGAA	GGATGGTATCACTGTTTGTGT	54.9	152	Genic	Ca1	6750597	myb-like HTH transcriptional regulator family protein
CaSSR19	(AT)14	CACATTTGTCATTTTGtAACAGCA	TGAGTTGGGAGGGGAGACTA	60.0	259	Genomic	Ca1	8867063	
CaSSR20	(ATG)5	AATTCAGGTCCTACAAAATCC	GAGCAGTGTGCCTATGATATT	54.8	151	Genic	Ca1	9791574	Glabra 2, a homeodomain protein
CaSSR21	(TCATGA)4	ATCATCATGAAGCATCCATAG	TATCGGATATAGCTTTTGCAC	55.1	156	Genic	Ca1	10682877	Involved in radial organization of the root and shoot axial organs
CaSSR22	(TCATGA)4	ATCATCATGAAGCATCCATAG	TATCGGATATAGCTTTTGCAC	55.1	156	Genic	Ca1	10682877	Involved in radial organization of the root and shoot axial organs
CaSSR23	(AATAA)4	CCTTTTTGGGTTCTTATGATT	ATTACACGAAGGATGAATTTG	55.0	151	Genic	Ca1	10893797	putative transcription factor
CaSSR24	(CAA)6	CCTTCACCTATCCCACTGGA	TGGGCCAAAGTGGATAAGAC	59.9	249	Genic	Ca1	11011602	Protein kinase superfamily protein
CaSSR25	(AT)11	AATCATAACCGATAACAAAACTCAT	TCATTAGACTCCAACAATTCATAGAC	57.3	268	Genomic	Ca1	11261320	
CaSSR26	(TAT)8	AGAAGAATCCAAATCCAAAAC	GAAGCCTCTTTCTCCTGATAC	55.0	148	Genic	Ca1	11285919	GRAS family Protein, transcription factor
CaSSR27	(CAA)6	CCACCTGTTACTATTCCTTCA	AAGGAGAATCAGAACCTTCAC	55	151	Genic	Ca1	11897074	DUF1635
CaSSR28	(GAT)6	ATGAGTCAAAGCCATAGTCAC	TTCTTCTTCAGTTGGATGAAA	55	153	Genic	Ca1	11899747	NAC
CaSSR29	(CTC)4	CTAAAGAATGGAATTGGGATT	CTCGTTTGTTTGCTCTATTGT	55	150	Genic	Ca1	11902768	SBP
CaSSR30	(AT)12	GTTGGTGCCAACATGCCTAT	TGAACCGTGtTTCaAAACCT	60.8	163	Genomic	Ca1	11960068	
CaSSR31	(CTT)9	TAAGTTGTGGCTGTGAGAGAT	GCATGAAAATTCAGAGAGAAA	55.0	157	Genic	Ca1	12928971	GLK1, Golden2-like 1
CaSSR32	(GAA)9	TGAGGATTCAAATGTTAAGGGG	TTCAGCAAGCAATGCAAATC	60.0	255	Genic	Ca1	14140590	Homeodomain-related
CaSSR33	(AAC)5	TCCTTTTGGTGACACTACAAATACA	AAAAAGAGACGCCATTCACG	60.2	277	Genic	Ca1	15231892	Eukaryotic aspartyl protease family protein
CaSSR34	(AAT)7	ACCTTATCAATGATTGCTTCA	CAAGACAAAGAGGAAATTGTG	54.9	154	Genic	Ca1	15378933	putative transcription factor
CaSSR35	(AG)10	GTTCCGAATTTGCGATCACT	TTTGCCGTTCATGGTGATAA	60.1	200	Genomic	Ca1	16531210	
CaSSR36	(AG)13	GAACTGAGCGAGGAAGTTGG	ACATCTCCGAACTCGACCAC	60.1	165	Genic	Ca1	16618090	putative TRAPPII tethering factor

CaSSR37	(TAA)7	TGAGATAATGGAGCTTGAATC	AGCTTGTTGAAGTGACACAGT	54.5	152	Genic	Ca1	20106435	basic helix-loop-helix (bHLH) DNA-binding superfamily protein
CaSSR38	(AAG)5	TTTCCTCACTTCAAAGACCCA	ATTTGACGAAACTCCAACGG	60.0	252	Genic	Ca1	21017902	IQ-domain 22 (IQD22)
CaSSR39	(ATC)5	AAGACCAATCATCAAACCGAA	AAGCTTGTGCAGGGAACACT	59.9	172	Genic	Ca1	21506732	YABBY family of transcriptional regulators
CaSSR40	(CT)12	GCGATCTCTCGAAAACCCTA	GAACGCAAACCACATGATTG	60.0	159	Genic	Ca1	21752019	ENSANGP00000022085 related
CaSSR41	(TA)12	TGAGAGAGGAAAAaCaGATTGAGA	CACGGATCTTGTGAGTGACC	59.5	141	Genomic	Ca1	30671033	
CaSSR42	(TCT)10	GCCATCTTCTACTTCCGCAC	AATTCGAACTCACCACTCGG	60.1	231	Genic	Ca1	31527936	putative protease SppA (SppA).
CaSSR43	(TC)7	TGAGAACAAGACCAGAGTCAC	GCATTTGTTCTTGAATCTCTC	55.3	144	Genic	Ca1	37689960	Possible function in phloem development in the root
CaSSR44	(CT)8	CCTCTTCAAACCATCCTCACA	TCTCGAGCGATCCATCTTTT	59.9	201	Genic	Ca1	43729171	Trypsin family protein
CaSSR45	(GGA)7	AGAGAAGAGAGAGGGGTCGG	CGACTTCGAGTTCCGTCTTC	60.0	184	Genic	Ca1	43894448	TT_ORF1, TT viral orf 1
CaSSR46	(AC)6cct(C)15	AAACGGATCAATGCGAGAAG	CATTACATTGTCATTTGGCCC	60.2	279	Genomic	Ca1	46935324	
CaSSR47	(AT)8	CTACCTTATGGGCACGCAAT	AGCCAATTTGGTGTTgGAAG	60.0	265	Genomic	Ca1	47131694	
CaSSR48	(ATTTT)4	AGGAATTAAGGTGATGAAAGC	GTTTAACAAGACGGACCAAAT	55.2	164	Genic	Ca1	47290885	BES1/BZR1 homolog 4 (BEH4)
CaSSR49	(TAA)7	CATGGAATTCCGGTGCTACT	GACGGTGTAGCTGCATGAAA	59.9	112	Genic	Ca1	48129325	TCP family transcription factor
CaSSR50	(GA)13	ATGGAGGCGAATATATAGGAG	TTTTAAGAAACAACTGCGTTC	55.1	151	Genic	Ca2	354109	LOB domain-containing protein 41 (LBD41)
CaSSR51	(GAT)6	GGAATTTTGGTGATGATTTTC	CAACATATGAAGGAACACACA	56.0	149	Genic	Ca2	1133300	WRKY Transcription Factor
CaSSR52	(TTC)11	GTCCCCCGCAGTTACTGTTA	GTAATTGTGAAGCCGGTCGT	60.0	218	Genic	Ca2	1588490	Protein of unknown function (DUF3531)
CaSSR53	(AC)6(A)10	TTGTTTCTCCAAACCAACTACA	TGCTTCCATAAGTTCTCCCG	57.3	119	Genomic	Ca2	1754600	
CaSSR54	(AG)11	TGAGAAAAATTAGCACACAAAG	ACATATTCATGCTGTTGTTCC	55.0	143	Genic	Ca2	2409848	homeodomain leucine zipper class I (HD-Zip I) transcriptional activator
CaSSR55	(ATG)5	TTGAAGGTGGTGATGGTGAA	GGGCACCTTATTAGCCCATT	60.2	169	Genic	Ca2	3226335	Glutaredoxin family protein
CaSSR56	(TTAA)3	CATCTATAAATGTGGCACAAG	GATGGAGTGAAGTTTGAGATG	53.4	128	Genic	Ca2	3236381	PRE1 (PACLOBUTRAZOL RESISTANCE1)
CaSSR57	(CACAAA)4	GGAGGTTATAACAACACCAAC	GGCTTTGTTATGTTTGAGGTT	53.7	150	Genic	Ca2	3451762	Mitochondrial substrate carrier family protein
CaSSR58	(TGT)5	CAGGAACCAAGATTGCAAGA	GGGAAAGAGTTCAAAACCCA	59.0	192	Genic	Ca2	3632977	SPX domain gene 4 (SPX4)
CaSSR59	(TCA)7	CAACCAAAAAGACAAGAATGA	GGCTCTTGATACCCTTACAAT	55.5	160	Genic	Ca2	4036035	a chloroplast trans-acting factor of the psbD light-responsive promoter
CaSSR60	(TCA)7	CAACCAAAAAGACAAGAATGA	GGCTCTTGATACCCTTACAAT	55.5	160	Genic	Ca2	4036035	a chloroplast trans-acting factor of the psbD light-responsive promoter
CaSSR61	(TGA)7	GGCTCTTGATACCCTTACAAT	CAACCAAAAAGACAAGAATGA	55.0	160	Genic	Ca2	4036194	a chloroplast trans-acting factor of the psbD light-responsive promoter
CaSSR62	(TAA)15	TAAAATCCTCCTCCACACCG	GGATGGACACATACATgGGG	59.9	169	Genomic	Ca2	4037173	
CaSSR63	(GA)14	GCAAAAGACACATTTAGGGTTT	GTAGGTGCAGGCCCTACCTT	57.4	270	Genomic	Ca2	4570670	
CaSSR64	(CCA)11	ACATTGTTGGTCGTGTTTGC	GGGTGATGTTGGAAGGATGA	60.7	259	Genic	Ca2	6404844	Core-2/I-branching beta-1,6-N-acetylglucosaminyltransferase family protein
CaSSR65	(TA)11	GAACCCCTTTcAACAAACGA	CGAGCCTCGTAAAATCCAAA	59.9	158	Genomic	Ca2	6749922	
CaSSR66	(AT)11	CCCTTCACTTTCTCACATAAAGG	AAGCCCTGTAACTCCAGTCG	59.2	195	Genomic	Ca2	8249226	
CaSSR67	(CTT)8	TCCTCCAACAACAACACCAA	AGGAGGAACCTTTGAAACCC	59.4	218	Genic	Ca2	9985718	Nucleic acid-binding proteins superfamily
CaSSR68	(TTC)6	AATGCCTTCACGAATTTTGG	AGGTTACTTCCTGCGGGTTT	60.0	240	Genic	Ca2	10263909	Expressed protein
CaSSR69	(AG)12	TTCCAGATCTCCGGTAGGTG	ACTCTCCACTCTCCCAACCA	59.7	209	Genic	Ca2	10330416	A basic helix-loop-helix encoding gene (BIGPETAL, BPE)
CaSSR70	(ATC)4	AGCAAATTTCATGCTACAAAC	TCCTAGTTCAATTCCTGATGA	54.8	151	Genic	Ca2	11661488	homeodomain leucine zipper class I (HD-Zip I) protein.
CaSSR71	(TC)10	TGGATCCATTTGGTTtGGAT	TTCTTTCTCGTCGACTCCGT	60.0	226	Genomic	Ca2	12299937	
CaSSR72	(GCA)4	CTTTAATCATGCCAGCTCTAC	TCCCTTTCTCTTTCTCTTTTC	54.4	165	Genic	Ca2	14624000	AL4, Alfin-Like family protein containing PhD domain
CaSSR73	(TA)10	TGGATGAGCCcTTCTTGAAC	TGAAATTTAAATGGGTGCCA	60.2	182	Genomic	Ca2	14846317	
CaSSR74	(ATG)9	CAGGCCTTGTTTGTTGAGGT	TTCTCCTCGATTTCAATGGG	60.0	206	Genic	Ca2	15235771	ABI five binding protein (AFP1)
CaSSR75	(TG)7(TA)8	TCGGAAAATTGAGAAAGTCTTCA	GCAAATGCTCCCCTAATTGA	60.2	276	Genomic	Ca2	15920661	

CaSSR76	(AT)10	TGACCATTTGCATTAGTTCTCAA	GCCACACCACtTTCAtTTTTC	59.6	246	Genomic	Ca2	17520553	
CaSSR77	(CT)8ttc(T)10	TCCGTTTAATTTTGCTTGCT	CGTGCACTTCAAAACCATGA	57.6	248	Genomic	Ca2	18353310	
CaSSR78	(ACA)8	CTCAAACTCCTCCACGAAGC	TAAACACCCATCTTGTGCCA	60.0	270	Genomic	Ca2	19292063	
CaSSR79	(TGA)4	TGTCTCATCTGAAAAAGATTGA	TTGGTTTCTTCATCAACAACT	55.1	150	Genic	Ca2	23951109	auxin (indole-3-acetic acid) induced gene
CaSSR80	(TTA)7	AAGATTTACCGCATCACATATCTC	CCTCCTACCCCACATTACCA	58.6	203	Genomic	Ca2	25670979	
CaSSR81	(CAA)4	AAAACAACAACTCTTCAATGC	TCTCCAATTGTAACCATCATC	54.6	155	Genic	Ca2	26834137	plant WRKY transcription factor
CaSSR82	(TC)12	CATCTCTCTCACTCTCTTTCC	AGATATGGGTAGCAAACGAAC	53.2	200	Genic	Ca2	27694606	ERF (ethylene response factor) subfamily B-5 of ERF/AP2 transcription factor family
CaSSR83	(TGG)6	TCTAGGCCTGGAAAAAATGGTT	TGATTCCCATTTCCTCTTGC	60.0	179	Genic	Ca2	27874806	Expressed protein
CaSSR84	(TTC)5	TCTTTCGCTTTCACTCCCTC	GGTGGAAGCGGTTATTGAGA	60.1	227	Genic	Ca2	28230844	Concanavalin A-like lectin Putative protein kinase
CaSSR85	(AT)16	TTGTCACCATTATCTCCACTC	CCAATATCTATTCATCTTCACCT	55.1	144	Genic	Ca2	29098865	protein disulfide isomerase-like (PDIL) protein
CaSSR86	(TC)9	AGCGATGAGAGCGAGTAAGC	TCTCTCCCCAATTCACCATC	59.9	278	Genic	Ca2	29913078	protein affected traf&#64257
CaSSR87	(CAG)7	TTGGTTAAGGATGTTCCTTCT	CTACCATCTGCTCTTGCTCTA	55.5	154	Genic	Ca2	30868340	CZF1
CaSSR88	(TA)11	AAAAACGCAAACACCaCTCC	GTGaAATAATTAGCTCTCTGATACCAT	60.0	237	Genomic	Ca2	31798047	
CaSSR89	(CAT)5	TGGAATGAaGAGATCcTCGC	CAAGTGGCAGCAGAAGTTCA	60.3	165	Genomic	Ca2	32013920	
CaSSR90	(GAA)11	CAAGTGAATGAATTCTCAAGC	CTCTCTTTCCTCTCATCTTCC	55.1	152	Genic	Ca2	32867739	EIN3 (ethylene-insensitive3)
CaSSR91	(GGT)4	CATGAACAACATCATCAACAA	CTCCACCACTACTTGTACCTG	55.3	148	Genic	Ca2	33605178	SHI gene family protein
CaSSR92	(TG)11	GGGGGTAAAgATTTCTCCACA	TCCCATAATTCAGGGATCTTG	60.2	225	Genomic	Ca2	33722683	
CaSSR93	(CTT)5	ACCATTTGGCATTGTTCCTC	GCGAAACCTGGAGTTCTTGT	59.3	266	Genic	Ca2	33745627	CLAVATA1-related receptor kinase-like protein
CaSSR94	(GAT)10	GCAACAAAAGAAAAGGCAGC	TTCTTCCTCCTCCTCtTCCC	60.0	195	Genomic	Ca2	34684799	
CaSSR95	(GAA)5	TTGAGCTGGAAGAAGGTCGT	CACTCCTCACATCCAACCCT	60.0	118	Genic	Ca2	35079352	trigger factor type chaperone family protein
CaSSR96	(CCA)6	ATTGATGACTCCGATTTCGC	ACCAGGTTGTCTACCCCAAA	59.3	148	Genic	Ca2	35422305	RNA-binding (RRM/RBD/RNP motifs) family protein
CaSSR97	(CT)6ca(CT)6	AAAATGCACTTGTGTTTTTGG	GCAACTTGATGCGAATTGAC	57.7	279	Genomic	Ca2	35431751	
CaSSR98	(TC)12	GATGGTGCTGTGAAATGTGC	TCCGCATTGAGATAAGGTCC	60.1	214	Genomic	Ca2	35754585	
CaSSR99	(TTCATT)4	AATGAGCTACTGCTGTCTTCA	CCAGCAAAGTAAAGAACTCAA	55.4	157	Genic	Ca3	80260	NAC domain transcription factor
CaSSR100	(TTTGAG)4	ATTCCACTTGTTGCTGATAAA	CTAAAAATGGAGGAGGAAGAG	55.0	151	Genic	Ca3	80320	NAC domain transcription factor
CaSSR101	(TTC)4	ACGCTTCAAGTATTGAACAAC	AAGGAAACCACACTTCACTCT	54.7	150	Genic	Ca3	81357	NAC domain transcription factor
CaSSR102	(AT)10	TGAAGCTAGGCTTGGTGGAT	ATTTTTCACCGACCGTTTTG	59.8	148	Genomic	Ca3	925403	
CaSSR103	(AAT)6	GGAGTTGGATTCTCTGGTGC	AATGGAACAAGTGGCTTTGC	59.7	240	Genomic	Ca3	2898679	
CaSSR104	(TC)14	CGGCCATTGAAAACGAATAC	TGGGTATTGGACTCCTTTCCT	60.3	266	Genomic	Ca3	3090422	
CaSSR105	(TAT)21	TGCTCTCTATTCGTTCCCTTTC	TCCGTTTAAGTTTCACTGCATTT	59.9	278	Genomic	Ca3	3745505	
CaSSR106	(TTA)31	TGTCATGCTTATTCCGACATCT	TCTcCCACCTCTGTTGCTTT	59.6	244	Genomic	Ca3	4772252	
CaSSR107	(TGA)5	TTGGAAGAAACACTGAATGAT	CTTTTTCTTCGTACGGTCATA	54.8	150	Genic	Ca3	7435046	SMAD/FHA domain-containing protein
CaSSR108	(AACAG)5	AGAGAAGAAGTCCAACAATCC	AGGTACAAAGAAAGTGCAACA	55.0	159	Genic	Ca3	10489944	ACT-domain containing protein
CaSSR109	(TTG)5	GGGTCATTTTGTTGCCTCAT	AATTTTGTGCATGACCCTGA	59.0	143	Genic	Ca3	12394016	Expressed protein
CaSSR110	(GA)9	TGAGGGAGAGAGACACATGAAa	CGGAAATTTTGTTGTCCAAG	59.8	212	Genomic	Ca3	15320676	
CaSSR111	(TTA)7	GAGGTTGGGGTTGGTTTCTT	CCATTCGCCTTCAATTTTGT	60.2	208	Genomic	Ca3	16451987	
CaSSR112	(ATTCAT)6	CCTTTCATTCATCACTTCAAC	AGGATAAAGGTGAAGCAAATC	54.8	165	Genic	Ca3	16813885	basic helix-loop-helix (bHLH) DNA-binding superfamily protein
CaSSR113	(TTTA)8	TGTGACATGATTTATGAATGACTTTTT	TTAGGGTTGTTCCAGTTGGC	60.1	207	Genomic	Ca3	17876577	
CaSSR114	(CATCAC)3	ACCATCATAACCATCATCATC	GGAAACCCTAGGTATTGTTGT	54.6	143	Genic	Ca3	20843695	one of two Arabidopsis RAPTOR/KOG1 homologs
CaSSR115	(CAT)6	CGTTTCCATTAGAGGAGAGAC	ATGAAAGTGATGGTGATAACG	55.6	162	Genic	Ca3	20843747	one of two Arabidopsis RAPTOR/KOG1 homologs
CaSSR116	(TCC)5	TCATCTGGGAAAAGGGAGTG	AAGGACCTCATCAAAACCAGAA	60.0	195	Genic	Ca3	21366194	WRKY Transcription Factor
CaSSR117	(AAC)8	ACACACTTCAAGAAACTTCCA	CTAAATCAGCAGCAGTAGGTG	54.9	150	Genic	Ca3	21774818	novel family similar to DNA binding proteins containing basic-leucine zipper regions
CaSSR118	(CTA)8	TCCCGTGAAACTGTCACAAA	CACCTGGAGAAGCTGAAATTG	59.9	277	Genic	Ca3	25631919	Expressed protein

CaSSR119	(CAA)7	TCTTACACCAAAACACAAACA	GAAGTGTTGTTGTTTCCCATA	54.3	154	Genic	Ca3	25672196	MYC-like bHLH transcriptional activator
CaSSR120	(AATGAA)4	AATAGTTCCAAAGGGTGAGAA	CCTTCTTGTTGGAATTGAACT	55.5	142	Genic	Ca3	26594189	GATA transcription factor gene GNC
CaSSR121	(ACA)7	TTTCAGTGTTTGTGGAAGACT	TGACTCATTTTGGTTTTGTCT	54.9	146	Genic	Ca3	26594514	GATA transcription factor gene GNC
CaSSR122	(TGT)4	TGAGTATCCCTTAATCACGAA	TGCACAGTAACAACAACTCAG	54.9	146	Genic	Ca3	27041765	BTB-POZ and MATH domain 4 (BPM4)
CaSSR123	(AT)7	CCAACCAATGAAAGCTAGGC	TCCTATACCAATCCCCCACA	60.0	134	Genic	Ca3	27694641	PHLOEM INTERCALATED WITH XYLEM (PXY)
CaSSR124	(CATCAC)3	AGATTACTCATTACCGCATCA	TCAAGCTATTGATCTTCTTGC	54.9	149	Genic	Ca3	28874001	HLH, Helix-loop-helix DNA-binding domain
CaSSR125#	(TAA)5	CGCATCTTCAATTCCATCCT	GGGCCCAACTCAAAAGTTTC	60.8	159	Genic	Ca3	29594640	ABC2_membrane, ABC-2 type transporter
CaSSR126	(CT)11	TGTCCCCATCTTCTTCATTCA	TTATTCAATGGGGTTCCAGC	59.8	241	Genic	Ca3	29681010	NIMA-related serine/threonine Putative protein kinases (Neks)
CaSSR127	(AT)16	TTCACAAACAACACCACACAa	TCACACGTGTCACAGTTCCA	58.5	174	Genomic	Ca3	30299506	
CaSSR128	(AG)6	TGAACCCAACAAGGAACCAT	CCCCATGTGCTAAAAGCAAC	60.5	223	Genic	Ca3	30690824	Plant protein of unknown function (DUF868)
CaSSR129	(TAT)6	AGTGGTGGAAGTATCCGTGG	AAGGATGAAAAACAGAGGGTG	58.2	237	Genic	Ca3	30756503	seed storage 2S albumin superfamily protein
CaSSR130	(GA)10	CCTCTGAAATGGGACTGTT	AACACTTCCCCACACAAAC	55.0	141	Genic	Ca3	31064037	ERF (ethylene response factor)
CaSSR131	(AAC)4	TTATCATCCAATGATGAATCC	ACTCCTCTAAACACCCTTTTG	54.8	155	Genic	Ca3	31248988	ERF (ethylene response factor)
CaSSR132	(TA)9	TCGTTTGCACTTGTTTTAGCC	TGTGAGCACTCATCGGTCAT	60.3	201	Genomic	Ca3	32341145	
CaSSR133	(TA)10	TCCTCCATTGCCTTTTCATC	GCATATTTTGGGACTAAGCATGT	60.0	229	Genomic	Ca3	32408558	
CaSSR135	(CTT)7	GCATCACAAGCTTCAACAGC	TTTTGGGTTGATGGGTGATT	60.0	213	Genic	Ca3	32584632	Chloroplast-targeted copper chaperone protein
CaSSR134	(CTT)9	GCATCACAAGCTTCAACAGC	TTTTGGGTTGATGGGTGATT	59.6	213	Genomic	Ca3	32584632	
CaSSR136	(AT)6(AG)7	AGGCTATAAGAATGTCcaCCTTT	AGAAATGCAAGCATCGTCAA	57.5	237	Genomic	Ca3	34902234	
CaSSR137	(CTT)5	ACATTCCCATTCCCATTGAA	ATTTGGAATCCATGGTGCTC	59.8	215	Genic	Ca3	35872876	FASCICLIN-like arabinogalactan protein 16 precursor (FLA16)
CaSSR138	(AG)19	ACCATTAACAGTCAACCATTG	CAGATATGTTCCTGCATTGAT	55.0	153	Genic	Ca3	36810456	group-S bZIP transcription factor
CaSSR139	(AAG)7	GATATCTGGATCGGGTCTATT	CATACCACACCATTTCAATCT	54.7	144	Genic	Ca3	36944041	Zinc knuckle (CCHC-type) family protein
CaSSR140	(TCT)5	TCCTAAATCTAATTCTATTTCGCATAA	ATCGCAAATGAAACGGAGTC	60.1	248	Genic	Ca3	37063772	RING/U-box superfamily protein
CaSSR141	(AATTGT)4	GGAACAACTCAAGCTCAGTAA	ATCCTTAGATCAGAGGAACCA	54.8	164	Genic	Ca3	37101948	squamosa promoter-binding protein-like 12 (SPL12)
CaSSR142	(AGA)6	CAGATTCCAAACGTGCAGTG	ATTGCAATGTGAACCCACAA	59.8	253	Genic	Ca3	37377922	Pectinacetylesterase family protein
CaSSR143	(GAC)4	TACATGTTCAGCACAGCTAGA	TCTTCTTCTTCGTCCTCTTCT	54.7	150	Genic	Ca3	37789311	ACT-domain containing protein
CaSSR144	(TA)9	AGGGAAACTTGACCCTCCAT	TGTTTGAAATTGCCACAAGC	59.8	191	Genomic	Ca3	38027665	
CaSSR145	(GAT)6	GAGCATCATAAGCGACGTGA	CCGCCAATCATATCCATTCT	59.7	140	Genic	Ca3	38354781	protein similar to glutathione synthetases
CaSSR146	(TAA)12	AATACGCATCCAATCCATCC	GTGTGGTGCTGCACAGAGTT	59.9	267	Genic	Ca3	38610591	Rubredoxin-like superfamily protein
CaSSR147	(GAA)7	CAAAGTAAACCCCACCGCTA	ACCGCAATATGGAACACGAT	60.2	259	Genic	Ca3	38624905	Expressed protein
CaSSR148	(TCA)8	AACACTTGAACATTTCAAACC	ATGGTGATGTTGTTACCAAAG	55.0	153	Genic	Ca3	38689024	Duplicated homeodomain-like superfamily protein
CaSSR149	(ATT)5	TCTCTCCCACAAAGGTCCC	GAAGGTGGCCAAGAGATGAA	60.2	134	Genic	Ca3	38689689	Duplicated homeodomain-like superfamily protein
CaSSR150	(ATC)7	ACCGTTCTCTAGGACGACCA	TGAGTGAAGATGATGACGGC	59.8	216	Genic	Ca3	39398787	WTF1
CaSSR151	(AG)7	GGTTTTGAGCGTGGTTTGAT	TGCAAAATGTGAAGCACAGA	59.0	146	Genic	Ca3	39618754	Expressed protein
CaSSR152	(AG)9(GT)6	TAGGTTTGTTGTGTGGGTCG	CCCTcACTCAGCGGTCTATC	59.5	142	Genomic	Ca3	39804933	
CaSSR153	(GAA)5	AGGAGGAGGTAGATATGATGG	CAAGTCTGTTCAGTTTCCCTA	54.8	156	Genic	Ca3	39817055	NGATHA3 (NGA3)
CaSSR154	(TATCAA)4	CCAAGGTCTAACTCACACTCA	TAGAAAGGAACTGTGATGAGC	55.4	134	Genic	Ca3	39817471	NGATHA3 (NGA3)
CaSSR155	(AAGA)6	ACTTCAGTGCTGTTTGATTTG	CCAACTAACAAACTTGGAAAC	55.6	155	Genic	Ca3	39818017	NGATHA3 (NGA3)
CaSSR156	(AT)14	TGGAGAGGAATTGTTAcGTGG	GCTTCCAATCAATAAAGTCTTACAA	60.0	222	Genomic	Ca3	39849058	
CaSSR157	(TCA)6	CGCAACTTCTCCCTTTCATC	TCATGGATTTCCTTTGCCTC	60.0	248	Genic	Ca3	39872382	endonuclease
CaSSR158	(TTTA)7	TTAGGCGCCACTCACTTTTT	TGTTCATTTGCATCGGAAAC	59.9	243	Genomic	Ca3	39872629	
CaSSR159	(CTT)17	CCGACCCAAcATTTTCATTT	TGCTGGAACGAACATTCAAC	59.7	174	Genomic	Ca4	820341	

CaSSR160	(TTGG)3	TTATGAAAATGATGGGTTGTC	AGACAACAGTGCCAAACTAAG	55.0	156	Genic	Ca4	1103152	NAC domain containing protein 74 (NAC074)
CaSSR161	(CAA)5	CGGTGATGAACCTGTTGTTG	AAGCCACTCAAGACGCTGTT	60.1	234	Genic	Ca4	1206702	12S seed storage protein
CaSSR162	(ATC)4	CAAGAAGTTTCTTCCACTTCA	CCAATCCATACTCTAACACCA	54.7	155	Genic	Ca4	1220485	ERF (ethylene response factor)
CaSSR163	(TA)8	TGTGCACATGACAATTAGAGCA	TTTCCATCAAGAACACGTCG	60.3	221	Genomic	Ca4	2543168	
CaSSR164	(TTA)5	AGATCCACCTCCACCTTGTG	TTGGAGGTTGTGTTGTTGGA	60.0	185	Genic	Ca4	2688512	AT-hook motif nuclear-localized protein 20 (AHL20)
CaSSR165	(AG)10	GTTAGGGATTTGTCGGAGCA	CAACGGTCACCTTTTTGGTT	60.1	217	Genomic	Ca4	3603795	
CaSSR166	(TA)8	CCACCTCTTCCCATCACTCA	TCATGGAGAATCCCAGATCC	59.8	271	Genic	Ca4	4594795	AT-hook motif nuclear-localized protein 19 (AHL19)
CaSSR167	(AG)6	GGGCAGTGAGAGATGAGAGG	CCTCACCCTTTTACCCACAA	59.8	148	Genic	Ca4	4699818	Nuclear protein that mediates light regulation of seedling development in a phytochrome-dependent manner
CaSSR168	(GATTCA)6	TGCAACTAGAGAAAGCTATGG	TGCGTTTTCTTTTAGTGCTTA	55.1	156	Genic	Ca4	4799223	ATRX
CaSSR169	(GATTCA)6	TGCAACTAGAGAAAGCTATGG	TGCGTTTTCTTTTAGTGCTTA	55.1	156	Genic	Ca4	4799223	ATRX
CaSSR170	(AAT)4	GAGAAAGAGGATATTGGGAGA	CTAACTCCAACATTCCTTGTG	55.0	151	Genic	Ca4	5080649	Duplicated homeodomain-like superfamily protein
CaSSR171	(ATA)10	CGGTTTGTTACTGAACTTGAA	CTTTGTCACCAATAAATCGTC	55.6	150	Genic	Ca4	5081312	Duplicated homeodomain-like superfamily protein
CaSSR172	(AAT)7	GTAGTGCAGATAAATGCAAGG	CAAGTTCAGTAACAAACCGATA	55.2	141	Genic	Ca4	5081434	Duplicated homeodomain-like superfamily protein
CaSSR173	(CAA)4	GACTATTCAATCCACTGGTCA	TTGAAATCAGTTACCTTCTCG	55.1	159	Genic	Ca4	5571877	UDP-Glycosyltransferase superfamily protein
CaSSR174	(ATC)4	AGATGATGATCACAAACATCC	TTGAAGTGAAGGAGATGAAGA	54.8	142	Genic	Ca4	5736891	indeterminate(ID)-domain 7 (IDD7)
CaSSR175	(AAC)4	TCATGACCTCATATCAAAACC	AGTTGCAGCAGATAAATGAAG	55.0	160	Genic	Ca4	5736936	indeterminate(ID)-domain 7 (IDD7)
CaSSR176	(ATG)8	TCAAAGGGAAAGGATTTTGG	AGGGTCCCTCAGTATTGCCT	60.0	145	Genic	Ca4	6590103	Major facilitator superfamily protein
CaSSR177	(CAA)7	CGGTGTGAATTTGGTGATGA	CAACAACAACCGAACGAGTG	60.2	253	Genic	Ca4	7315077	Tetratricopeptide repeat (TPR)-like superfamily protein
CaSSR178	(GAGAAA)4	TGAAACTTCAGAGGTTGGTAA	GCTTCTTGCTCTTGTTGTTC	55.0	144	Genic	Ca4	7561185	NAD(P)-binding Rossmann-fold superfamily protein
CaSSR179	(TGG)8	ATCCGGTAAATCAACATGAAT	CAGAACCCAGTTCTCATGTAA	55.9	157	Genic	Ca4	7970786	Zinc finger C-x8-C-x5-C-x3-H type family protein
CaSSR180	(ACCCT)5	TTCCTAAACGACATCTTCAAC	CAGTTTCTTCCACAAAAGCTA	54.5	153	Genic	Ca4	8051984	TLP family
CaSSR181	(ATG)7	GAAACTGAGCACTAGGAGTTG	CTTCTCTCTGCTTCATCTTCA	54.3	169	Genic	Ca4	8052130	TLP family
CaSSR182	(ATG)6	TAAACAAACCGGGAAGTTGG	CCCCTTACGAATCCCATTTT	60.0	137	Genic	Ca4	8052218	TLP family
CaSSR183	(GA)9	GAATCAGAAGGTGGTTGGGA	TTCGTGCCAGAAACAGTGAG	60.0	155	Genic	Ca4	9314839	protein with a DWD motif
CaSSR184	(TA)8	TGCATCTCGCCTTGAATATG	CCACACATGCCCCTACTCTT	60.0	214	Genic	Ca4	11051159	NAC domain containing protein 16 (NAC016)
CaSSR185	(GAT)4	AGGTTTTCAAGAAAAGTGGAC	GAGGATCAATAACAGCATTTG	55.1	147	Genic	Ca4	11053035	NAC domain containing protein 16 (NAC016)
CaSSR186	(ACC)5	ACTATTATGCACCCACTGAAG	GCAGAGACGAAGAAGATGATA	54.5	138	Genic	Ca4	11573408	Duplicated homeodomain-like superfamily protein
CaSSR187	(GAA)7	TGCTGGTCTTGTAGCTGGTG	GCAGCTTGTAAAGGGTTTGG	59.7	168	Genic	Ca4	11658503	Vacuolar iron transporter (VIT) family protein
CaSSR188	(CTCATC)4	CCACTAATACAAGGAAGCAAA	AGTCGCTCTTATTGGACTTGT	54.8	156	Genic	Ca4	12023919	Leucine-rich repeat protein kinase family protein
CaSSR189	(CTCATC)4	CATTCTTCAAATTCAAACCAC	TGGAAAAAGAAAGACAATGAC	54.8	136	Genic	Ca4	12023936	Leucine-rich repeat protein kinase family protein
CaSSR190	(AAC)7	CCCAAAACATCAACAATCTTA	CCTGTTGGAAATTTCTAGGTT	55.3	132	Genic	Ca4	12176090	SCARECROW-like 8 (SCL8)
CaSSR191	(TCA)4	CTAACTGAACCACCAGCACTA	AGAAGGAGAGGTGATAGGTTG	55.6	146	Genic	Ca4	13843207	LUH | WD-40 repeat family protein
CaSSR192	(AG)9	AAAATTGGATTGGGAAAGGG	TTTGCTACCAAACCAAACCC	59.8	131	Genic	Ca4	14340497	tubulin alpha-6 chain
CaSSR193	(AT)13	GGGATGCATCTGCAAATTTTA	TGAGTCCAATAAAACCCCCA	59.9	249	Genomic	Ca4	15398881	
CaSSR194	(AT)8	AACACCCCACATAATGACCC	TCATTTGACATTCACCTCACTTTT	59.9	222	Genic	Ca4	16478585	Expressed protein
CaSSR195	(ACTTGC)4	TGAACTCGTATCTTCTGCTTC	ATCATGCTTCACAACTTCATC	54.8	172	Genic	Ca4	16650296	ovate family protein 13 (OFP13)
CaSSR196	(ACTTGC)4	TGAACTCGTATCTTCTGCTTC	ATCATGCTTCACAACTTCATC	54.8	172	Genic	Ca4	16650296	ovate family protein 13 (OFP13)
CaSSR197	(TC)13	CGTCGTGCAGATCTTCGATA	GCGACGTCACTGTAAACCCT	60.0	248	Genomic	Ca4	16665218	
CaSSR198	(AT)14	CAATTTCCTCCTATTTGTTTTGACA	TGATTCTCGAGGATGCGAC	60.6	250	Genomic	Ca4	17043109	

CaSSR199	(CCA)6	CCAACTAGGCAAAAGCTTCG	GTTTGCAGGTTTGAGCTTCC	59.9	273	Genic	Ca4	17663778	RING-H2 protein that interacts with the RING finger domain of COP1
CaSSR200	(AT)9	AACTGAGAATGACTTGCCTTCA	TTTAGGGATGATTGCTTGGG	59.0	228	Genomic	Ca4	17704840	
CaSSR201	(ATT)8	TCTTTGAGACTGTTTTTCCCTTT	CATGTCTCATATTCCTCTAGCTGC	58.5	115	Genomic	Ca4	19856773	
CaSSR202	(ATTCT)4	GGGTCATCATCAAAACCA	AGTTCTGAAGTCATGGTAGGG	54.9	147	Genic	Ca4	20436604	TCP family transcription factor
CaSSR203	(TTG)9	CAGGAGTTGAAACTGAAACAC	TCCACAACAACAACAACAGTA	54.9	155	Genic	Ca4	22196748	embryo sac development arrest 31 (EDA31)
CaSSR204	(TA)10	AACCCATCATTTTGCCATGT	CCTGGAAAACTTGCAACTGG	60.1	155	Genomic	Ca4	22226606	
CaSSR205	(TA)10	GGGGATTGTCAGAAACGGTA	TTGTCGACACATCCTCTCAAA	59.8	223	Genomic	Ca4	26366347	
CaSSR206	(TCA)6	ACCACCACTGCCTCTTGTTC	CTCCAACAACTTCCCACGTT	60.0	149	Genic	Ca4	26873093	Expressed protein
CaSSR207	(AG)13	CACAATCATCATCGAGCACC	ACACACTCCTCACCGCTTCT	60.1	222	Genomic	Ca4	27187560	
CaSSR208	(AG)7	TCCACACAGACATGGTTCATC	CCTCATGGAAGGCAACCTTA	60.1	207	Genic	Ca4	28594218	AT-hook motif nuclear-localized protein 20 (AHL20)
CaSSR209	(CAG)6	TGGCATCATCATCACTGTCTT	CCATCCTTTTCAATGCGAGT	60.1	253	Genic	Ca4	30414975	cobalt ion binding
CaSSR210	(AT)9	TGTGAACTTTTCATTTGGCTTG	CCATTCGATCATGGGTAGTAGTT	60.1	205	Genomic	Ca4	30455676	
CaSSR211	(AAT)7	CCCCCACAAACTCAAAGTAAG	GCTGCATTTTCACCGTACAA	58.6	265	Genomic	Ca4	32335239	
CaSSR212	(ATGGT)3	TCCCATCAAGATGCTAAAATA	AATCAATCTTTGAGTTGTTGC	55.0	149	Genic	Ca4	32780320	SHY2/IAA3 regulates multiple auxin responses in roots
CaSSR213	(CAA)5	AAACAACTAACGCCGTAACTA	TTGGCATACGTATTCTTCTTC	54.5	153	Genic	Ca4	35416175	TCP family transcription factor
CaSSR214	(CCATT)4	TTCATTTAGCTTGAAGCACTC	GGTTGAGGAGAAAATTAAAGC	55.0	150	Genic	Ca4	35417258	TCP family transcription factor
CaSSR215	(TCC)5	AAACACAGATGTCGTCGCAA	CACTGATATTCGGCAACACG	60.1	156	Genic	Ca4	36155130	Plant protein of unknown function (DUF827)
CaSSR216	(CAC)6	CGTCGTTCACATACCAAACG	CCAGAAGGAGATCCTGAACG	59.8	207	Genic	Ca4	36842702	Putative plant-specific transcriptional regulator
CaSSR217	(CAC)8	CTAAAGAATGGAATTGGGATT	CTCGTTTGTTTGCTCTATTGT	55.0	153	Genic	Ca4	36842734	Putative plant-specific transcriptional regulator
CaSSR218	(GAT)4	TGAAGTTGAGAGGTTAATTGG	TCATCACACATAGAGCCTTTT	54.6	151	Genic	Ca4	36952282	indeterminate(ID)-domain 5 (IDD5)
CaSSR219	(TA)13	CTTCAACCCACCACGCTACT	CTCACTTCCTCCGTCTCTGC	60.2	271	Genomic	Ca4	38376225	
CaSSR220	(ATAA)7	TCTCCTTTCCCTCTAGTAATTTGTG	TTTGTTTTAAAATTGTCACTTCAACA	59.3	272	Genomic	Ca4	39006837	
CaSSR221	(TA)9	CATTCCACCTTAAAACTCCTCG	CCGTCTAACTAAGATGAACCCTG	60.0	199	Genomic	Ca4	39226042	
CaSSR222	(TA)6	ATAGCAGCAAATCCTCAGCG	GCTCATTGAAGATGACATTGC	58.3	273	Genic	Ca4	39805351	putative c-myb-like transcription factor
CaSSR223	(TC)9	TGTATGTAGGCTTGGGGAGG	ATCCAGATGATGCCCCTACA	59.9	170	Genomic	Ca4	40276977	
CaSSR224	(CT)7	CCTCAAGTGCAACAAAAACAA	TGCAAACATTTTCACACCAGA	60.1	124	Genic	Ca4	40670097	CCT motif family protein
CaSSR225	(CAG)5	TGCAACAGATGTTGTTACAGA	GAGATGAGCCCTATCCCTAC	55.3	170	Genic	Ca4	41865067	LEUNIG
CaSSR226	(CAG)4	AATTAAGGCTAGGGAACAACA	CATGCCTCTGTAACAACATCT	55.7	143	Genic	Ca4	41865183	LEUNIG
CaSSR227	(TA)12	ATGCCATTCTTGCCTGATTT	TCAAATGCAGCAACAACAAA	59.5	269	Genomic	Ca4	42025608	
CaSSR228	(ACA)4	GTTTGGATGCATAACAACAAG	CTCAGCATTCATATCAGCTTT	55.8	153	Genic	Ca4	43462315	homeobox protein 34 (HB34)
CaSSR229	(GAA)5	GAAAGAAGATCACAAGCCTTT	CAAAGGTATCTTGAGTGGTTG	55.3	155	Genic	Ca4	43513782	basic helix-loop helix transcription factor involved in tapetal cell development
CaSSR230	(GAA)8	TAGTTGCTGCCTTCGGAACT	GGCGGATACTACTTTGTCGC	59.7	200	Genic	Ca4	43715230	Ubiquitin-like superfamily protein
CaSSR231	(AATTAA)4	AATGAATTGGTTTGTTCTGTG	CTTACTCACCCGATCCTAACT	55.2	148	Genic	Ca4	43891008	A class II knotted1-like homeobox gene family
CaSSR232	(AGA)8	GTTTATGGTGTTGAAGGTTGA	TACTCTACGCTTCTTCCCTCT	55.2	149	Genic	Ca4	43891170	A class II knotted1-like homeobox gene family
CaSSR233	(AT)10	GTAAGCCAACTTCCTTGGCA	AACCCACAACCACTTCCATC	60.2	196	Genomic	Ca4	44581810	
CaSSR234	(ATG)5	AATGTTTCAATTTTCAATGGGA	GGATCGGTTATCGACTGAGG	59.5	189	Genic	Ca4	44625382	Expressed protein
CaSSR235	(AG)10	GGACACACAACAGAGAGAAAA	ACGAGGATTGTAAGGAGTACC	55.3	167	Genic	Ca4	44660343	AL4 the Alfin-Like family of nuclear-localized PhD domain
CaSSR236	(CAT)6	TCGCATAGAAGATGTCGTCG	ACGCTACTCGATGACGAGGT	59.9	152	Genic	Ca4	45074489	protein containing N-terminal tripartite nucleotide binding site
CaSSR237	(ATTT)9	GCTTCAGAATTCGACGTGGT	TACATTGGGCTGGTACATGG	60.3	105	Genomic	Ca4	45108920	
CaSSR238	(TGAA)3	ATGGAGGAAATTCTGAAACAT	AAGGAACTGTCTGTTGAAA	55.2	150	Genic	Ca4	45186457	transcription factor involved in photomorphogenesis
CaSSR239	(TAT)12	GGGTGTGGATAGCAATGGTT	AGCTCAATTGCCAGGAAGAA	60.0	274	Genic	Ca4	45445913	Putative homolog of the Blind gene in tomato

CaSSR240	(GTA)5	GGCGGAATAATTGAAGTAGTAG	GTTGTCGATCAAAAGTATTGC	55.0	158	Genic	Ca4	45595013	Growth regulating factor, transcription activatorr
CaSSR241	(GT)7	TTTTGTTAGCGTTTGTGTTTGG	CATAAAACCGCCTGCAACTT	60.1	203	Genic	Ca4	46259055	F-box protein
CaSSR242	(TC)10	CGTAACCGAGGAGTTTGGAA	AAGGGCGTTGAAAGAAAGAA	58.9	217	Genic	Ca4	46269177	Mannose-P-dolichol utilization defect 1 protein
CaSSR243	(AGATGA)3	CGATGAATTCAAGAAGCAATA	TGAACCTAACCAAATTCTTGA	55.7	159	Genic	Ca4	46398833	DREB subfamily A-5 of ERF/AP2 transcription factor family (RAP2.1)
CaSSR244	(AG)10	AATCAGAAGCGGAGTGGTTG	AATGGAGGCGGGAGAGTAAT	60.3	266	Genomic	Ca4	46457612	
CaSSR245	(TTA)21	ACAGAGCCACCAGGATTGTT	GAAACTGGCCAGACGtTTTT	59.6	225	Genomic	Ca4	46788418	
CaSSR246	(CT)7(AT)7	ATCTCGCCAATAACCACCAC	GGTAGCTAGTTGATCAAGTTCTTTCTT	59.8	197	Genomic	Ca4	47266711	
CaSSR247	(GAA)7	TGACATCTCCAACAACAAGAT	CACACTGTGGATTCTTGTCTC	55.6	147	Genic	Ca4	48124978	BEL1-like homeodomain 7 (BLH7)
CaSSR248	(ATA)6	GGCTATGCCAACATTCCTGT	TGAAGTAGGCCAATGCAACA	60.3	248	Genic	Ca4	48145725	cytosolic thioredoxin
CaSSR249	(TAAA)5	AAAGCATGCACTAATCACAGT	CAAGTCTTTGTCATCTTTGCT	55.1	139	Genic	Ca4	48319615	Basic-leucine zipper (bZIP) transcription factor family protein
CaSSR250	(TC)9(T)10	TTACATGAAAAATAAAAaCAAGCAA	CCGGAAGATTTAGCAATGGA	57.3	199	Genomic	Ca4	48825962	
CaSSR251	(GAT)6	ATGAGTCAAAGCCATAGTCAC	TTCTTCTTCAGTTGGATGAAA	54.4	153	Genic	Ca5	164592	LONG VEGETATIVE PHASE 1 (LOV1)
CaSSR252	(TCCC)3	CGAGTATACAAGAGAGCAGGA	AGCTGCCTTATTAGAGTGTCC	54.9	147	Genic	Ca5	166052	LONG VEGETATIVE PHASE 1 (LOV1)
CaSSR253	(CAA)5	TGACAACAGTTCTTGGTCTTT	GGGAGAGGGAGTAACAAAGTA	54.9	154	Genic	Ca5	166287	LONG VEGETATIVE PHASE 1 (LOV1)
CaSSR254#	(AATCCA)5	ATTCAGCAGTTAGTTCCAACA	GCATCAGCAAAGTAGACAAAT	55.1	147	Genic	Ca5	166458	LONG VEGETATIVE PHASE 1 (LOV1)
CaSSR255	(CTTTG)5	AATGGAGAAAAAGCAGAAGAC	GCGTAAACTAGATGGCTGATA	55.3	144	Genic	Ca5	222915	homeobox protein 31 (HB31)
CaSSR256	(TA)11	ATTGGGAATTCAGCGTTTCC	ACTGAGAATTGGGGTCgTTG	61.2	280	Genomic	Ca5	7247789	
CaSSR257	(ATTA)3	AACATGATGGAAGATGGAAC	TGGTCACTTGGGCTAATG	54.8	147	Genic	Ca5	7675104	a MADS box transcription factor expressed in the carpel and ovules
CaSSR258	(CT)15	TCTGCCTCGTACTCCTCACA	GGTTGTTGCTGGCATTTTCT	59.6	247	Genomic	Ca5	8866821	
CaSSR259	(TGT)4	TTGTTGATGTTGATGTTGCT	CCTATCAACCTATCGAACCTT	55.0	144	Genic	Ca5	9961600	WRKY transcription factor 2
CaSSR260	(TGC)5	ATTGATGATGAACCATTATGC	ACAACAACAACATCAACAACA	54.9	144	Genic	Ca5	9961692	WRKY transcription factor 2
CaSSR261	(ATA)6	TCGTCATAGTTTCAAGCGGT	CAGGAGGTGCACTATCAGCA	60.0	149	Genic	Ca5	9989246	chloride channel protein
CaSSR262	(TTC)9	TGCTATTGTGATTCAGTTCCT	AGCATGACAAGTTTAAAAGCA	55.0	147	Genic	Ca5	10080588	myb family transcription factor
CaSSR263	(AG)16	CAGAAAACAAACCAAAACCGA	TGCTCATGCATTTCAACCTC	60.0	191	Genomic	Ca5	10184550	
CaSSR264	(CTT)7	TCCAAAACACATTCACAAGCA	TATGGATCGGAAGACGAACC	60.1	245	Genomic	Ca5	11065299	
CaSSR265	(CTT)6	CCAAAACCATTTTCCTTCAATC	TATGGATCGGAAGACGAACC	59.9	191	Genic	Ca5	11065353	Plant invertase/pectin methylesterase inhibitor superfamily
CaSSR266	(AG)14	CGAATAAATTTGGCGGAGAA	AAGCATCCAAATTGGCAAAG	60.0	275	Genomic	Ca5	12476511	
CaSSR267	(GT)9(AT)6	AAGACATTATGAAATGCAACCAAA	TGAGAGATGCATTCAACATTATAGG	59.8	250	Genomic	Ca5	12592203	
CaSSR268	(AAT)10	TGATTTCTCCATCTtATCGGG	ATTAGCGCCTTACGACATCC	59.0	259	Genomic	Ca5	14185310	
CaSSR269	(ATT)8	AAAaGAACAAAATGGAAGCCC	TGGATGCAAAAGATTTGAGC	59.5	266	Genomic	Ca5	15878709	
CaSSR270	(AT)10	TCACGTCCATATTAATCATCAATTC	AGTGATAACCGTTGGAAAAGC	59.2	280	Genomic	Ca5	17120195	
CaSSR271	(CAC)4	CTTATGACATGAGGAAGCAAC	ATTTCTCTCACAATCAAATGG	55.0	150	Genic	Ca5	19048743	Acyl-CoA N-acyltransferases (NAT) superfamily protein
CaSSR272	(TCA)4	CAAGATTATCATTTGGACGAC	AAATGGTGCTAATTTGGATCT	54.7	150	Genic	Ca5	21386676	Pseudo response regulator involved in the generation of circadian rhythms, TOC1
CaSSR273#	(AT)10	CCTTTATAAAAaGAGGTGGGgT	GTGGGTTGCAAGGAAGATTG	57.7	201	Genomic	Ca5	23012241	
CaSSR274	(AT)12	TGCTTAAAGAATTTGAAACGATGA	CATTAGCCTCGTTGGCATTT	60.1	210	Genomic	Ca5	23438498	
CaSSR275	(AG)13	TACAAAAGAATCAAACCCAAG	TTTCTTCCACAATTCTCGTTA	54.6	165	Genic	Ca5	23874393	BTB/POZ domain-containing protein
CaSSR276	(AAT)7	TCCACTTCACCACATCATCA	TGCATATTTTGACCCTGCAT	59.0	209	Genic	Ca5	24717518	Homeodomain-like transcriptional regulator
CaSSR277	(AT)10	AACCGTTCACAGTAGAGGCA	CCCTATGTCCCAAAGCTACG	58.4	147	Genomic	Ca5	26329409	
CaSSR278	(GAT)7	TGAGGGTACTCCTAAACCCG	CCCCCTTCTTTTCCTTCAAC	59.0	180	Genomic	Ca5	26833175	
CaSSR279	(GAAA)3	AGCTATGCCTCCTGATAAACT	GAGTAGTGGCTTCTGTTTGAA	55.0	167	Genic	Ca5	27118508	basic leucine-zipper 52 (bZIP52)
CaSSR280	(CCT)4	AAGTAAACCACACGAGTTCCT	TATGAAGCTTTCAACATCAGG	55.5	144	Genic	Ca5	27757413	auxin response factor family protein
CaSSR281	(AAT)8	GCACATAGCAGTTACATATGGCAG	TACTTTTGCACCTCGGTTCC	60.9	265	Genomic	Ca5	28955959	

CaSSR282	(AT)7	CACAGCAACAGAACTGGCAT	TTCATGGCACAAGAAGTTGC	59.8	267	Genic	Ca5	29319100	protein, expressed in leaves, with similarity to pollen allergens.
CaSSR283	(CCTTCT)3	AGTTCTTCCACAAATTCAACC	CCAAGAACAGAACAAGAAGAA	55.8	152	Genic	Ca5	29398059	DREB subfamily A-6 of ERF/AP2 transcription factor family (RAP2.4)
CaSSR284	(CTT)5	TTCTCTTCCTCCATCAATGACTT	GCGAAAAGTTGGTTGGAAAA	60.1	155	Genic	Ca5	29465178	Contains a weak similarity to ELG protein from Homo sapiens
CaSSR285	(TCTTGC)3	TCTTTATCTTCTGCAAAACCA	CAAGAGCAAGAACAAGAAGAG	55.3	149	Genic	Ca5	30587088	nucleic acid binding
CaSSR286	(CTTCTC)4	TTTCATCGTCTACTTGATGGT	TTGATGGTAGAGCAAGAACAT	54.8	157	Genic	Ca5	30587245	nucleic acid binding
CaSSR287	(CTTCTC)4	TTTCATCGTCTACTTGATGGT	TTGATGGTAGAGCAAGAACAT	54.8	157	Genic	Ca5	30587245	nucleic acid binding
CaSSR288	(TTCTCC)3	TTTCATCGTCTACTTGATGGT	TTGATGGTAGAGCAAGAACAT	54.8	157	Genic	Ca5	30587245	nucleic acid binding
CaSSR289	(TAT)7	ACGCCTTCTTTGCTCCTTTT	AGTCAAGCCTCACTCCCAGA	60.4	250	Genomic	Ca5	31109261	
CaSSR290	(TTC)5	ATGAAATTGCTCCGTTGAGG	GGGATTTGATTCGTGGAAGA	59.9	258	Genic	Ca5	31182241	Calcium-binding EF hand family protein
CaSSR291	(ATG)6	CATGAAGGCCTATAACAAGAA	GAGCTTCTACTCGTCATCATC	54.7	145	Genic	Ca5	31194921	HMGB (high mobility group B) protein
CaSSR292	(ATT)9	AATTAGGGTTGGATGGAGGG	CTTCCGAACCACTACGCTTC	59.9	175	Genic	Ca5	31744505	RING/FYVE/PHD zinc finger superfamily protein
CaSSR293	(TC)6(TA)8	TCAATCTCCATCTTCCCCTG	CAACAAgGTCCCCACCAATA	60.0	222	Genomic	Ca5	31861702	
CaSSR294	(TG)7	TTTTTCCCTTTATCGCATGG	TAGGGGAAGGCAAATGTACG	60.0	145	Genic	Ca5	32856391	Expressed protein
CaSSR295	(CGG)5	CTGAGGATTTTGAAATTGAAC	TCATCTACACTCCGATCTCAC	54.0	145	Genic	Ca5	34050111	WRKY Transcription Factor
CaSSR296	(TAT)8	CCAGTTGTTGCCTCGGTATT	CAGGTTGATGTCCGAATGTG	60.0	273	Genic	Ca5	34521276	Expressed protein
CaSSR297	(AAG)10	CCAGCCATGGTAATTTGGAC	TTCTACAACCGTTTTTCTTCTCTG	60.2	204	Genomic	Ca5	34626059	
CaSSR298	(ATAA)9	TTtGGTTGCTAGACCAAGGG	CGGAATCCGATTGCTTCTAC	60.1	230	Genomic	Ca5	35432669	
CaSSR299	(AG)6	AAGCATCAGAAGAATCAGACAGG	CTTCCTCCTCGAGATCCTCC	60.3	220	Genic	Ca5	35796488	AGC (cAMP-dependent, cGMP-dependent and protein Putative protein kinase C)
CaSSR300	(CTTC)7	TCTAGAAGCTTCCTACTTCACC	CGAAAGTGAGTGTTGAGAAAG	54.7	163	Genic	Ca5	35861292	Heat Stress Transcription Factor (Hsf) family
CaSSR301	(TAT)5	CACTCCCTTCCTCCCAAAAT	CGCCTTTCGAATTATTGCAT	60.1	212	Genic	Ca5	36421460	Trypan_PARP, Procyclic acidic repetitive protein (PARP)
CaSSR302	(ATA)6	TCCATTTCCTTCCTTTTCCTC	GGTAGAAGGTGCTTGACCGA	60.3	219	Genic	Ca5	37739978	Chondroitin sulphate attachment domain
CaSSR303	(TTG)4	GGTATGACCAGAACATTTGAA	AACATCAATTCAATCATGGAG	55.1	142	Genic	Ca5	37896821	protein similar to a subunit of the CCAAT promoter motif binding complex
CaSSR304	(AT)10	AACAAGTCTGCAACAACCTTCT	CACAAAGTGAAGGGGGTCAT	58.0	270	Genomic	Ca5	38903229	
CaSSR305	(ACC)9	TTCTCTGAATCCAACGGTCC	TGTGTGCGTGAGAGAAGAGG	60.2	149	Genic	Ca5	40667653	Nodulin MtN21 /EamA-like transporter family protein
CaSSR306	(AAC)7	CTCTTGCAACTTCCCCACTC	AAAGCAAAGGAGGGTTTGGT	60.0	150	Genic	Ca5	41308534	Expressed protein
CaSSR307	(AG)6	ATGGTTTTGCTCACATTCTTA	TCTCAATTCTCTTCAATTCCA	55.0	151	Genic	Ca5	42499312	the MADs box transcription factor family
CaSSR308	(TC)11	CAGTGCCATCACTCCATCAC	CACGCaAAAACAAACCACAG	60.1	252	Genomic	Ca5	42534709	
CaSSR309	(GAA)4	GGAGGAAGAGGAAGAGAGAAT	GAGAGGACCTCAATTTCTTGT	55.7	172	Genic	Ca5	44153203	SWI3 gene family protein
CaSSR310	(ATG)4	TATAAACCACGCAAGTATTCG	GTACCCATCAAATCAACTTCA	55.6	146	Genic	Ca5	45306950	Homology Subgroup III
CaSSR311	(TA)6	CCGGCTTAATGAAAGAGGAA	GACCATGGGATGGATGTTTC	60.0	209	Genic	Ca5	45479771	Expressed protein
CaSSR312	(AG)8	AGAGTCAAATTAACAAAGAGCATAGA	AAAATGCCCACAAGAGCAAG	60.2	122	Genic	Ca5	46316346	CYP714A
CaSSR313	(CAC)9	AACCCATTTTGCAATCTGCT	CGCTGAGGAGAGAGTTCCAC	60.1	124	Genic	Ca5	46360697	RING/U-box superfamily protein
CaSSR314	(CCATA)4	AAAACAACACTCTCCTCTTCC	ACAGGTAGTGGGTATATTCCAG	55.1	143	Genic	Ca5	47007215	Transcription elongation factor (TFIIS) family protein
CaSSR315	(CAA)7	CAAGCTCACACTGAACTCTCT	GTTGTTGAGTTGTGGTGATTT	54.8	146	Genic	Ca6	466443	KANADI protein (KAN)
CaSSR316	(TCATTT)3	TCATAGATTCTTCTCCCTTCC	TGGAAACTTATAGATTTTGTGG	55.0	156	Genic	Ca6	1609131	DA1-related protein 2 (DAR2)
CaSSR317	(TTCTCT)3	CTCCACATCATTTTCATTTTC	AAACCAGACTTCCTTTCTGAG	54.7	173	Genic	Ca6	1609211	DA1-related protein 2 (DAR2)
CaSSR318	(TC)9	TAGGCATTTGCAACGCTATG	TTTTGCGTTCCTTTTCTCCT	59.9	202	Genomic	Ca6	2111716	
CaSSR319	(TTG)7	TCACCATCGTGTGATGGACT	TTGTTGGGTCCTCTTTTGTTTT	59.9	220	Genic	Ca6	2501203	Expressed protein
CaSSR320	(TTC)5	GCAGCAACATCTGGTGAGAA	CGTTCAGAGGTCTAGGGTGC	59.9	205	Genic	Ca6	2549860	RING/U-box superfamily protein
CaSSR321	(TTA)15	TCCCTTCCCCTCTTTTGTTT	TGTTTGGAAAACTGTGTTTAGAGT	59.9	203	Genomic	Ca6	2766175	
CaSSR322	(AAAGAG)5	GATGCACATCATCTTCTTCTG	GCAATATTGGAAATTCAGTGT	55.8	147	Genic	Ca6	3867508	NAC domain containing protein 75 (NAC075)
CaSSR323	(AT)12	CGTTTTTCATATTCAGGCCC	CGCATGCATACGGAACTCTA	59.4	187	Genomic	Ca6	4048430	

CaSSR324	(AG)7(A)13	CATAAATGGTTGCGACATGC	TCGACACAAACAAAATCACCA	60.0	168	Genomic	Ca6	4400440	
CaSSR325	(TTTTTC)4	ACAACTTACTGTCGTTGGAAA	ATTTCAAATCCTTCGTGAAAC	55.0	147	Genic	Ca6	4566075	Transcriptional co-activator
CaSSR326	(TTTTTC)4	ACAACTTACTGTCGTTGGAAA	ATTTCAAATCCTTCGTGAAAC	55.0	147	Genic	Ca6	4566075	Transcriptional co-activator
CaSSR327	(TA)8	CGGGTAATGAAACTCATAAATTAAA	CAATGCCTGCATTCCAATTA	57.8	274	Genomic	Ca6	5169574	
CaSSR328	(CAA)5	AAGACCAAGTCAAGGACTTTC	GAATACATATTCGTCGTCGTC	55.1	144	Genic	Ca6	5237484	Homeodomain protein
CaSSR329	(ATC)7	TGTTCCTTCTTCATCTTCAAA	TGATCTATTTTCTTGCCACAT	55.1	151	Genic	Ca6	5237688	Homeodomain protein
CaSSR330	(CAA)5	TGTTCCTTCTTCATCTTCAAA	TGATCTATTTTCTTGCCACAT	55.1	151	Genic	Ca6	5237688	Homeodomain protein
CaSSR331	(TCT)8	GGCAGCGACAACTACAACAA	TAATTGAGAATCGGGTTCGG	59.9	199	Genic	Ca6	5259895	BTB/POZ domain-containing protein
CaSSR332	(ATT)8	TCCTTGCCTTGTTCATAGTTA	GGCCCCTGTAGATGATGA	55.2	121	Genic	Ca6	6141841	Putative auxin response factor
CaSSR333	(GAA)8	TAATGAAAATTGTGGGAGAAG	TGACCCTCTTGTCATACTCAT	54.0	153	Genic	Ca6	6540055	Putative protein kinase similar to the calcium/calmodulin-dependent protein
CaSSR334	(TTC)12	ATCACTTCTTGCCATGTCCC	GGTGGTGGAATGAGGAGAAA	59.9	106	Genic	Ca6	6540055	TT viral orf 1
CaSSR335	(AAAT)12	CCGAATTCCCTGTTGTAGGA	ACTGCATGTTTGCCAGGTTT	59.9	276	Genomic	Ca6	6631430	
CaSSR336	(GA)9	TTCACTCAAATgTAGCAATCAAa	AATTATTGCCTGAGTTGCGG	57.5	214	Genomic	Ca6	7376505	
CaSSR337	(AT)8	TGCAGCAGAGCATCAAATCT	GTTGTCTGAAGGTCCCCAAA	59.9	135	Genic	Ca6	7531996	Expressed protein
CaSSR338	(AC)8	GCCACCGTTGTGTTCCTTAT	CGTTTTGAAAAATCGGTGGT	59.8	183	Genic	Ca6	7645002	LIP1 gene small GTPase
CaSSR339	(CT)20	TTTGGTGGTGAACGTTGAAA	GGAGAAGGaAAAGGAAGGGA	60.0	243	Genomic	Ca6	8335176	
CaSSR340	(AAAAC)5	CGTTGCTTAAGATACCAAAAC	AGAGCTTCCAAGTCTCTTCAT	54.4	179	Genic	Ca6	8368375	homeodomain leucine zipper class I (HD-Zip I) protein
CaSSR341	(TGA)9	TCATGGATTTCCTTTGCCTC	CGCAACTTCTCCCTTTCATC	60.0	248	Genomic	Ca6	8816079	
CaSSR342	(AG)10	AGGTAGTTGAAGAAACGAACC	AGAAGCAGGATCAATACCTTT	55.1	153	Genic	Ca6	9118284	Basic-leucine zipper (bZIP) transcription factor family protein
CaSSR343	(TGAT)4	TGATTGTGGATTCTTCGATAC	CAACTAACACCAAGAAGCAAC	55.2	150	Genic	Ca6	10053750	basic leucine-zipper 44 (bZIP44)
CaSSR344	(CAG)8	AATCTCAGCCTCAAACTCAAT	GCTGTTGTGATTTTTGTTGTT	55.6	147	Genic	Ca6	12437191	auxin response factor family protein
CaSSR345	(AC)12	TTCTTTTTGTTTCTCACCAAC	CCTTTGGTGTAAAAGGGTACT	54.6	138	Genic	Ca6	12569883	TEOSINTE BRANCHED 1, cycloidea and PCF transcription factor 2 (TCP2)
CaSSR346	(CCACTA)4	TTGCCATTATCATCTTCTTTC	ATAAATTGCACAGAAGGTGTG	54.6	141	Genic	Ca6	13095585	indeterminate(ID)-domain 14 (IDD14)
CaSSR347	(AACACC)3	AGCTCATATCAAAACATGTGG	GGTTGTAATGTTGTTGCTTGT	55.3	159	Genic	Ca6	13096321	Putative role in shoot gravitropism
CaSSR348	(AGA)6	ATCGCGTGAAGGAGAGAGAG	TACAACACCAACGACGCATT	60.0	141	Genic	Ca6	13949884	Protein of unknown function DUF1084 (InterPro:IPR009457)
CaSSR349	(AC)10	TGGCATCACAAGATTTCAATG	GCACTGTCATGAGAAAGCCA	59.5	251	Genomic	Ca6	14059898	
CaSSR350	(TC)11	CGTTTCTCGCTCTGGAGGTA	TTTCGTTGGTTACACGGTCA	60.0	253	Genic	Ca6	14301000	A-type cyclin-dependent Putative protein kinase
CaSSR351	(AAT)27	TGAAAGTGTTTTGAAAATTAAATGATG	GTGGCTACGGAAGTCTCCAA	59.7	255	Genomic	Ca6	14324174	
CaSSR352	(TTC)13	CTGCATCAACCACACCAATC	CAACGGATAATGCACTCCCT	60.0	136	Genic	Ca6	14759107	potassium transporter
CaSSR353	(ATA)11	TTGAGCACAAAGTTTCCTCT	TCCCTTCAATAGGTAAATTCC	55.2	151	Genic	Ca6	14811478	basic helix-loop-helix (bHLH) DNA-binding superfamily protein
CaSSR354	(AT)11	ATTGTTGGCAGGTTGTGGTT	CCGATTGTAGTGGGGCTAAT	60.3	230	Genomic	Ca6	15710297	
CaSSR355	(TCT)5	CATCAGTAACCGCTCAACGA	TTTGGAGGTGGAAAGAGTGG	60.1	244	Genic	Ca6	16936979	Ribosomal protein S5 family protein
CaSSR356	(TA)10	ATCAGCACCTCATACCCGAG	CGACAAGCCCAGACTCATAA	60.1	242	Genomic	Ca6	17580949	
CaSSR357	(TAT)9	TATCCCAAAAGTCACAATTCA	ATATGCAACACACAGAGGAAG	55.7	141	Genic	Ca6	18298837	ERF (ethylene response factor) subfamily B-3 of ERF/AP2 transcription factor family
CaSSR358	(AT)15	TTTTTCATCCATCACATCATCA	TTGATGCTTTACAACGTCGC	59.9	261	Genic	Ca6	19858207	F26K24.10 protein
CaSSR359	(ATT)15	CGGTTACCTATTTTTAATTGTGC	TTCTCCATCACACTGGGAGA	57.0	247	Genomic	Ca6	20442770	
CaSSR360	(TTGATA)3	GTGGAAGTGAATTCAAGTGAG	GTCCAACACCATAACAATCAT	54.8	148	Genic	Ca6	20514111	SET domain protein 35 (SDG35)
CaSSR361	(TTCACA)5	CCCACTTTCTCACTCACTCTT	TTCTGGAGGAGATAGTAGTGGT	56.1	163	Genic	Ca6	22249411	GRAS family transcription factor
CaSSR362	(CAA)8	ACCATTTGTTTGGGCATTTC	CGAATTGAGGGTTCTTCCAA	60.0	274	Genic	Ca6	23920468	NTMC2T5.2
CaSSR363	(AGA)4	GGATTAATTATCTCCGACCAG	TGGTAATCTTCCAGCTATCAA	55.4	122	Genic	Ca6	24448426	the R2R3 factor gene family
CaSSR364	(TC)6	ACCAACTCACATCTAGAAGCA	TGATCTTCCCATCTTATTTCA	55.0	157	Genic	Ca6	24448740	the R2R3 factor gene family
CaSSR365	(TAG)6	TGCAAACCACTTTTCCTTCTG	CGAATCACAAAATCCATCCC	60.1	140	Genic	Ca6	26357912	GRAS family Protein, transcription factor

CaSSR366	(TAG)7	AAGCCTTCTATATTGCAAACC	CAAAATCCATCCCTAGAACTT	55.4	146	Genic	Ca6	26357925	GRAS family Protein, transcription factor
CaSSR367	(ACT)7	TGGATAACCCTTTTCTTCTTC	AGCATTTGGTTTATGTAACGA	55.1	145	Genic	Ca6	26358317	GRAS family Protein, transcription factor
CaSSR368	(AGAA)5	GTGATCTGTCATCGTTTCAAT	AGGAAGCTTTGAGAAAGAGAA	55.0	141	Genic	Ca6	26358709	GRAS family Protein, transcription factor
CaSSR369	(AG)6	GCTATCGCCGATCAAGAGAA	TTCTAAGCTTTGGAAGAGATCCA	59.5	236	Genic	Ca6	26765102	Ras-related small GTP-binding family protein
CaSSR370	(ATT)8	AAAAaCCCGTGTCGTTTATTT	AGGGCAATATGCGATTCATT	57.6	256	Genomic	Ca6	26878517	
CaSSR371	(AT)14	CCATGCTTGTGACGTTCTCT	GCCTTTTCtTTTTCTTTGGGA	58.9	279	Genomic	Ca6	29119837	
CaSSR372	(GTG)4	GAGGAAGTTACCAAAAGCAAC	GGTTACATGTTTTGCATTCAT	55.7	162	Genic	Ca6	29256025	C2H2-type zinc finger family protein
CaSSR373	(TAT)32	TtCACTTGTTCAAGTACAACTATTtCA	GGAATGTGCCAACATCAGAA	58.5	273	Genomic	Ca6	30258394	
CaSSR374	(TA)10	GCGTTAGCGTCCTTGACTTC	TGAAGTGGTATATCTTAACCCAGGA	60.0	257	Genomic	Ca6	30465376	
CaSSR375	(AGA)7	GACCTCGAAATTGAGAAGCG	GCATCGTCATCTTTGCTTCA	60.0	109	Genic	Ca6	30580648	DNA-binding protein that binds to plastid DNA non-specifically
CaSSR376	(AAT)7	AGCGATTACCTTGAATGTGAGA	TCACACACATGGACCGAGTT	58.9	273	Genomic	Ca6	31079517	
CaSSR377	(TA)10	GGAGACAACTTCTCTTCACCAA	AGCCACTTCATTGACTTGTCTC	58.4	203	Genomic	Ca6	32082396	
CaSSR378#	(ATT)9	CATTCCCATATTTTCCTCCG	AAGACAATCGAATCCAACGG	59.9	266	Genic	Ca6	32200864	Expressed protein
CaSSR379	(GAA)6	TGTTCTGAATTCTGTTTCAGG	ACTTCCTCTGTCTATGCAGGT	55.4	150	Genic	Ca6	37387965	ovate family protein 4 (OFP4)
CaSSR380	(CTA)8	TGTTGCTCcTTTCTTTGCCT	GAGCATGTGGTGAAGCAGaA	60.0	152	Genomic	Ca6	37974670	
CaSSR381	(AG)12	TACCACTTTTATACGCTGCAC	ATGAATGAACGAATGTGACTC	55.7	145	Genic	Ca6	38814576	HAIRY MERISTEM 3 (HAM3)
CaSSR382	(AT)11	TTTTtGTTTTtGTCAATGTAATATGC	AAGCGAGAACTTACGGAACC	58.6	244	Genomic	Ca6	41910782	
CaSSR383	(GGA)7	TTTTGGAGGAGGAGATATAGG	AGTCATTTGTAATGGCATCC	55.1	163	Genic	Ca6	42715963	Dof-type zinc finger DNA-binding family protein
CaSSR384	(TA)11	CCCATAGAGAGCCCAACAAG	GGTCCtCTTTCTTCCGTCG	59.7	252	Genomic	Ca6	43988772	
CaSSR385	(AC)7(AT)6	CTTTCCATTTCCCATCACAA	AAGTCCTTGCAAACAAATCTCC	58.4	218	Genomic	Ca6	47487429	
CaSSR386	(TA)10	TGAAAATTAACGTACGGCACA	GATGACTTGTTCTCGTCGCA	59.1	153	Genomic	Ca6	49250840	
CaSSR387	(CT)15	CACATGTTCTTGCAACCCTG	ATTCaAAACCCaGAAAGCCC	60.2	154	Genomic	Ca6	50249027	
CaSSR388	(AG)16	AAACGCCTTGTCACCTTTTG	AATgGGGTGTTGAtTTTGGA	60.1	233	Genomic	Ca6	51773961	
CaSSR389	(AT)9	CCCtGCACTACACGAGGTCT	AAATACGTGTCTGCACGTCG	60.3	277	Genomic	Ca6	52161279	
CaSSR390	(TTA)7	CTCCTCAACCGCTGCATTAT	TGGTGGTTCTTCTTCCTACGTT	60.2	259	Genomic	Ca6	53565894	
CaSSR391	(CAA)7	TCAACTGTTCAACAACATCCT	GAGGAGGAGCACAAGAACTAT	55.6	163	Genic	Ca6	53652536	basic helix-loop-helix (bHLH) DNA-binding superfamily protein
CaSSR392	(AAT)6	CGGTAAGAGAAGAAGCCACG	TGCATTCAATTCAATTCCACA	59.9	280	Genic	Ca6	56766583	putative galacturonosyltransferase activity
CaSSR393	(GAA)6	GAATGGGAGGAAGTGGTTGA	CAACCCAACACCCCTCTCTA	60.0	198	Genic	Ca6	56796755	RING/U-box superfamily protein
CaSSR394	(AC)11	ACCGAAGAGTTTGATGGACG	GCTGCACTCATGGATGTGAC	60.1	137	Genomic	Ca6	56978935	
CaSSR395	(AT)7(AG)8	TCACCAACTCGTTGTTTACCTTT	TCAATAAACAAATTCAACATTCAAG	60.0	271	Genomic	Ca6	57785268	
CaSSR396	(AAG)6	TCGAGGATCTGATGAAGGAAG	TTCAGTTTCACGCAATCACC	59.7	236	Genic	Ca6	58296246	Major facilitator superfamily protein
CaSSR397	(TGTTCA)4	GAATCACAGCCATTAACCAT	GACAGTGTTGGAAAGACAAAG	55.0	147	Genic	Ca6	58334307	transcription factor CIB1 (cryptochrome-interacting basic-helix-loop-helix)
CaSSR398	(TA)6	TTGCTTTGAAGAAGGGGAAA	CCACTGCTGTCCTACACTGC	59.5	155	Genic	Ca7	1283953	Expressed protein
CaSSR399	(AGA)7	AACGATTCCAAAAGTTACGAT	GGGTTTCTGAATTAGGTTCAT	55.5	145	Genic	Ca7	1371794	WRKY Transcription Factor
CaSSR400	(CT)6(CA)7	TTTACACCCCCTAATTCCCC	CAATTTCGGCCAAGAACATT	59.9	151	Genomic	Ca7	1691706	
CaSSR401	(TC)8	CGATCAGTTAAAACCGCACA	TCTTCGACGAAACAAAGCAA	59.7	241	Genomic	Ca7	2219240	
CaSSR402	(TTA)7	GGGACAAGTCAGTCCGGTAA	ACACCACCCACCAGTAGGAA	60.3	196	Genic	Ca7	2222564	Expressed protein
CaSSR403	(AAT)7	ACACCACCCACCAGTAGGAA	GGGACAAGTCAGTCCGGTAA	60.0	196	Genic	Ca7	2222759	Syntaxin/t-SNARE family protein
CaSSR404	(TTC)7	AATGGAAGTGGAAGTAGTGGT	CTCCAATCCAATTTCCATAAT	55.3	139	Genic	Ca7	2334917	AP2 family of transcriptional regulators
CaSSR405	(TAT)5	CGCCACAACAACACCATATC	CGAAAGAAATTGATGCGAGA	59.0	202	Genic	Ca7	2944747	casein Putative protein kinase 1 protein family
CaSSR406	(TC)12	TCACTCCCTCGATCCTCAAC	AGAAACTTGGCAAAAGCAGC	59.6	280	Genic	Ca7	3046324	MAP Putative protein kinase Putative protein kinase 2
CaSSR407	(TGC)4	AGATTTTGTGATTCAGTCAGC	TCAACAGCAACAACAGTTACA	54.5	174	Genic	Ca7	3356103	Putative auxin response factor
CaSSR408	(AAG)10	CGGCCATTGAAATTGAAAA	GTTGGAAACAAACACGCCTT	60.0	276	Genic	Ca7	3393987	plastidic beta-ketoacyl-ACP synthase II

CaSSR409	(TCT)4	CAAATTGATGCTTATCAAACC	AGAAGGAGGAGAGATGCTTAG	54.9	159	Genic	Ca7	3750508	the Aux/IAA family of proteins
CaSSR410	(TA)8	AACGCTCCAACGTCTTAAAAT	ATGTATGAAACAAACAAACACGA	58.0	266	Genomic	Ca7	4219629	
CaSSR411	(CT)10	ATTCTCCTCATAAGCCATTCT	TTCTCGATCTATACGTCAACC	54.7	150	Genic	Ca7	6057942	A Class II KN1-like homeodomain transcription factors
CaSSR412	(ATTC)6	AGAATTCACAACCCACCGAC	ACATCACCTCCTCCGTTCAC	60.0	240	Genic	Ca7	7785589	serine/threonine protein Putative protein kinase.
CaSSR413	(TC)11	CCGATACCGGAGGATGTAGA	CCCAAACTCGACCTTGTTGT	60.0	239	Genic	Ca7	8489146	HNH endonuclease
CaSSR414	(CTT)7	ATCACAACGTGGTGGTAGCC	TCTTCTTCTCCTGGGGATCA	59.7	265	Genic	Ca7	8615603	TCP family transcription factor
CaSSR415	(CTT)9	TCGACATGATAACACAAGTCA	TAAGATCAAACAAACCTCACG	55.0	163	Genic	Ca7	8615627	TCP family transcription factor
CaSSR416	(TAT)7	CATCAACGACGACGAGAGAA	AACACTTGGCCGTTGGTTAC	60.0	214	Genomic	Ca7	8921807	
CaSSR417	(AGA)6	GAGGAGGTGAATTTGGCAGA	TGTTGGTTCTATTAACCCCCAT	59.5	217	Genic	Ca7	8979287	Dynein light chain type 1 family protein
CaSSR418	(AAGAAC)3	ATACTTCCCCTGAAGAAGATG	CCACGAACTCTCTTAACCTTT	55.0	142	Genic	Ca7	9867339	C2H2-like zinc finger protein
CaSSR419	(AT)11	CCACATCCACAAACACAACA	TGTTCACCCAAACCAACTCA	58.8	267	Genomic	Ca7	9969630	
CaSSR420	(AGA)12	GGAGCTGTGAACGGTGAAAT	CGAAACGCACCCTATTTTGT	60.1	207	Genomic	Ca7	10031195	
CaSSR421	(TGAATT)4	TGAACTATCCCCTTTAGGAAC	GAAGTTGATGAGTTTGATTCG	55.0	153	Genic	Ca7	10115566	ERF (ethylene response factor) subfamily B-3 of ERF/AP2 transcription factor family (ATERF-5)
CaSSR422	(TC)7	CCCAGAAAAGAGAAAACGCA	ATTTCTCAACTGTCTCGCCG	60.4	269	Genic	Ca7	10296770	Inositol phosphorylceramide synthase
CaSSR423	(GTG)4	AAGAGGTTCAAGAATTGAAGG	AGCCATAGAGAAAGTGGTTTT	55.2	149	Genic	Ca7	11192806	homeobox protein HAT22, the HD-Zip II family.
CaSSR424	(AC)8	CGTGGGACGAACTGAGTCTT	AGTTTGGCGCTGAATTTGAC	60.3	230	Genomic	Ca7	11425179	
CaSSR425	(AGA)6g(A)10	TTCTTCCTTAGAGCACAAACCC	TCCTCTCTCTCCGTTACCCA	59.8	190	Genomic	Ca7	11656778	
CaSSR426	(AT)7ag(T)11	TTCGTTTTTCATTTTTCTTCAGC	ACAACGTCGCACTAAAACGa	59.8	172	Genomic	Ca7	11960437	
CaSSR427	(ACACAA)4	CCTCTCTTATTTTGTTTGTCAAC	TGAAACTGTCGGTTCTGTAAC	54.9	165	Genic	Ca7	12021104	WRKY DNA-binding protein 15 (WRKY15).
CaSSR428	(TA)10	TTTGCAAATGGCTCAcTCAT	TTCAAAATGAAATATTCTTCTTCCG	59.3	233	Genomic	Ca7	14610251	
CaSSR429	(TAT)5	AAACTTGAGGGCAAGCCTTT	CAAAACACAGAGTTGAAGGAACA	59.3	103	Genic	Ca7	14738300	flavanone 3-hydroxylase
CaSSR430#	(AG)12	AAGCAGAGACCCATGTGGAG	AGTGGGGAGGGAAACAAACT	60.3	233	Genomic	Ca7	15721218	
CaSSR431	(ATGG)3	AGGTTTACCATCAGCTTCACT	TCCTTATGTTGCAATGTATCC	55.6	157	Genic	Ca7	17777145	G group bZIP transcription factor family member
CaSSR432	(ATGG)3	AGGTTTACCATCAGCTTCACT	TCCTTATGTTGCAATGTATCC	55.6	157	Genic	Ca7	17777145	G group bZIP transcription factor family member
CaSSR433	(TTC)7	GTCTTGGGAACCTTGCGTTA	GATGCGTAACTACAACCGCA	59.8	160	Genic	Ca7	18618008	Tetratricopeptide repeat (TPR)-like superfamily protein
CaSSR434	(ATT)28	TGAACAACTCAAATAAACAATCTTCC	TCTCACTAATCCaAAACaCCaAAA	59.8	257	Genomic	Ca7	19594142	
CaSSR435	(CT)18	ACCGCCTCAATTTCTTCTTA	TAATTTCACGGACAGTGTCTT	56.6	148	Genic	Ca7	19831349	PLATZ transcription factor family protein
CaSSR436	(TTC)7	AGTGATTCTCTGTGTCTCTGC	AGAATAGAAAACGGCAATGTT	54.5	155	Genic	Ca7	19942079	basic helix-loop-helix (bHLH) DNA-binding superfamily protein
CaSSR437	(TTC)7	AGTGATTCTCTGTGTCTCTGC	AGAATAGAAAACGGCAATGTT	54.5	155	Genic	Ca7	19942079	basic helix-loop-helix (bHLH) DNA-binding superfamily protein
CaSSR438	(GAA)8	AACATCATCATCAAGTTGGAG	TGAAGAAGAAGCACTTAATGG	55.1	154	Genic	Ca7	20120406	putative transcription factor; KANADI family
CaSSR439	(TAT)6	GGTTTGTTCCCTTTTACTTGT	CTCTTCAACTGAACCCTACCT	55.0	141	Genic	Ca7	20231155	Floral homeotic gene encoding a MADS domain protein homologous to SRF transcription factors
CaSSR440	(TA)11	TATCTCCACTCCCTCGTTCC	TGTTCTAGCAGGACCCTTCC	59.1	197	Genomic	Ca7	21614939	
CaSSR441	(AGC)4	CTGGTGTATTTTTCGTCAGTC	GATTCAAAATAGGGGTCTCAC	54.9	153	Genic	Ca7	21805504	Putative transposase
CaSSR442	(TTGGAA)3	ATGATGTTGGTGTTTCAAGAG	TAATATCACTGCGAAATCCAC	55.1	159	Genic	Ca7	24021097	homeobox protein 34 (HB34)
CaSSR443	(AT)13	CCGCAATCATATCAACTTtGTG	CCCTTCCAATTCTTCAATCC	60.4	243	Genomic	Ca7	24412796	
CaSSR444	(AT)6(AC)6	ATGCAAAGGTGTTTCGTGTG	CAAAGTGAGGATGAACCAATTTC	59.6	229	Genomic	Ca7	27443030	
CaSSR445#	(AG)14	AGCGGTAGGTACTTAAGAAGG	AAGCTGTCTCCTAAATGGAAG	54.7	155	Genic	Ca7	28547368	SBP-box gene, a the SPL gene family
CaSSR446	(AATT)6	GCTCCTCCATTAACTCATTTC	CAACTTCAAGCTCAATGTTTT	55.5	168	Genic	Ca7	28548004	SBP-box gene, a the SPL gene family
CaSSR447	(AG)6(A)12	TTGAATCGAAATGGGTGTGA	cCCCTTTGCCTTTATTCCAT	59.9	197	Genomic	Ca7	29567814	
CaSSR448	(AT)21	TCGGATTAGTGCATGaGAAAa	GGATAATACGAgTTTGTTCGCAA	58.4	205	Genomic	Ca7	30653052	

CaSSR449	(TC)12	CCCCTCcATCTACTGTTTCC	AAACCTAGCTTGGCAAAAACC	58.4	252	Genomic	Ca7	31192294	
CaSSR450	(TA)10	GTGACGGCCTCCTTAACAAC	TTTTGGTGGAACACTTGCCt	59.6	264	Genomic	Ca7	31642295	
CaSSR451	(TTGTGA)4	TTTGAAGGTCTTGTTGTTGTT	AGTGCAAGAGCTATGACAAAC	54.9	151	Genic	Ca7	31893675	indeterminate(ID)-domain 11 (IDD11)
CaSSR452	(TTGAGT)3	GACAAAGCTATAACCTCAGCA	CAGGAAACAGAACTGAAATTG	54.9	148	Genic	Ca7	31894863	indeterminate(ID)-domain 11 (IDD11)
CaSSR453	(TTTGAT)6	CAAGAAGGTGAAGTTAAAGCA	TCTTCAACAACAACAACAACA	55.0	155	Genic	Ca7	32855952	Growth regulating factor, transcription activator
CaSSR454	(TTTGAT)6	CAAGAAGGTGAAGTTAAAGCA	TCTTCAACAACAACAACAACA	55.0	155	Genic	Ca7	32855952	Growth regulating factor, transcription activator
CaSSR455	(TC)8	TCCAATGAGGTTGTGACGAA	CGGAGATGAATTTTCCGAGA	60.1	201	Genomic	Ca7	33218307	
CaSSR456	(AAT)9	CAAACGGAGAGGGATGAAGA	TGATCCATGCTTTTCCAAAT	58.0	270	Genic	Ca7	33782743	Putative 1-phosphatidylinositol-3-phosphate (PtdIns3P) 5-Putative protein kinase
CaSSR457	(ATG)4	CTTCTCCATCAACAACAAATC	CTTGTTTCCAAGTGACAATTC	54.8	161	Genic	Ca7	34320789	NGATHA3 (NGA3)
CaSSR458	(ATC)7	ACCAACCCTACAAGTCTCTTC	GTTATGCATCTCATGGTGAAC	55.0	142	Genic	Ca7	34923150	protein containing Dof zinc finger motifs
CaSSR459	(ATC)4	TCTTGAATCCAGCAGATTTAG	TGTATTTCTCTTTTCCTCGTG	54.8	150	Genic	Ca7	35180540	basic helix-loop-helix (bHLH) DNA-binding superfamily protein
CaSSR460	(TA)11	ACTAATGCCAATCGCTCAGG	ATGTGATGCGTGGTTGAGTC	60.2	267	Genomic	Ca7	35187940	
CaSSR461	(CAAGCC)3	ATGTCCATGCAAGTAAACAAG	CGACGACCTCTACCTTCTACT	55.3	137	Genic	Ca7	38472298	TCP family transcription factor
CaSSR462	(AAT)45	GTCATTTTCAACTGACTCATATTCAT	TTCTATGGaAAcCCAGTGAGC	57.8	247	Genomic	Ca7	42333636	
CaSSR463	(ATG)5	TACGGAGGCTATGGATGGTC	CACTCTTCCACTCCTTTGTGC	59.9	194	Genic	Ca7	48439099	Expressed protein
CaSSR464	(AAT)24	TCACCTTCATTGTTGGTCGT	TTGTAAATTGTGGCACCCAG	59.0	256	Genomic	Ca7	48752339	
CaSSR465	(TTC)5	CCCTCTTCTCTACGACACCG	CGGTGGaCATCCTCGTATCT	59.9	279	Genomic	Ca8	1930653	
CaSSR466	(AG)6	TGTCAAAAGCGAGAATTCCA	GGAAGCAGCTGGTTTTGTTG	60.8	280	Genic	Ca8	2001637	protein with hydroxymethylglutaryl-CoA synthase activity
CaSSR467	(ATA)7	CAAAGGGGAAGAAGTGTTGC	GCCATTTCTGGAGTTGGAAA	59.7	219	Genomic	Ca8	2524466	
CaSSR468	(TGAT)3	GCTCTAAGACCCAATTGTTTT	GAATCAGTGATGAAGAAGACG	55.2	151	Genic	Ca8	2557055	homeodomain-leucine zipper protein
CaSSR469	(CAA)7	TTTCAACAATGCCAACCAAA	TCGAAGAAGGGGAAGAAACA	59.8	211	Genic	Ca8	3154215	basic helix-loop-helix (bHLH) DNA-binding superfamily protein
CaSSR470	(AT)6	TTTTCAAAGAACCCCAACAGA	AGCAGGACATTTGTGCAGTG	59.9	159	Genic	Ca8	3763063	Chaperone DnaJ-domain superfamily protein
CaSSR471	(ACT)7	CAAAACCCCATAATGCCACT	TTAGCTGAGCTGTTTGGCG	60.3	205	Genic	Ca8	3945750	Phototropic-responsive NPH3 family protein
CaSSR472	(TGA)5	TCCAATTCCTCACAACCACA	CTCTTAACTCCGCCGTCAAC	59.9	197	Genic	Ca8	4243742	Homeodomain-like superfamily protein
CaSSR473	(TTA)9	AAGTTGAGGAAGGGGATGGT	TAGTGGGCTTCGTATTTGGG	59.8	274	Genomic	Ca8	4270429	
CaSSR474	(TGT)7	ATATTTTTGTTGTTGGTGTCG	GAAGCCAATGGTAGAAGAAAT	55.2	155	Genic	Ca8	4623460	BEL family of homeodomain proteins
CaSSR475	(TGT)7	ATATTTTTGTTGTTGGTGTCG	GAAGCCAATGGTAGAAGAAAT	55.2	155	Genic	Ca8	4623460	BEL family of homeodomain proteins
CaSSR476	(AG)6aaagaga(GT)(	CGCGAAGAGAAAGAGACGAG	TGCAGGGTATGAAAGGATGA	60.4	141	Genomic	Ca8	4628934	
CaSSR477	(TTC)4	TAGATCTCACACACACAATGG	GTTATGGTGTGAATTTTGAGG	54.4	143	Genic	Ca8	5196644	homeobox protein 24 (HB24)
CaSSR478	(ATT)4	TCTACAGACACAAAAGGGGTA	CACTTATCTGTCGCACTCTTT	54.9	150	Genic	Ca8	5411626	SHI gene family protein
CaSSR479	(AG)7	CACAAAAGGGGTAAGTGGTCA	CGCATTCAACTCATTCGTGT	59.7	232	Genic	Ca8	5411634	SHI gene family protein
CaSSR480#	(TTGAG)4	AATCCAATCGAAGAGAGAGAG	ACTCTAGGGTTTTCACTTTCC	55.3	154	Genic	Ca8	5569895	DNA binding
CaSSR481	(GAA)6	TGCAAGTTAACACGAGCACC	TTCCAGCAAATTTTGGAACC	59.9	189	Genic	Ca8	5822343	Chalcone-flavanone isomerase family protein
CaSSR482	(GGT)5	TCCTCTTCGACAATTCGCTT	GAAGGAATTGGTGCTTTTGG	59.5	173	Genic	Ca8	6160737	Expressed protein
CaSSR483	(TCT)8	TTCAATCTCTTCACGCCCTT	GAAGGTTTCGAGTGCCTCAG	60.0	160	Genic	Ca8	6688803	Pentatricopeptide repeat (PPR) superfamily protein
CaSSR484	(AAG)8	GAACCAAACATGCACAATGC	TCCTGCGTATCTGTGTCTTTGT	60.0	278	Genomic	Ca8	7731588	
CaSSR485	(CTA)7	CACAAGGGTTTATGATAGTGC	CCATGATAAGAAGACAACCTG	54.9	150	Genic	Ca8	8364902	homeobox-7 (HB-7)
CaSSR486	(AGA)4	TGAGATTGGTGTAAAGAAAGG	AATTTGTCCACCTTAGCCTAC	54.6	155	Genic	Ca8	8639326	the R2R3 factor gene family.
CaSSR487	(AATAAC)5	TCACAGAAGCAGAATCAAAGT	CAAAACTCAGAATCTCACCAG	55.2	148	Genic	Ca8	8841425	MYC-related transcriptional activator
CaSSR488	(AT)9	AGGCCAGTTGACACCATAGC	GCTGGAACCATTTCCAATGT	60.1	171	Genomic	Ca8	10925248	

CaSSR489	(TA)10	TTgaATATTGTGTTTGATTTCCTTT	CCCTCATATTCAAAACAATCCC	58.1	219	Genomic	Ca8	11159303	
CaSSR490	(AT)10	CAAATCAATATACAACACATCACTCC	CTTGCTCCTACGTATCCCCA	58.8	215	Genomic	Ca8	12030526	
CaSSR491	(TA)10	TGTCTTCAAAAGGATAACTCATGC	TCATTTTGAGTGTTGAAGATTATgG	59.7	207	Genomic	Ca8	13144169	
CaSSR492	(ACA)5	CCCAGATACAATGCATACGCT	CCGATTTTCCTTCCTCAACA	60.0	222	Genic	Ca8	14017713	Expressed protein
CaSSR493	(CCT)5	CATCATCTGTTGCCTCTTAGT	ATGAAGATGATGATTTCGATG	54.5	167	Genic	Ca8	14955395	Alcohol dehydrogenase transcription factor Myb/SANT-like family protein
CaSSR494	(ATCAAA)4	AAGTACTGTGAATGCTTCCAA	CTTGCTGAATATACGTGTGGT	55.1	156	Genic	Ca8	15220963	Tesmin/TSO1-like CXC domain-containing protein
CaSSR495	(ATCAAA)4	AAGTACTGTGAATGCTTCCAA	CTTGCTGAATATACGTGTGGT	55.1	156	Genic	Ca8	15220963	Tesmin/TSO1-like CXC domain-containing protein
CaSSR496	(TCT)4	TAAATCGCCTAGAGATGTTCA	CATAACTTGTTCCGAGTCTTG	55.2	155	Genic	Ca8	15607448	DREB subfamily A-4 of ERF/AP2 transcription factor family

*CaSSR *Cicer arietinum* SSR

seven informative genomic and genic microsatellite markers discriminated all the 94 cultivated and wild *Cicer* accessions from each other
