# Supplementary material for: Natural Allelic Diversity, Genetic Structure and Linkage Disequilibrium Pattern in Wild Chickpea
Source: PLoS One. 2014 Sep 15;9(9):e107484. doi: 10.1371/journal.pone.0107484 (PMC4164632; doi:10.1371/journal.pone.0107484)
Supplement: Table S3 — Summary of 384 TF gene-derived SNPs genotyped in 94 annual/perennial cultivated and wild Cicer accessions using high-throughput MALDI-TOF mass array. (PDF) [file pone.0107484.s010.pdf]

Table S3: Summary of 384 TF gene-derived SNPs genotyped in 94 annual/perennial cultivated and wild *Cicer* accessions using high-throughput MALDI-TOF mass array

SNP IDs*	1st Pre-PCR primer sequence	2nd Pre-PCR primer sequence	Unextended Primer sequence	SNP allele (ICC 4958)	SNP allele (ICC 17160)	Size (bp) of alleles amplified	Actual annealing temperature (0C)	Chromosomes	Physical positions (bp)	Transcription factors (TFs)
CaTFSNP1	acgttggatgTCCCAACAAGGAAGCTAGTG	acgttggatgTCAGGTTCCTTCTTAGTTGC	AGCGTTCTTAGCAGGACCTTCATTA	C	T	98	49.1	CaChr1	1661476	GeBP
CaTFSNP2	acgttggatgTCAACAAGACAGTTCGAAGCA	acgttggatgCATCAGCGTGTCTTTTGAGG	GTTCCGAAGACAGGATGTAC	T	C	108	59.62	CaChr1	1827593	Jumonji
CaTFSNP3	acgttggatgCACTGGAGGTGTTGCTTTCA	acgttggatgTTCTCCCCAACAGAACCAAC	CCCCAACAGAACCAACTGAA	G	A	199	59.87	CaChr1	2188544	SNF2
CaTFSNP4	acgttggatgTGATCCTCCAGCTTTTGACC	acgttggatgAAAGGTGGGGTGAGGAAAAG	AAAACCTCTTCTTCATCTTCATCATC	A	T	158	60.2	CaChr1	2602506	SBP
CaTFSNP5	acgttggatgGAGGAGGAAGTAGAAACAATAGA	acgttggatgTCCTCCTCCTCCTCTTCT	AGAGGAGGAAGTAGAAACAATAGA	G	A	101	53.77	CaChr1	2631901	C3H
CaTFSNP6	acgttggatgTGACTGCCAAGGAACTCAAA	acgttggatgTCCCTCTTCTGCATGTATTCAA	GGATTCGTTCCAAAAAGAAC	G	C	115	59.41	CaChr1	3650162	MADS
CaTFSNP7	acgttggatgAAAGGGAAGCATCAGGGAAT	acgttggatgTGCTAAAATCCATGGGATGA	GCCAAATGAATAAAAGGCAGAA	G	A	201	59.9	CaChr1	3734533	Bromodomain
CaTFSNP8	acgttggatgGCCTTGCTCTGCCCTAATCT	acgttggatgTTGTGAAAGAGGGGTTTTGG	TGCTCTGCCCTAATCTCAAC	G	C	109	60.87	CaChr1	4325063	mTERF
CaTFSNP9	acgttggatgCCCCCTCTCAATGTTTTTGA	acgttggatgTTTCATCATGGAGCCGAAA	CATAATCATTTAGTTCCCTGTATCTTTCT	T	C	197	59.9	CaChr1	4379384	SNF2
CaTFSNP10	acgttggatgGGGTGTTGTTGCTCCTGAAT	acgttggatgTCTTTGGACGATTCACTGCT	CATCTAATGTTGCTGTAACTGTGAGT	G	T	228	59.97	CaChr1	4866342	BSD
CaTFSNP11	acgttggatgGCAACTTCTTATTTAATTAT	acgttggatgCCATTTAGTAGATTTTCATC	CATATAATATTTATCCTAAAAATGCAAG	C	G	109	46	CaChr1	5452945	MYB-related
CaTFSNP12#	acgttggatgCAAGGAACCAAAGAAGAAAAGG	acgttggatgGGTGCTCATGGAAACAACAA	GAAAAACTACTAATTGTATTTCTGCTG	T	C	103	59.28	CaChr1	5666051	WRKY
CaTFSNP13	acgttggatgCCCGTGAAAACTGGGATTTA	acgttggatgAGTCACCCGAACAAATCGTC	CCTGGGCATACTCACCTGTA	G	A	206	59.79	CaChr1	6592726	FHA
CaTFSNP14	acgttggatgGGTGCCAAAGTTGCTTCAAT	acgttggatgGGATCAGTTCAAAGGGTTGC	TTCAGAACTGAGTCCTCCGC	A	T	199	60.12	CaChr1	7417345	HB
CaTFSNP15	acgttggatgATCCAATGGCCAATCTTCAG	acgttggatgAAATGTCGAGACTCGTGCAA	TGCAGGTGTTCCTTTGCC	G	A	199	59.89	CaChr1	7436472	CCAAT
CaTFSNP16	acgttggatgTGTCATGCAACCTAAACACCA	acgttggatgGTTACTTCCAACCGGCACAT	TTTGGTACACAAGTTTCACCTATCTC	G	C	206	60.02	CaChr1	8284578	MYB-related
CaTFSNP17	acgttggatgCTCCGAATACAGAAGCCATC	acgttggatgAGTACAGCCCTTTACTTCGC	CGAGACCCTTCTAAACC	G	A	100	46.3	CaChr1	9654075	Trihelix
CaTFSNP18	acgttggatgGAGTCTGCAACCGCAATTTT	acgttggatgTGGCAATGATGACATGATGA	GATGATCTTGTTGACAAATACC	G	C	117	60.26	CaChr1	10455472	MYB
CaTFSNP19	acgttggatgACCTTGTCGCTCGTGATTCT	acgttggatgGATGGAGGGTGGTGGTCTTA	TGAATTCAAATTCGAATAACTCC	G	A	115	59.87	CaChr1	11285630	GRAS
CaTFSNP20	acgttggatgGGGAGCTTGTGAGGATTTGA	acgttggatgGTACTGCTTCCCCGGTACAA	GGATGGAGCGAAACAGAGTGT	G	A	197	60.2	CaChr1	12530205	AP2-EREBP
CaTFSNP21	acgttggatgCACTGCAAGTAGCACACTCACA	acgttggatgGGAAGCTGGCAAGAAGAAGA	ACAAAAAACATAACATTCACATAA	G	C	110	60.16	CaChr1	12928919	G2-like
CaTFSNP22	acgttggatgATTTCCCAATGCAGTCGGTG	acgttggatgAAAGTTCAGGCGCGCACAAG	CCATACATTGCCCTTGC	C	T	102	48.6	CaChr1	13679988	AP2-EREBP
CaTFSNP23	acgttggatgTCTGCTGGTTCATCCTGTTG	acgttggatgTGTTCGTGTGAGGAATCCAA	GCTCTATGATTATGGATTTGA	T	C	118	59.83	CaChr1	13801427	bHLH
CaTFSNP24	acgttggatgTTGTCTGGTGAAGTTGCTTGA	acgttggatgCCCGAACCTTAATCCTCTCA	CCTAAGTTCCCTACACCTCCTC	T	C	203	59.47	CaChr1	13994815	HB
CaTFSNP25	acgttggatgCATTTCGGAGAGAAGGAGGA	acgttggatgTGCTTCAAGTCCTGAAACCTC	CCAACATTACTAAGATGGATAAGGC	A	C	178	59.36	CaChr1	15378650	bHLH
CaTFSNP26	acgttggatgACTGTCCATCCCGAGGATCT	acgttggatgCGATTCATCTTCCTCCCTCA	GCGTTCTTGTAAGCCCGAT	A	C	201	60.88	CaChr1	15606770	WRKY
CaTFSNP27	acgttggatgGCACCCGATGATAACGAAGT	acgttggatgTCCTCCAACTTTCGTCGTTT	GGGCGAGAGTGTCAGAGAGAC	G	T	200	59.96	CaChr1	16080907	GRAS
CaTFSNP28	acgttggatgAGCTTTCCAATACCCAGATCC	acgttggatgCGCAAAGTAGCTGGTCATCA	TTCAGTCCAAGAGATAGGAAGTATCC	T	C	192	59.43	CaChr1	16680465	NAC
CaTFSNP29	acgttggatgGATGAGGCTTGCTCATTTGC	acgttggatgCTCTTTCAATGCGGTTGGTG	AATGCGGTTGGTGATATTTTACTAGC	T	C	105	54.2	CaChr1	17405553	CAMTA
CaTFSNP30	acgttggatgGGGGGCTAGTCTGGAGAGAA	acgttggatgTGGCATGTGCAAGATTGAGT	AAACATAGTATCAACGACTACAAATGGA	A	C	209	60.73	CaChr1	18460430	mTERF
CaTFSNP31	acgttggatgAGGAGGAAAAACCAGGCTGT	acgttggatgGGCACTCTTTCTCTCCCAGA	GGCCAGACAGAATGCAAGTATTA	T	C	211	60.11	CaChr1	20598392	C3H
CaTFSNP32	acgttggatgACCTTTCCTGTTCTCTGAGC	acgttggatgAAATCGAACGTAGCCTAAGC	CGGGCAAGAAAGAATCAATTGTTCA	C	A	107	52.8	CaChr1	21135977	MADS
CaTFSNP33	acgttggatgTGCAGCATCCTGAATCTGAG	acgttggatgAAATGGGTGTCGGAAATCAG	CGGGAAATTAAACTTAGCACCAC	T	C	204	60.1	CaChr1	23850218	AP2-EREBP
CaTFSNP34	acgttggatgTTTGCAAAAGATGCAGTTCA	acgttggatgAAGCCTAATCAAGCACTATCAGC	CCATTAAAAAAGCAGTGTGCTGA	G	A	188	58.05	CaChr1	24068638	ARF
CaTFSNP35	acgttggatgTGGCTAAAACTGTTGCCTTC	acgttggatgGAACCCCACAAATGGATCAC	GGGGAAAATGGATCACAAGAAGTC	T	C	86	45.3	CaChr1	25186105	Tify
CaTFSNP36	acgttggatgCCGTTTGAATGCATATTGGA	acgttggatgGAGGAGGAAGACACATCCTGA	GACACATCCTGATGCTGCT	A	C	120	59.38	CaChr1	26026317	SBP
CaTFSNP37	acgttggatgCTTCCAGATCTTCCAGAGTG	acgttggatgTAAGCGTAGCTGTCGCAAAC	TAGCGCCGAAGAAAGC	C	T	107	50.1	CaChr1	33623744	SBP
CaTFSNP38	acgttggatgAGAGGTGGTGAACCAAATTC	acgttggatgTGTTGGACCTCAAGGAATGC	CCGCCTGAAAATATGGCACT	G	T	83	48	CaChr1	37691837	G2-like
CaTFSNP39	acgttggatgCAATTTTTTGCTTATGGGTC	acgttggatgCTTGACCTTAACGTTCCTCC	CCTGAAGTTGCTTGATCTG	G	T	95	48.6	CaChr1	41981594	AP2-EREBP
CaTFSNP40	acgttggatgCAAACACATTTGGAGGAGCA	acgttggatgTGGGTTATTTCTTTCCATTTTTG	CCCTTTATCATGTGATAGAAAAGT	G	C	119	59.69	CaChr1	43957759	NAC
CaTFSNP41	acgttggatgGGCTTTTCCTCCTATTCTGC	acgttggatgCCATGAATCTGGTTCAAGGC	GAGGGCTCTAGTTTGAA	C	G	95	45.5	CaChr1	46718906	mTERF
CaTFSNP42	acgttggatgGAAACTTGCTTATTTTGCACCA	acgttggatgGAACCAATTGCATTTCGTACAG	TGTTTCAATACATAGCAACTAGATAT	T	C	98	59.3	CaChr1	48002347	PHD
CaTFSNP43	acgttggatgCGCAGCAGCTACCACATAAC	acgttggatgGAAGCGAAGAAATTGGAGCA	GACGAAAGAAATGTGGAGAAATC	T	C	184	59.52	CaChr1	48218880	Alfin-like
CaTFSNP44	acgttggatgATTCCTCGATTTCTCCTCGG	acgttggatgAATGATGTCTCGAGGAAGGC	GGGCGAGGAAGGCAATAGAGTCACT	C	A	101	54.3	CaChr1	48249846	bZIP
CaTFSNP45	acgttggatgTCTCTTCTTTCGACACGTGG	acgttggatgGCCAATAACTTCGGAAGCAG	GAGTCGGAAGCAGCTGCTAA	G	A	103	51.5	CaChr2	355965	LOB
CaTFSNP46	acgttggatgCCAAGGCCAAAAATTCTGTT	acgttggatgTCATGGAGCAATGGAAGTGA	GCATTGAAGTAGTTGTGTCATGCT	A	T	231	59.06	CaChr2	3202163	HB
CaTFSNP47	acgttggatgATTGACCCTTTTGCCCTTCC	acgttggatgCAAACCAACATTGACATGGC	CCATTGACATGGCAAAAAAAGCACTTAG	G	A	112	54.5	CaChr2	3353616	MYB
CaTFSNP48	acgttggatgCATGCAGCTAATGTTGGAGGT	acgttggatgCAACCACAAGCAGCACAAAT	CTAATGTTGGAGGTTATGCT	G	A	104	60.15	CaChr2	3410435	zf-HD
CaTFSNP49	acgttggatgTTTATGATGCGTGCAACCTT	acgttggatgGCTCTGTCCTGCAATCAGGT	AAAGTCATCTTGTATTCAGAACCCTC	A	C	199	59.19	CaChr2	4324332	SAP
CaTFSNP50	acgttggatgGAGGGGAAACAAACATGGAA	acgttggatgCCTTTTCCACTCCCCTTTTC	TGTTATTTCGTCCATTACGGTTG	A	T	199	59.77	CaChr2	5886240	PHD
CaTFSNP51	acgttggatgAGAGGAAAGGGTGGGGTTT	acgttggatgTCTCAAACATGCGTTTCCAG	AACATTACGACGAACTGGCAT	T	C	200	59.78	CaChr2	6181000	SET
CaTFSNP52	acgttggatgGGGTGTCCATTCAGATTACG	acgttggatgGGTTGTGAAACCCACAAACG	TCACCATTTCTTACGGG	C	T	102	46.3	CaChr2	6743024	FAR1
CaTFSNP53	acgttggatgGGCTTCAATTCTTCGAGCAG	acgttggatgTTTTCTTGAGGACCATCACTG	CAAAAACGTTGGTGTC	G	A	116	60.1	CaChr2	9152974	bHLH
CaTFSNP54	acgttggatgAGTAGGCGAAGGAAGAGAAG	acgttggatgGAAGAAATGCATTTGGCGGG	ATTTGGCGGGAACTGT	T	C	98	49.2	CaChr2	9266035	GNAT
CaTFSNP55	acgttggatgAACGCCTTTAACCGAATCCT	acgttggatgCTTCCAACACGGCATCCTAT	GTGAACCATGGCTGGAAAAG	T	C	203	59.97	CaChr2	10330226	bHLH
CaTFSNP56	acgttggatgCCATGGACATGGTCCATGAG	acgttggatgTTTCAATTCCTCTCGCTCCC	CCCCCCTTTACAAATCCACAAAA	G	A	85	46.8	CaChr2	14248647	GNAT
CaTFSNP57	acgttggatgGCAACCTCTCTTTTCTCACC	acgttggatgTGTAAACTCCCTGGAGAGTG	AGCGACTAGAGTATGATTTTTGGAAGT	G	A	107	51	CaChr2	15817338	C2C2-YABBY
CaTFSNP58	acgttggatgTTGGCTCCTCTTCCAGTTTG	acgttggatgTCGAATCTTCAATACAAAACATACC	CCTCACTTTGATTTTCAAGA	G	A	84	60.37	CaChr2	16132823	SBP
CaTFSNP59	acgttggatgCCGGCATTGTTAGACTCGTT	acgttggatgTTGTCATGGCCCCATAATTT	TGAGAAGAAAGTGAACAATGACAAAT	G	C	188	60.13	CaChr2	16353473	GeBP
CaTFSNP60	acgttggatgTCCTCTTCCCAACAAATCCC	acgttggatgCCTTCGTTTCAAGGGATTGG	GCTTGGGAAGGCTGTTGT	T	C	92	53.5	CaChr2	18418063	GNAT
CaTFSNP61	acgttggatgGATGCTGGACGAAATCATCA	acgttggatgTGAATCATCCGTGGTTGAGA	ATCCTAGTGCAGATGAAGGACC	T	C	225	59.61	CaChr2	18878024	bHLH
CaTFSNP62	acgttggatgCGGCGAATTTTGACCTTGAG	acgttggatgGAGATTCCAGAGGCATCAAC	AGGCATCAACTCCGGT	C	G	99	50.9	CaChr2	21714168	zf-HD
CaTFSNP63	acgttggatgTGCATAGCCACCGTCATCTA	acgttggatgAGGGTGAGGGTGGAGGTACT	TGCGAATCAACTTCCATATCAA	T	C	210	60.24	CaChr2	24236619	Alfin-like
CaTFSNP64	acgttggatgATTGGCCTCCTGTCTTCCTT	acgttggatgGGCATGCTGACGATGATAGA	TTCTCTTCTCTCCTATGTTGATCTTCT	G	C	202	60.07	CaChr2	25291561	C3H
CaTFSNP65	acgttggatgGAGGGAAGGGTTTGATGGTT	acgttggatgTCTCCAGGACCACAAAGGTC	GTGGTGGTGGAGTTGGTTC	T	C	207	60.17	CaChr2	26158345	FHA
CaTFSNP66	acgttggatgGCCTCCTGATAAGTTAGCTG	acgttggatgGCAGACTGTCGATTTGCAAG	TCTCATTCTCTTAGCACGTTTGGG	A	G	98	50.7	CaChr2	27361120	bZIP
CaTFSNP67	acgttggatgAACCAACCTTGTTCCTCTTTCA	acgttggatgCCAGCTTCAGCTTCTTGAGC	GCTTCTGTAAAGACATGGTAGAGAGA	G	C	194	60.01	CaChr2	27694185	AP2-EREBP
CaTFSNP68	acgttggatgTTGGAAATCACAGTGCATGA	acgttggatgGATGAAAGTTTCGGGTTTCG	CCTTTATTGAAGTCTCTAGAAGCC	G	A	89	58.62	CaChr2	30377757	MYB
CaTFSNP69	acgttggatgTAGCATCGGAACCTTCTTCC	acgttggatgTTTAGGAGATGCCCAACCAG	TCTTGAGAGTGAAGATGATGATGC	G	A	200	59.27	CaChr2	30698052	HMG
CaTFSNP70	acgttggatgAGTCCGTGTAGTTGAGATAG	acgttggatgGGAGCAAGGAATTCAACAGG	GGGGCTTTGAATCCAACAAACCTTGA	C	T	120	49	CaChr2	30867757	C3H
CaTFSNP71	acgttggatgTCAAATGTGTTTTGGGCAGA	acgttggatgCAGAGCCAACCAAAAGAAGC	TATCCCAGTGAATGGAGCAAA	G	A	203	60.09	CaChr2	32769483	FAR1
CaTFSNP72	acgttggatgAAAGTGTTGCTGCTCCTGGT	acgttggatgGCTGAAGCCTATGCACATCA	CATCAAACCTTAGCTAATATGTACC	G	C	117	59.91	CaChr2	32958106	bZIP
CaTFSNP73	acgttggatgCAAGTACGTGCACCAGCAGT	acgttggatgCCTCCACCTAAATGAAGCTCAG	AAAACATGTCCACCAATCTTCAC	A	T	197	59.97	CaChr2	33604180	SRS
CaTFSNP74	acgttggatgCCAGCTTTAGCGAGGATGAA	acgttggatgGCACAACAAACAGCAAATGG	CGAGGATGAAACTGCCGA	T	C	215	60.48	CaChr2	33661127	HB
CaTFSNP75	acgttggatgATAGTCCTTGCCACTTGACC	acgttggatgTTCAGATAGGATCAGCCAAG	CCGCCAAGATAATTCTGGCAATGTT	T	C	91	52.5	CaChr2	33727257	SNF2
CaTFSNP76	acgttggatgACGACGAAAGGTGGAGAAGA	acgttggatgTCCTGTGGCTTTCCAAAATC	TCCTATATTTTCTTCCTCTTAGGCA	T	C	202	59.84	CaChr2	34132454	NAC
CaTFSNP77	acgttggatgGAGGCTCGGACCATACTGAG	acgttggatgTTCCTCCTCACGATCTGACC	CATCTATGGTTGTGTTTCTCTCTCTACT	G	T	199	59.83	CaChr2	34853120	Bromodomain
CaTFSNP78	acgttggatgGGTCAAGGATTGCACCTCAT	acgttggatgCGAAACACCCTCAGCTTCTC	TGTGAAGTTCATTTACCAAGTACAA	G	A	209	59.93	CaChr2	35412866	mTERF
CaTFSNP79	acgttggatgCTCGTGAACAGGACCGATTT	acgttggatgATCGTCTCCGTTGATCGTCT	AAGATCTCGAAAGACGCGAA	G	A	197	60.11	CaChr2	35556870	CCAAT

SNP IDs*	1st Pre-PCR primer sequence	2nd Pre-PCR primer sequence	Unextended Primer sequence	SNP allele (ICC 4958)	SNP allele (ICC 17160)	Size (bp) of alleles amplified	Actual annealing temperature (0C)	Chromosomes	Physical positions (bp)	Transcription factors (TFs)
CaTFSNP80	acgttggatgTGCAGGCATCAGAACAACAT	acgttggatgTTGTAAACGGCGAAACTTCC	TTTACTGCACGTTGGAATCATC	G	A	214	60.27	CaChr2	36087743	GRAS
CaTFSNP81	acgttggatgACGAGGCACATAGCTCCAAG	acgttggatgCTAAGGGATCCCCAATTGCT	ATCAGAGATTGTTGCATTATTTCTTG	G	A	193	60.42	CaChr2	36337365	GNAT
CaTFSNP82	acgttggatgACCAGGAAGACTCAAACTCG	acgttggatgCACATTTTCCTCAAGTTTCC	GGCCTCAAGTTTCCTAATTTTGAAGC	G	T	111	50.9	CaChr2	36520110	FAR1
CaTFSNP83	acgttggatgGGCTTTCCTTGATGCATACC	acgttggatgTCTCCAATGGACAGAGAAGC	CTCCAATGGACAGAGAAGCAAGAGTC	C	T	116	57.3	CaChr3	2706867	C2C2-CO-like
CaTFSNP84	acgttggatgACCATTAGACCTGCGGTCAC	acgttggatgGGCATCGGCTTTACAAAGAA	TTTTGCCGCGCAGACT	T	C	213	60.00	CaChr3	2709320	C2C2-CO-like
CaTFSNP85	acgttggatgTGTCTTTTTCCAACGGTGTTC	acgttggatgGGCGCATTTTATGGAAGTTG	CACCACCTTAACAACCACCAC	A	C	197	60.00	CaChr3	3617757	zf-HD
CaTFSNP86#	acgttggatgGGCTACAGGTGGCGAAAGTA	acgttggatgGTGTTGTGGCTGCTGTGTCT	AACATGTCGAGAGAGCATCAAC	A	C	200	60.27	CaChr3	4823888	WRKY
CaTFSNP87	acgttggatgCGGAGTGAAGCTTCCTTATC	acgttggatgGATGCCTCCTGAGACTAATG	AAACTTAAGAGAGAAATGAAGATG	C	A	101	47.2	CaChr3	7436178	FHA
CaTFSNP88	acgttggatgAAAGAAGATACTGCGTGCTC	acgttggatgGACCTCGATCGAAAGAGATG	CCACTCTCCTCGGAC	A	G	100	47.4	CaChr3	10493006	PHD
CaTFSNP89	acgttggatgAGGCAATTTAGTCCCTGAGC	acgttggatgGCCGGAATTCTGCAATATGG	CTTTGCATTTGTTCAAGCCAAGATGTT	G	T	105	50.6	CaChr3	14026885	ARF
CaTFSNP90	acgttggatgCTACCGATGGCTCTGCAAAT	acgttggatgTCATGAGCTCCATTGTCTGC	AAGGAAGCTGAACTCTAAAGAAACA	G	A	202	60.24	CaChr3	15739519	FHA
CaTFSNP91	acgttggatgGCTTTGGCTCAGTCACCTGT	acgttggatgATTGCATACTAAACTCAAAGACCTC	ATCTAGATTGAAAGCAAAAAGGATTC	G	A	200	60.45	CaChr3	17174233	LUG
CaTFSNP92	acgttggatgTAGCGTTTAAGGAACCACCC	acgttggatgGCAATGCACAGTTACAGCG	CAGTTACAGCGAAGAGAT	C	T	83	46.5	CaChr3	17181273	LUG
CaTFSNP93	acgttggatgACCCGATAAACGACAGCAAG	acgttggatgCGAAGGTGGCCTAATAGGTG	TCTCTCTTTCACTAGTTTCTCGGC	T	C	200	60.13	CaChr3	17936789	MYB
CaTFSNP94	acgttggatgGGAGGGTTTGATGTCACCAT	acgttggatgCAACCAGGCTGAAAATAGCC	CATGAACAAATTTTCTTGGACT	G	A	98	59.64	CaChr3	20174631	S1Fa-like
CaTFSNP95	acgttggatgACCTAGGGTTTCCAAAACAC	acgttggatgTAAATCCCCCAATGCCATCC	GTAAACCCTTAGAGATCAATCATTCGAT	T	C	101	52.7	CaChr3	20843598	SAP
CaTFSNP96	acgttggatgGCAAAGGTTGGCACCTAAAG	acgttggatgCACCACTCCTTGATGGTCCT	TTGTTGAACAGCAACTATTATCAAG	G	A	227	59.75	CaChr3	21776235	GRAS
CaTFSNP97	acgttggatgCACGGTTGTCACTACGCAAA	acgttggatgTCAAATCCAGTGCCAGACAA	GGTTTGGGCATTGGCG	G	C	211	60.75	CaChr3	22065431	FHA
CaTFSNP98	acgttggatgGCTGGGAGAGGCCTTAATCT	acgttggatgCACAGCCTGCTCCTCATTTT	GCTGACACAGTTGTCATCTA	T	C	87	59.81	CaChr3	23278789	SNF2
CaTFSNP99	acgttggatgAAGGGTCACCATATCCAAGG	acgttggatgGGATCTGATCTGTTTCTTTC	TGATCTGTTTCTTTCAACTTGTTTTCT	C	A	111	52.4	CaChr3	23622995	WRKY
CaTFSNP100	acgttggatgCGGCGTCTGTTAGGTTCTTC	acgttggatgTGATAATGAAAGCCTCCTTGG	TCGGAAGGACTTGTTGAGTC	G	A	102	59.88	CaChr3	23898026	C3H
CaTFSNP101	acgttggatgGATTAATGCGTGTGGGAGGT	acgttggatgAATGCAAATGTCTTGGCACA	TGGGAGGTTGCCCGC	G	A	176	59.82	CaChr3	24542187	mTERF
CaTFSNP102	acgttggatgCTCCTTCACATGGCTTGATG	acgttggatgCCTTATCGGTTCCGAAATCA	AGAATATAATCACCAAGTGTTGAG	G	C	119	59.24	CaChr3	25508396	C2C2-CO-like
CaTFSNP103	acgttggatgTTGCTCCGAAACAACCTCTT	acgttggatgAATCGAAGGATACGCCAATG	CCGGATTTTTCTTTCTCCTCTAC	G	A	209	59.85	CaChr3	25757540	SET
CaTFSNP104	acgttggatgCAGGTCTTAGCAATGACACG	acgttggatgGCCCTATTTCTTCCCATGTC	CCATGTCTTGTTTGGATAC	C	T	81	46.1	CaChr3	26544003	NAC
CaTFSNP105	acgttggatgGAGGAATATGTCTTCTTCCATTCC	acgttggatgTGGAAAGAGTAATTTTCCCCCTA	CTCATGACTGAGTCACTTCCA	G	C	116	59.37	CaChr3	26593875	C2C2-GATA
CaTFSNP106	acgttggatgATGGTTTCCTTCCCCTTGAC	acgttggatgAATGGGAGAGGCTAAGAGTGC	TTAACTCATCTAAACAGTCAGAAATATGAA	G	C	188	60.17	CaChr3	28560960	SAP
CaTFSNP107	acgttggatgGAACTGGGGAGAGTCCAACA	acgttggatgGCACGTATGCCTTTTTCCTC	CAACAGATGATACAGAAGACAAAAATC	G	A	197	60.09	CaChr3	28615172	bZIP
CaTFSNP108	acgttggatgCCACATGAAGCAACGTTCAG	acgttggatgTGCAATGTTTGACGTGGAAT	GGTGCTCCTCTAGCCACCTT	G	C	235	60.3	CaChr3	29843867	bZIP
CaTFSNP109	acgttggatgGAGAAGGGTGATGGAGGTCA	acgttggatgCTCCCCATTCCCTTCTTTTC	GGGATGAGATTGAGGATCTTAGTAG	T	C	194	60.05	CaChr3	30320095	BSD
CaTFSNP110	acgttggatgGGCATGCAAATTTGAGCTTC	acgttggatgGAATTCCGACGTCGCTAAAG	GTAGCGTCTTCCTTAACCACAAT	G	C	197	60.73	CaChr3	30955375	GNAT
CaTFSNP111	acgttggatgCAAATTCACGAGGATGCTGA	acgttggatgGGGTGAACCCAAAGTATGGT	GGTATAGTGTCCCAGGAAGATTTG	G	T	197	59.8	CaChr3	31359638	Jumonji
CaTFSNP112	acgttggatgATGGCTACGCCTCAAATCAT	acgttggatgTTGGAGATGCTCTAACCTTCG	CAGGACAGTATTTTACCAACGCT	A	C	213	59.56	CaChr3	31566643	G2-like
CaTFSNP113	acgttggatgCACAGTTTCTCAGCGTCAGC	acgttggatgACCCTTGTGATCCAGCATTC	GGCCGATGACTTTGAGTT	T	C	99	59.78	CaChr3	32898333	S1Fa-like
CaTFSNP114	acgttggatgGAAATCTCGTTTTGTGTTGTC	acgttggatgACGTGGCTATGCTTTCTGTC	CGTCTGTCCGTAACACACATGA	G	T	93	51.6	CaChr3	32900447	S1Fa-like
CaTFSNP115	acgttggatgTGTGAGGTCAACACGGCTTA	acgttggatgCAAAAGTCGCTTTGTTTCAGG	AAAGAAGGGCTTGAGGGAAA	T	C	202	60.3	CaChr3	33155717	Sigma70-like
CaTFSNP116	acgttggatgTGAAGCATATTTGTCCCTTGG	acgttggatgGGGAAGGGCTACTTCCAGAC	CTGACTGCAGTTCTTTCTGTAGTTG	G	C	209	59.95	CaChr3	34594243	Alfin-like
CaTFSNP117	acgttggatgGGCCTGTCAAAATCCCTTCT	acgttggatgAAATGTCTGCAATCGTGGTG	CGTCCCTCATAGTTACGACTCCT	T	C	201	60.44	CaChr3	34848851	C3H
CaTFSNP118	acgttggatgTTGATGGAACCTGGGTTGAG	acgttggatgAGCATCAAAACCTCTGCACC	CTCTGCACCATCACA	G	A	100	45.2	CaChr3	35935079	Jumonji
CaTFSNP119	acgttggatgTTCCTCATCCTCGATCGTCT	acgttggatgTTCCGCTTGATTTCCTTTTG	ATCTCCTCTTATTATTTCGATTTTATTTG	G	A	202	59.76	CaChr3	36809912	bZIP
CaTFSNP120	acgttggatgCTTCCACCCAACCGTACTTC	acgttggatgTGTTCCCCAAAGATCAGGAA	GTTCCCCAAAGATCAGGAAAG	A	T	204	59.45	CaChr3	37102025	SBP
CaTFSNP121	acgttggatgTCAACAACGGCGATAAACAA	acgttggatgCCTCAACCATCCCAATCCTA	TCAATCAATCCAGATACGTTTTTC	T	C	218	60.11	CaChr3	38614598	GeBP
CaTFSNP122	acgttggatgTGCTCTTCTCAAAGCCAAGC	acgttggatgTTATTTCCACAAGGGCCAGA	CTACAGCGGCGCAAGC	T	C	168	60.8	CaChr3	38760068	bZIP
CaTFSNP123	acgttggatgAACTGGAGCAGCGGTAAAGA	acgttggatgCATTTGCAGCCTCTCCTTTC	TTCTTCCATTTTCAGTGATTTCG	G	T	181	60.02	CaChr3	38914646	mTERF
CaTFSNP124	acgttggatgTAAGGACCCACTTTCCATGC	acgttggatgGGAGAGACTGCATCATCAAGG	GGATATCTGGGTTGACCAATAA	G	C	90	59.93	CaChr3	38969598	MYB
CaTFSNP125	acgttggatgTTCTCCAGCAATGACGACTG	acgttggatgGCCTGCTTCCTTAGTCTGGAT	CAAATGTCACAAGTGCCCC	G	A	198	59.98	CaChr3	39312173	bZIP
CaTFSNP126	acgttggatgGGGAACCGAAGAAGAAATCC	acgttggatgTTTTCGGCCTTTCAGACAAT	GGTGCCGGAGATGGC	G	A	104	59.88	CaChr3	39546588	AP2-EREBP
CaTFSNP127	acgttggatgCCTGTTTTCCTTGGCCTCTA	acgttggatgTGAGGAGGAGGGACTGCTAA	TAAGGTTTAAACCCTGTTCG	G	A	109	59.31	CaChr3	39788772	MYB-related
CaTFSNP128	acgttggatgCCAATACCAATTAGGCATCCA	acgttggatgAGCCGTGGGGGAATAGAATA	CCGGGTAAAATATCTTCCTCTTC	T	C	233	59.67	CaChr3	39816472	ABI3VP1
CaTFSNP129	acgttggatgACATAGATTGGAGGCCAAGG	acgttggatgTAGTTTGGAGGGAGAAGTGG	GGATGATGATAGTGAGAGTG	T	A	99	47.1	CaChr3	39817437	ABI3VP1
CaTFSNP130	acgttggatgGCGGTATGCTTAACTGTTCC	acgttggatgATGATGCAGAGGCTTCATTC	AGTCTTCTTCTTCTTCTTCTTTT	G	T	111	45.1	CaChr4	19353	bZIP
CaTFSNP131	acgttggatgTGAATCGCATGATACCACCA	acgttggatgAGGACAGGTTGTTCCCATGA	TGATACCACCATGCTCTTTGG	A	C	197	60.9	CaChr4	832308	AP2-EREBP
CaTFSNP132	acgttggatgAGCATACAGGTGTGCTGTCG	acgttggatgACATGCTCGGTTTTCTTTGG	GCGTGATTTAATTATGCC	G	T	105	59.93	CaChr4	1047604	SNF2
CaTFSNP133	acgttggatgCAAATCCGGAACTTGCCTTA	acgttggatgCATTTGTTGTTGCTGCTGCT	AATTCCAACGACGAGTTTCC	T	C	188	60.07	CaChr4	1494086	WRKY
CaTFSNP134	acgttggatgTCCAGCAGAAAATGATGGTG	acgttggatgTTGCACACAAGAGTTGAGAACA	GAGAGCACATTACTTCTCACTCC	T	C	204	59.65	CaChr4	1619541	bHLH
CaTFSNP135	acgttggatgTCCAAACACTGCTTCTGGTG	acgttggatgTGGCTTGGTTTGACTGAACA	TGGCCCTGCACAGTACA	T	C	95	59.87	CaChr4	2005078	PHD
CaTFSNP136	acgttggatgGGGTGGTGACCCTTTCTGTA	acgttggatgCTTTATCGGTTGGCCAATGT	GGATTTTAGTAAACAAGAAAATAAAGCAG	G	T	201	59.82	CaChr4	2168233	SAP
CaTFSNP137	acgttggatgTTCACAAAACCCAACCACCC	acgttggatgTGTCAAGAAGAGGTTAGGCG	GAAGAGGTTAGGCGTATGAA	G	A	94	49.5	CaChr4	4276290	Jumonji
CaTFSNP138	acgttggatgTCCATTGTCCAAACATTCCA	acgttggatgCCCTGTAAAGGCGTCTTCTG	GAACTTCCCTTTCTGATTTTGACTA	G	A	198	59.75	CaChr4	4745377	G2-like
CaTFSNP139	acgttggatgCTGGTTGGCTCCACCTTCTA	acgttggatgGGAATTAGTCCCCAGCATCA	CTGGTTGGCTCCACCTTCTA	T	C	199	60.25	CaChr4	5004108	bHLH
CaTFSNP140	acgttggatgTGTTTGGATCGAGGGTCAAT	acgttggatgACGAAATTCCCGACCACTTA	GCTACCTCTCCCCTTTCTTCTTC	G	T	202	60.32	CaChr4	5022053	bZIP
CaTFSNP141	acgttggatgCCAAGGGAAGCTTGTTCTACA	acgttggatgCCTTTGTTTTCATCTGCTGGA	GCTTGTTCTACAATCTGATCAAAA	G	C	108	59.36	CaChr4	5080383	Trihelix
CaTFSNP142	acgttggatgGAGGACAACTGCTGCGAATC	acgttggatgCTTCCTGGTGTGTTGTTGGA	AATTGAACGACTGCCAAATC	T	C	203	60.96	CaChr4	5677430	MYB-related
CaTFSNP143	acgttggatgAATTCCGCCATGAGAGCTAA	acgttggatgCAGGAGAATCGTCACCATCA	GCACGTCATGCTTCCAATC	A	T	208	59.81	CaChr4	5747340	Jumonji
CaTFSNP144	acgttggatgTCTTGGAAAAGAAACCCAACA	acgttggatgAGGTTCCACTTCCATCATGC	AGTTGAATATCAATAAGAAAGAGTTT	G	A	95	59.57	CaChr4	6183406	HB
CaTFSNP145	acgttggatgGGCAAATTTCCCACTTCAGA	acgttggatgTCAAAAGTTTTTCCTGTTTGGTT	TGGTTAAACCAATGACTAGA	T	C	105	60.05	CaChr4	6766479	MYB
CaTFSNP146	acgttggatgTGCCCCAAATTGTCGTATAA	acgttggatgCACTGCAGCTCCTTCTGATG	CCACTTTGAAAATCTCCCAA	T	C	116	58.89	CaChr4	7394399	AP2-EREBP
CaTFSNP147	acgttggatgGCCGTTCGTAGAAGCAGAAC	acgttggatgCCCAGTTGGGTCGTTACTGT	AAGCTCTGAAGATGTTGGAACC	G	C	196	60.02	CaChr4	7974685	C3H
CaTFSNP148	acgttggatgGAGTAGAATGCTCTCTCACG	acgttggatgTTCCGTTAACGATTCAATCC	TCAATCCAAAACTGCTTCT	T	C	107	47.6	CaChr4	8390599	BSD
CaTFSNP149	acgttggatgCCTCAGAATTGAAGTCAACAACA	acgttggatgGAGCCAACACCTGTTAGAGGA	GATAATATATCAGACTCATTTTTCACCAAT	G	C	191	59.27	CaChr4	8471278	AP2-EREBP
CaTFSNP150	acgttggatgTCCCTTTTGCTCCCAAAAAT	acgttggatgTCGCTGAGGAGAGAGAGAATG	AAATCTCAATTCAAAAACCCTAAT	A	T	82	60.77	CaChr4	8769821	NAC
CaTFSNP151	acgttggatgGGGGAGCACAGATTTACGAG	acgttggatgACGATTTCGGATTTGGAATG	GGCGCAGGTAATGATGC	G	A	208	59.69	CaChr4	9471744	AP2-EREBP
CaTFSNP152	acgttggatgTTGCAAGAGGTGAAGTGTGG	acgttggatgGGGGGCAGAAGAGTGATTTT	GAAGTTGAGACACGACTCCATCTA	A	C	177	59.87	CaChr4	10704805	mTERF
CaTFSNP153	acgttggatgCTCGTGCTCACAAGTGCTTC	acgttggatgGTGCCAAGGATGGATCTGTT	AAATGATCATATGGGATTTAGAGCA	G	A	197	59.78	CaChr4	10973664	SAP
CaTFSNP154	acgttggatgGAAAAGTTTGGGCGTTCTCA	acgttggatgATAATCGGAATCGGCGAAC	GGGCGTTCTCATTTGG	G	T	92	60.23	CaChr4	11054722	NAC
CaTFSNP155	acgttggatgTCGACGAGTGAGGATGATGA	acgttggatgCGCGAATCTCTTTCTTCCAC	CCTCGCCTATATAAGAAACCGC	G	A	213	60.36	CaChr4	11600593	Trihelix
CaTFSNP156	acgttggatgCTTGTGGCATTGTGGAATTG	acgttggatgTCATAGGCGCTTCCAAGATT	GCTTCCAAGATTCGACGTAA	G	A	220	59.96	CaChr4	12315577	RWP-RK
CaTFSNP157	acgttggatgCCAGAATGGTCTCCCAATGT	acgttggatgGCCTCAAGAGCTTTGCAGAT	CAAAGCCAAGGTTCCACG	G	T	209	59.78	CaChr4	12318493	RWP-RK
CaTFSNP158	acgttggatgCATCATTAGAGGAACATGG	acgttggatgAGGACATGTGATCCAAAGGC	TTCCAAATCCAAATCAAAATCCATA	G	C	101	50.1	CaChr4	12319718	RWP-RK
CaTFSNP159	acgttggatgTTGGGAGAAGAACTCCATGC	acgttggatgTTTTGCACTTGTTGCTTTCG	TTAGCATGAAAGATTTGCAGA	G	A	101	60.2	CaChr4	12964701	MADS
CaTFSNP160	acgttggatgACAGCCTAGACTGCCTCCAG	acgttggatgCTAGGCCCAATGTTTCTTGC	TTCTTGTCAGTCATACAGCATCG	G	A	190	59.62	CaChr4	13328322	Tify

SNP IDs*	1st Pre-PCR primer sequence	2nd Pre-PCR primer sequence	Unextended Primer sequence	SNP allele (ICC 4958)	SNP allele (ICC 17160)	Size (bp) of alleles amplified	Actual annealing temperature (0C)	Chromosomes	Physical positions (bp)	Transcription factors (TFs)
CaTFSNP161	acgttggatgCTCCTTCAACAAGGGCAGAG	acgttggatgTATGGGAACAATGCATGCTG	AACACAGTAGGGTCAAGATGAAAAG	T	C	196	59.98	CaChr4	13727396	Alfin-like
CaTFSNP162	acgttggatgGGCATATTCGCGTAAACGAT	acgttggatgTGGATCTTGGCCTTTACGTT	TGAGAAGTAGATAGGGCTCAAAGAAA	A	T	221	59.96	CaChr4	14094264	FHA
CaTFSNP163	acgttggatgCCACGAACACGTTTGCTAGT	acgttggatgCCGGAATACCCTCAAGCTAA	TTGGGTGTTCCGTCGAA	G	A	191	58.85	CaChr4	14511914	LIM
CaTFSNP164	acgttggatgACCATCCTGCAGACCTCAGT	acgttggatgAGGAGGGCAGAAAGAGGAAG	TGGTCTCCATAAGGGCATGT	A	T	195	59.71	CaChr4	15539515	C3H
CaTFSNP165	acgttggatgGACTTGTACGACGCCGTTTT	acgttggatgTGATGACAAAGAACGTGGCTA	AAAACCGCTAGAGCTTGTGA	G	A	200	60.18	CaChr4	16279070	C2C2-CO-like
CaTFSNP166	acgttggatgCCAAAAAGGACTTGTTCTGG	acgttggatgAAGTCAAGTCAGATCCACTC	TCCACTCTTAAGAACAAAAA	G	C	119	45.1	CaChr4	16362489	AP2-EREBP
CaTFSNP167	acgttggatgCTTCCACCATTGACATGCAC	acgttggatgAAATGGAGGCGATTCATCTG	TCGTTTCTTTCCCTTAGTAGCG	T	C	206	59.97	CaChr4	17427472	Jumonji
CaTFSNP168	acgttggatgTTTTGGTGAAGACCATGCTG	acgttggatgCCCTCACCAGAGCAACAAAT	TCAGATCATCAACTGTAAGCCG	G	T	203	59.69	CaChr4	17722046	SNF2
CaTFSNP169	acgttggatgGGTGCTCCATTTTGCAGTTT	acgttggatgCGGTTCAGCTCCAGATTCTC	GGTTCCAAAATAGGCGTGCT	G	T	181	60.12	CaChr4	17862210	HB
CaTFSNP170	acgttggatgATGGGACAAATGCAAAGCAG	acgttggatgAATTTTTGCAGTGGCTGAGG	GCGAATTGAGCCCTCAC	A	C	102	61.03	CaChr4	19013085	C3H
CaTFSNP171	acgttggatgTGCAGGAGTTGATGTCAAGAA	acgttggatgTTCCAATGCCTCATTCTTCC	AGAAGAAAAACACACTCCACCAA	A	C	198	59.43	CaChr4	19586476	MYB-related
CaTFSNP172	acgttggatgAAGTCTGCGTATGCATCGAG	acgttggatgAAAGGGTCCGTCAGTTGGAG	CTAAAAGCTCAAAAAGTCTCTTGTC	A	T	105	49	CaChr4	20446814	Sigma70-like
CaTFSNP173	acgttggatgCAAAGACAGTCCCTGAGTAG	acgttggatgGCAGTACAACATGTGGAACG	GAACAATTCAGCGGCGCGTGA	A	G	99	56	CaChr4	23557644	HB
CaTFSNP174	acgttggatgCAAACCCACATCAAATCTTG	acgttggatgCTTCTGAGGTTTTTCCTTGTG	CAAATTACTTGGTAGTGGATCTT	C	T	100	47.6	CaChr4	29778149	HB
CaTFSNP175	acgttggatgGCGTTGAACAAAATCTTGCC	acgttggatgAACTGTTCCTGATGGTACCG	CAACAGAATTTGGTTCGT	G	A	114	45.9	CaChr4	36535887	ABI3VP1
CaTFSNP176	acgttggatgAGAGGTCAGCGAAGGAAACA	acgttggatgGGCCAATTTGTTGCAATTCT	TTGAGTTAAAATATTGAACCAAAGAAAAC	G	A	206	59.99	CaChr4	36974817	SNF2
CaTFSNP177	acgttggatgGAGAATGTTTGTGTGAGGCTCTT	acgttggatgTGAAAAACAAACTGCGGAAA	AAGATACTCAAAACACAAAGGATC	T	C	101	59.81	CaChr4	37061273	SET
CaTFSNP178	acgttggatgTGTCTTGTGGATCACACATATAAAGA	acgttggatgGGCAGGAAGTAGCTGCGATA	CATATAAAGAAGTCCTTCTCACAA	T	C	112	59.83	CaChr4	37096147	ABI3VP1
CaTFSNP179	acgttggatgGGGAGTGGTGATTTCCTTTTC	acgttggatgCATGGAATGTGGACTTGAAGC	CTAGAGAGAAAATAACAACTTGGAATGA	A	T	185	59.8	CaChr4	37097065	ABI3VP1
CaTFSNP180	acgttggatgTTTCACAAATTCAAGCCAACC	acgttggatgTGGAATAAATTCCGTCATCACA	ATAGGGAGTGGTGATTTCCTTTT	A	T	189	59.97	CaChr4	37097068	ABI3VP1
CaTFSNP181	acgttggatgTGTTATCCTTGCTTCGCTGA	acgttggatgCAACCACCATGTTTCCATCA	CTCGCAAAGCATCACTGG	G	A	205	59.57	CaChr4	37514472	Tify
CaTFSNP182	acgttggatgTATCCGGGAGAAACAGATGG	acgttggatgGCTGCATAGGTTGAACACGA	ATTTGAGAATTGAAATCCCTTCC	A	T	203	59.89	CaChr4	38486601	MADS
CaTFSNP183	acgttggatgGCGTTGATGATCCTGAGTCC	acgttggatgGTGATGAAGCTGGAATGCAA	GCTAGCTTTTCAAGTCTTTGTCCTAT	G	C	203	60.63	CaChr4	38659802	GRAS
CaTFSNP184	acgttggatgTGGCGATCAAACTGTTGCTA	acgttggatgTAAAGTCAAGCATGCGTCGT	TAGGGCTGTTATTTCCCTGG	G	A	203	60.4	CaChr4	39804794	Trihelix
CaTFSNP185	acgttggatgCCGAGCGATTTGACAGTACA	acgttggatgTTCATGATCGTCCGGGTATT	AAGTCAGATACAAAAAGCACGAAA	G	A	200	59.86	CaChr4	40230993	Sigma70-like
CaTFSNP186	acgttggatgGGTGGTTTTGGTCAAGGATG	acgttggatgACCAGCAACCAAACCTTCAG	GGTGTTGTTTCTGGAAATAGGGT	A	T	201	60.21	CaChr4	40578365	Jumonji
CaTFSNP187	acgttggatgAACGGTTTCACGTCGTTGTC	acgttggatgGAACTTTTCACGAAGCGTCC	CCCAATTGCCGGAGGA	T	A	100	48.8	CaChr4	40933076	bZIP
CaTFSNP188	acgttggatgAAGTTTCTCTGCAAAAACTCGTG	acgttggatgTGGTGACAGAAACAAAATTGATG	TTCTCTGCAAAAACTCGTGT	G	C	111	59.98	CaChr4	41866592	LUG
CaTFSNP189	acgttggatgTTGCTTAGAGGACGCTGTTG	acgttggatgTGATGATTGAGCCCTTTCGT	GACGCTGTTGGCTTGAC	T	C	91	59.22	CaChr4	42354903	bZIP
CaTFSNP190	acgttggatgTGGTTATGGTAAGCCATT	acgttggatgACTCACCCGATCCTAACT	AGATTCTAATTCTAATT	G	A	91	51.25	CaChr4	43891050	HB
CaTFSNP191	acgttggatgTTCCTTCCTGTTGAATGA	acgttggatgTTCATGGCTTAATGATATTG	ACTGTTTCAATATTATATATAT	G	C	97	38.89	CaChr4	44393302	C2C2-CO-like
CaTFSNP192	acgttggatgGGAATCGCATAGCTTGGTAG	acgttggatgCCCTCCCAATCCAATTCAAC	ACACTCTCAACCTTCAAAG	C	T	118	47.5	CaChr4	44394420	C2C2-CO-like
CaTFSNP193	acgttggatgGGGTCCATTGCAAAGCATA	acgttggatgCGGCTAGGCAAGAGTTTGAA	CAAAGCATATTTAGCTGATTTTCAAT	T	C	203	59.49	CaChr4	45416533	HB
CaTFSNP194	acgttggatgGACAGAGACCATGGGGAAAA	acgttggatgAGTATTCGGCGGAAAAGTGG	GCAGCAGAGATTAGAGATCCACA	T	C	203	59.9	CaChr4	45736499	AP2-EREBP
CaTFSNP195	acgttggatgAGGTGACCATGAGAAAAATGC	acgttggatgTCCACACACTTGCAAACAAA	GCAAGTTTCATATTTGTGAACTACA	T	C	194	59.04	CaChr4	45835578	LIM
CaTFSNP196	acgttggatgTACGCCCTCTTGCTCCTAAA	acgttggatgGGAAGCACTTTCGGGTCTAA	CACTTTCGGGTCTAATGAAA	G	C	107	59.97	CaChr4	46419887	HB
CaTFSNP197	acgttggatgTGTGAAGCGTATAAGCGGTCT	acgttggatgAATCCCCCAATTCACTACCC	CAGGGGAGAACACTTTACGG	T	C	198	59.92	CaChr4	46995447	CCAAT
CaTFSNP198	acgttggatgTTAAGCGGAGAGGAAGACCA	acgttggatgCAAGCAAACATTCGTTGGTG	AAAATGAGATAGAAAATACCCTTCTGTT	A	T	201	59.95	CaChr4	47258388	ABI3VP1
CaTFSNP199	acgttggatgACCGATTCAACCAATTCAGC	acgttggatgTTCCAGACGTGGAAAACTCA	CAATTCAGCCGATTCAACC	G	A	203	59.94	CaChr4	47445593	LIM
CaTFSNP200	acgttggatgATCCTGATCACGGAAACCAG	acgttggatgGGCGTAACATGGATGAAGGT	CAACCCATCTTTGATCTCAAC	G	A	96	59.93	CaChr4	47884183	HMG
CaTFSNP201	acgttggatgGGCTTGGTTCGGATACATTG	acgttggatgGGAGATGTCAAGCTGATTCCA	GTAGGCAAGATTCGCAATTC	G	A	101	60.33	CaChr4	48124866	HB
CaTFSNP202	acgttggatgGATTTGTTGCTTGGTGACGA	acgttggatgGAGCCACAGTTCAACCCACT	GCACCACGGGATTTCCT	G	T	185	59.7	CaChr4	48276063	SWI/SNF-SWI3
CaTFSNP203	acgttggatgGGCTGACCTCGGTATATATG	acgttggatgTCAACCTCCAACTCAAGAAC	TGTTCGAGATTTGCATGATAA	C	T	101	48.3	CaChr4	48384848	ARF
CaTFSNP204	acgttggatgCCTCCTGCAGCAGAATCTTG	acgttggatgTGATCCTAATTACCGTGCAG	TGGCAAGGTTAATGATGATGCAGCAACA	A	G	110	54	CaChr5	166360	NAC
CaTFSNP205	acgttggatgACGACAGTGTTTCGGAATGG	acgttggatgCATGATCAAGCCCTATAGAC	ATTGGTGACATAGGTATCACTT	G	A	98	49.5	CaChr5	7382449	bHLH
CaTFSNP206	acgttggatgTGTTGAAAAATGGGGAGAGG	acgttggatgCCATTTCTTCTCTTGCAGAAAGTT	GAAAAATTGAAATAAAAAGGATTGA	G	A	89	59.9	CaChr5	7681689	MADS
CaTFSNP207	acgttggatgGGAAGTGCCGTTGAATTGAT	acgttggatgTTCATTGGCACAACCATCTC	TTTTCTGGAACATTGACACTTGA	G	A	191	59.94	CaChr5	9958884	WRKY
CaTFSNP208	acgttggatgACTTACTGCCTGAGCGGGTA	acgttggatgATCCTTCAATCGACCACGAG	TGAATTTTCAGATTCTTGCTTGTT	T	C	187	59.9	CaChr5	10989680	FAR1
CaTFSNP209	acgttggatgGGAGGTTAATGAGTTTGCAAC	acgttggatgGAGAAGGAATTGGACTTCCG	GCGTTGGTGTTGCTG	G	A	104	48.7	CaChr5	11384336	C3H
CaTFSNP210	acgttggatgGACGGAATTTATTCCAACCA	acgttggatgTTGACCAAAACAGAACATGGA	TCATTTCTAGTGAGCTTCAAGTC	A	C	87	57.4	CaChr5	14755276	ABI3VP1
CaTFSNP211	acgttggatgTCCAGCTTACTTGAACTTGC	acgttggatgCATACCAGAAGCCAACCTCA	CCCTCATTTATCAGAGAAGCG	G	A	120	48.3	CaChr5	16677409	C3H
CaTFSNP212	acgttggatgTTGGTGGTTGAATAATGTTGTCA	acgttggatgACATGAACCGCAAACAAGTG	GTTGTCACTTGTTGTAGGCTTA	T	C	116	60.14	CaChr5	19048521	GNAT
CaTFSNP213	acgttggatgACCTCATCACGAGGCTTTGA	acgttggatgAGGGATTCGGAGAAGGATGT	CCGCAGCATCACTCCAC	G	T	206	60.8	CaChr5	21801207	FHA
CaTFSNP214	acgttggatgTTCTCGTTCTCCTTCTCCAC	acgttggatgAATCCCTCCCTCTATGGTTC	ACTCTATGGTTCCTCTCGTGCTC	C	G	118	54.5	CaChr5	21801378	FHA
CaTFSNP215	acgttggatgCCCATCAAACAAAGAGAGTTGG	acgttggatgTCAGCTGATGATATGGATGGA	AGAGTTTGTTTGTTTCTCTCCA	A	T	102	60.87	CaChr5	24343946	CCAAT
CaTFSNP216	acgttggatgCAGCCAGCTATGACAGGATTC	acgttggatgTGCATATTTTGACCCTGCATT	TGACCCTGCATTTTAAACTC	T	C	110	59.85	CaChr5	24717637	MYB
CaTFSNP217	acgttggatgTGGATCATTCCCAATGAATC	acgttggatgTTCTGTAGGCTCCTCCTTAG	GAATATTAACACGTTGTTGATTC	A	G	111	45.4	CaChr5	26426790	WRKY
CaTFSNP218	acgttggatgAAATGCTGAGGAAGCGTGAT	acgttggatgGAATCACACCCACCCACTTC	GAGAAAATGTACTATGTTGGTGACG	G	A	202	59.84	CaChr5	27042239	MYB-related
CaTFSNP219	acgttggatgTCGGTTCAAAACCCTAATGC	acgttggatgTCTGTCCAGCACTTGACTCG	GTCAGATACCAACGGAGCATC	G	C	203	59.94	CaChr5	27150196	ABI3VP1
CaTFSNP220	acgttggatgAAGCTGCCAGCCTGATCTAA	acgttggatgTCTTCGTTTTTGCACAATGC	TCACTTGACATCTCTCGGTTCA	T	C	201	60.12	CaChr5	27749046	ARF
CaTFSNP221	acgttggatgGGCATGAAAGGCTATGAACC	acgttggatgTCTTGCACTCGTTTGAATGAA	ATGAAAGGCTATGAACCACG	T	C	118	59.53	CaChr5	28253736	LOB
CaTFSNP222	acgttggatgGCGAGAGGTACACAAAA	acgttggatgAATAACACAATTTGGAAGC	ACAAATCAATTTCTATTT	G	C	73	49.91	CaChr5	29397487	AP2-EREBP
CaTFSNP223	acgttggatgCCCTGCAGGTCCTGAAAGTA	acgttggatgCATGCTGCAATACTGGATGG	AGAGCATGCGAAGTACACCAC	T	C	189	60.25	CaChr5	29790311	FHA
CaTFSNP224	acgttggatgTGTGGCTCCTGATTCATGTC	acgttggatgGATCCATCAGCCACTTGTAG	TAACATGGTACCACCTGATTT	C	G	112	49.7	CaChr5	30439023	MYB-related
CaTFSNP225	acgttggatgGCTAGTGGCTCCAACGGTTA	acgttggatgGTAAGTGTCGCGCCTTGTCT	GATATGGAAAGAAGCAATTCACAGT	A	T	210	60.27	CaChr5	31244017	Bromodomain
CaTFSNP226	acgttggatgAGACGGAAGCATTCATGACC	acgttggatgAAACTGAAACTGGGCTTCCA	ACTCTGTTACCGGATGTGGTG	G	A	192	60.08	CaChr5	33879428	bZIP
CaTFSNP227	acgttggatgGACTGCATAGCATGTAACAC	acgttggatgATGAAGGTCCTCCGAAATGC	GAGAGGTCTAGAAGGCCTAA	G	A	100	50.4	CaChr5	34476592	Tify
CaTFSNP228	acgttggatgAGCAAAAGCCTTTCATGCAG	acgttggatgCAACATCACCCCCAGAGTTC	ACAGAATCTGGCATCACCATC	T	C	204	60.52	CaChr5	34510155	LUG
CaTFSNP229	acgttggatgGCAGATTCACCTCCCATCTC	acgttggatgAAATCTCCGTCTCGCAAATG	GTATCAGAAGTAGCACATGGAATCTC	A	T	201	59.62	CaChr5	34684944	bZIP
CaTFSNP230	acgttggatgTCGGACTTGAAATGGACCTT	acgttggatgGTCTCGGTTGCTGGTTTTGT	TCTCACAGGTTTCAGTAAATAATCACA	G	A	200	59.53	CaChr5	35117329	ARR-B
CaTFSNP231	acgttggatgCCACAGTAGAGCACGGTGAA	acgttggatgGTGCGTTCTCATTCAACTCG	GGTCCAAATCGCTGCCTAC	T	C	192	59.9	CaChr5	35122407	PHD
CaTFSNP232	acgttggatgAATCGGAAGACGTTCGTGAC	acgttggatgCTGCACCACCTGTGATTCC	CTGCGAAGTCTGCGAACA	A	T	199	60.12	CaChr5	35389971	C2C2-CO-like
CaTFSNP233	acgttggatgTCGTTCACCCAATCTTTTGC	acgttggatgCGTGTATGATGTATCATTACCAAAG	TAGCTAGGGCCTAGGGGG	A	T	105	61.01	CaChr5	36046998	HB
CaTFSNP234	acgttggatgCTCCCCCAACAATTTCTCAA	acgttggatgTGGAAATCAAACTCCGGTTC	AACAACAAGAACAAGTGGATGATG	T	C	199	59.9	CaChr5	36211837	BSD
CaTFSNP235	acgttggatgTGCAGCTGACTAAGACATGAAA	acgttggatgTTTGCCACAACAATAAATGACA	AAGCAGTAGAGCTAACATAAAATAT	A	T	120	58.73	CaChr5	36283170	LIM
CaTFSNP236	acgttggatgCAAGGAGATCGACGGTGAAC	acgttggatgCCGATGCAGTAGGTGGAAGT	CTGGATACCTCCACCACCAC	T	C	206	60.66	CaChr5	37147512	zf-HD
CaTFSNP237	acgttggatgGCCTTCCCACACTTTCTCTCT	acgttggatgGGTGAAACCCTAACCCTAACA	CTTCCCACACTTTCTCTCTCTAC	A	C	103	59.87	CaChr5	37485160	C2C2-GATA
CaTFSNP238	acgttggatgTCTCGTTGACCAATGCTGTT	acgttggatgAAGGAAAGAGAAAAGAGCCAAAA	TCTCGTTGACCAATGCTGTT	G	A	201	59.29	CaChr5	37814550	Jumonji
CaTFSNP239	acgttggatgTTTCGAAACATTTCGTGCAA	acgttggatgTCGAAACACGAAGAGTGCTG	CTAATCAAATCACCATAAACATCAGAATA	T	C	197	60.23	CaChr5	37985131	MYB-related
CaTFSNP240	acgttggatgTTTGGTCTGCATTTTCTGTCA	acgttggatgTTCACATGGATTCAGCAACAA	TCTGTCATTTTCGGTTCTTCCT	T	C	205	59.31	CaChr5	38149806	bHLH
CaTFSNP241	acgttggatgTGGGTGGAACTTTCATGTCA	acgttggatgAGAAAGCATTTGTCGCAGGT	ATGTAGAAGAGGCGGTGGC	G	A	203	59.94	CaChr5	38467232	GNAT

SNP IDs*	1st Pre-PCR primer sequence	2nd Pre-PCR primer sequence	Unextended Primer sequence	SNP allele (ICC 4958)	SNP allele (ICC 17160)	Size (bp) of alleles amplified	Actual annealing temperature (0C)	Chromosomes	Physical positions (bp)	Transcription factors (TFs)
CaTFSNP242	acgttggatgGCTTACGTGGATGCAGGTTT	acgttggatgTCTAAAGCTGATGGGCCAAA	CTAAAGGATCAAAATAAATGGGTTGT	T	C	202	60.14	CaChr5	40243059	FAR1
CaTFSNP243	acgttggatgTTTGGCTATGTTCCGTGTGT	acgttggatgTGCTTGTATCAAAGTGGCATT	CAGAAGGTGAATTAGACAATGATGA	T	C	186	59.05	CaChr5	40405468	FHA
CaTFSNP244	acgttggatgAAGAGCTTCATTCTCTTGCTGAA	acgttggatgAAATCGTCGTGCTCGTTTG	AGCTTCATTCTCTTGCTGAAG	A	C	118	59.8	CaChr5	40619677	HB
CaTFSNP245	acgttggatgTGAGCGCGAAATAGTATCCTC	acgttggatgCCCGAAATAGAGGAACAAGC	CTCCGAAACAACTCAACCG	T	C	200	59.47	CaChr5	41313407	SET
CaTFSNP246	acgttggatgAAAGGAGCTGTTCAAGCTCAA	acgttggatgATGCCTATGGGTCCAATTCA	TTTATTCGTCTTCTAATTATTTCTTGTATG	T	C	202	59.25	CaChr5	41478089	SWI/SNF-SWI3
CaTFSNP247	acgttggatgTTCGATTTGCAGAAGTGCTC	acgttggatgTGTTGGAAGGTGACTTCCAG	CCATTCTGTGCGGTTGTCCTTTATTGA	C	T	97	55.6	CaChr5	42527654	CAMTA
CaTFSNP248	acgttggatgTGCTTCACTTCCCCTAGACG	acgttggatgAGGATCTTGAGCCATGTTGG	GCGATTTTCCTAATGGTCTA	A	C	83	60.39	CaChr5	42984383	ARR-B
CaTFSNP249	acgttggatgAGGAAACCTTGCAGCAAATG	acgttggatgTGACCATACTCGTCCCATCA	CGAACCTGCTTAGTCTTGCAT	G	C	201	60.25	CaChr5	43835527	bZIP
CaTFSNP250	acgttggatgTGCATCTTCTTCACCAGAGG	acgttggatgACTTGCAAGGACAGCAGTGA	TTCTTCACCAGAGGCGTC	G	T	117	58.96	CaChr5	44421018	SET
CaTFSNP251	acgttggatgGCTAAACGACTCGCACCAAT	acgttggatgGGGGATCAATTGGTCTTCAG	TGCAGCAGAAAATCCTCCATA	A	T	196	60.28	CaChr5	44560496	HB
CaTFSNP252	acgttggatgGATGGAGTGCCTTCATTGGT	acgttggatgGTGAAGATGCAATTCCCACA	GAAGTTCATCAATCTTCTCAATTTTACTC	G	C	198	59.93	CaChr5	45076994	Sigma70-like
CaTFSNP253	acgttggatgAAGCTGCAGAAAGTCGTGCT	acgttggatgCAGAAGACCACAAGGTGCATT	AGTCTCTTAACCCCAAGATAGAACCT	A	C	210	60.34	CaChr5	46175167	NAC
CaTFSNP254	acgttggatgACACCGGGTTTCTCATGTTC	acgttggatgCGCATCAAAAGGCATTGTGG	GGTTGTTAACAGAAATGGAGGGACC	T	C	97	53.3	CaChr5	46513987	bZIP
CaTFSNP255	acgttggatgTTTGTTTTTGCGGGATGAAT	acgttggatgGCCTCATCAGCTTTTGGTTC	GTTTTGAAAAAGAAAAACCCACATA	G	A	175	60.3	CaChr5	47223799	C3H
CaTFSNP256	acgttggatgGTGCAGCAATTGTACAACCG	acgttggatgGTTGACCATTCACACATCGG	AAATTGGGTGGACAAGAATTTCCTACT	A	G	94	54.7	CaChr5	47734066	NAC
CaTFSNP257	acgttggatgTCACTTCCATGACATGAATC	acgttggatgGTAGGTTTCTTGGTGATCCC	TTTCTTTTAAATTTGGTGATGA	C	G	119	45	CaChr6	789313	GRAS
CaTFSNP258	acgttggatgTTGCTTTCCAAGCCAAGAAT	acgttggatgTGAAGGAGGAGCCATAAGGA	CATAACCACAAAATCTATAAGTTTCCATC	A	C	219	59.82	CaChr6	1609229	LIM
CaTFSNP259	acgttggatgCTTCGGATGCCAATTTGATT	acgttggatgGTCTGCTCCTCGACCAATTC	CATCTTCCCTCAAACTCATCC	G	A	205	59.9	CaChr6	1842423	GeBP
CaTFSNP260	acgttggatgTCAGCAGCAGAGCGTTTATG	acgttggatgAAGGGTTCAGCTGCTCAATG	GCCCTTCTACAACAAAGTCTCTAATC	G	A	188	60.3	CaChr6	2925948	Tify
CaTFSNP261	acgttggatgCAAGTCCAAAGCTGGCAAGT	acgttggatgTTCCCTGATAATGCCAATCA	CAAAGCTGGCAAGTTATAGATGAT	G	A	205	60.43	CaChr6	5506963	bHLH
CaTFSNP262	acgttggatgCCTGACTGGGATATCTTTGC	acgttggatgCTATTCATCGCTGGAAGGAG	CTGAAAGACTCACTCTGAGCA	T	C	116	47.3	CaChr6	5688576	LIM
CaTFSNP263	acgttggatgCCAACTTGGAATGGAGGAGA	acgttggatgAAGCTTCTGGAAGGTGTTGG	CCACAATCACAACCAACACC	G	A	184	60.04	CaChr6	5789222	SBP
CaTFSNP264	acgttggatgGGAGATTGGGTTTTGTGTCG	acgttggatgTGATGATCTGGTGCATTTGAA	GAACTTGAACATGAAGAGGACGTAG	T	C	203	60.35	CaChr6	6015415	NAC
CaTFSNP265	acgttggatgTTTTGGTAGCGTCCAGGAGT	acgttggatgTCCAAGACACGGTCCAAAAT	CAGAATATGGATGGCAGAATGA	G	A	190	59.73	CaChr6	6142063	ARF
CaTFSNP266	acgttggatgGAGCCCAAGATTGGTTGAAG	acgttggatgTAGAAGGGTTGGCAAGCAAT	CCTAAGTTGGATACCATACTCACATC	G	A	209	59.67	CaChr6	6295375	C3H
CaTFSNP267	acgttggatgGCATCTTGCGAGTCTGAGAA	acgttggatgGTTGGAAGGGATGGACTCAA	AACATTAACCTCCTCTGGCTTTAG	T	C	206	59.27	CaChr6	6913579	SNF2
CaTFSNP268	acgttggatgGTCCTTTCGCACCTATTTCG	acgttggatgTTCCATTTCCTCTGCGATTC	TTCCATTTCCTCTGCGATTC	A	T	210	59.71	CaChr6	7275013	MYB
CaTFSNP269	acgttggatgGGCCAGACACCAGAAACAAC	acgttggatgGCAAATCAAATCCAAACAACAA	AGGAAGAACGAGAGAAGAGTTTTTT	A	C	194	60.55	CaChr6	10032665	zf-HD
CaTFSNP270	acgttggatgTCATTTCTGAGTGAACTTGG	acgttggatgGAGCCTGATGGAAAAGTGTC	GTGTCTAGCATTACTGATG	C	T	118	45.2	CaChr6	10748091	mTERF
CaTFSNP271	acgttggatgTGCGATCTCTTCAATTCTGC	acgttggatgAAACAGGGAACCACTTGTCG	CATTAATATCGTATTCGTAACCAGATG	G	A	212	59.1	CaChr6	10864322	CAMTA
CaTFSNP272	acgttggatgGATGAGGGCGAAAATCAAGA	acgttggatgTGCAGAAAAATGCTGCTAAGTC	CCATCAGATCATATAGATAGCAAAAGAA	G	C	186	60.16	CaChr6	13242976	C3H
CaTFSNP273	acgttggatgCCGAGTTGCAAGAGAAAAGAG	acgttggatgCCATGTGATGAGACGTTGCT	CAGAAAATCAGAGTTGAAGAGTT	G	A	101	59.25	CaChr6	13996302	HMG
CaTFSNP274	acgttggatgCTGCAGATTTCTCCACGACA	acgttggatgAATGGTCCACCCATGGATAA	GTTCAGGGGGTTAAATCCAA	G	A	211	59.98	CaChr6	14815110	CAMTA
CaTFSNP275	acgttggatgTAAGCTCATTGAGCTCCGTC	acgttggatgCAACGATGTTGTCGCTAACC	TCACAATACGTGACCTT	C	T	100	45.4	CaChr6	15273685	Bromodomain
CaTFSNP276	acgttggatgGGAATTTGTGTCCCCTGAAA	acgttggatgTGCATGTAAGCTGACGTGTTT	ATCTACTTTTGCTCTATGGCCTTC	T	C	164	59.77	CaChr6	15848050	Jumonji
CaTFSNP277	acgttggatgCGATACAGAACCCACACGAC	acgttggatgGCATCCCAAGTGAAGCTCAT	AAGATCTTACTTCTGCTTTAAGTCTTTG	A	C	203	59.01	CaChr6	18443828	bZIP
CaTFSNP278	acgttggatgGGGAACTTGGATATGGCAGA	acgttggatgTGAGAGGGCTCATCATGACA	GAATTTTGGGGCATTTGGA	A	T	207	59.89	CaChr6	18949559	mTERF
CaTFSNP279	acgttggatgTCCACCGGTTGAGGTTTTAC	acgttggatgCCAATCTTATCTGATGGCTC	CCCCCATCTGATGGCTCAACCTT	C	T	109	48.8	CaChr6	20519235	NAC
CaTFSNP280	acgttggatgCTTCTCCGCCAGTTTCTGAC	acgttggatgAGAGATGGGGCTTGAAGGTC	CATCAGCAGGCTGAACC	A	T	101	59.99	CaChr6	21437289	Bromodomain
CaTFSNP281	acgttggatgACCCCAAAAGGGTCAAAAGA	acgttggatgGCGATTCTTTGCTTGAGAGC	TTGAGCCGATTGTCTATTTGC	G	A	205	60.69	CaChr6	21497337	bZIP
CaTFSNP282	acgttggatgTCGTCAATTGCAGATGTGGT	acgttggatgATATGCCATCATCCCATGCT	TGGAAGGTGTTGAGCAAATAGAA	G	A	211	60.12	CaChr6	21675697	HB
CaTFSNP283	acgttggatgGGGGTCCAATCCACCTTTAC	acgttggatgGGAGACGTGTTGCCAGATTT	CCTTGATGGTTCTTAGATTGATGATT	G	C	200	60.42	CaChr6	21716470	G2-like
CaTFSNP284	acgttggatgTGCATCCGGTTTTCTTTCTC	acgttggatgCCACCAAACCCGATGTTAAA	GAGTTTCTGAATCAGAGATTTTCT	T	C	109	60.19	CaChr6	21777225	AP2-EREBP
CaTFSNP285	acgttggatgACACACAATGTTGCGATTGG	acgttggatgTGGGAGGCCTAACTGAGATG	ATTGGAGGGGAAGGTTCTAG	T	C	100	60.44	CaChr6	22228859	E2F-DP
CaTFSNP286	acgttggatgTGAATGACTGCGCCAAATAG	acgttggatgTGGGTAACTCCTTTGGCAAC	ACTCTGACTCTCTGGCGACAA	G	A	205	59.83	CaChr6	22711105	ARF
CaTFSNP287	acgttggatgTGTCCCTTCCCATGTTGTTT	acgttggatgTGAAGCTCAGGCACATTGTC	TGTTGACACAGACAGAGAAACCAT	T	C	202	60.21	CaChr6	23284891	NAC
CaTFSNP288	acgttggatgTGATCGGGTCTATTGCCTTC	acgttggatgCTCTCAAACTCACAACCGTC	ACCTTCCCCATTAGGCGAC	A	G	116	52.7	CaChr6	24586525	bZIP
CaTFSNP289	acgttggatgTTGCAATTTGAGAGGGGTGT	acgttggatgATTCTTCCTCCCCCTCGAC	CTTCCTCCCCCTCGACTC	A	T	183	60.5	CaChr6	26357644	GRAS
CaTFSNP290	acgttggatgGGTGGCATGTTTCCTGAAGT	acgttggatgTCAGGATTCAACCCTCGAAC	GGAGAGAACGCTTGCCATC	G	T	199	59.97	CaChr6	27861705	C3H
CaTFSNP291	acgttggatgCTCGGCCATACAATTGAACA	acgttggatgATGGAGCATCATCTGGGAAC	TCTTACAGATATGTAGTGGAACTGATGA	G	A	199	59.54	CaChr6	28103045	HB
CaTFSNP292	acgttggatgTTCAGATCATCAAAATCGTGCT	acgttggatgTCTGTTTTGTGCCAAACGTC	ACAGAATCAATCAACAGCTAAATCAT	A	C	190	59.71	CaChr6	30942481	bZIP
CaTFSNP293	acgttggatgATGGCACCACTTTGGAAGTC	acgttggatgCCCATTGTGCTTCTCAGGAC	GGAAGTCCCTGATCCTGA	T	C	88	59.97	CaChr6	31477570	E2F-DP
CaTFSNP294	acgttggatgTAACCGGCGTTCCTCGATTG	acgttggatgGGGCTGGATGTTTCAAAACC	GGTAAAAACCAGGGGAGGC	G	T	113	48.8	CaChr6	31478512	E2F-DP
CaTFSNP295	acgttggatgCATCGGGTTCCTTCTGCTTA	acgttggatgTCCATTAGACTTTTCATGAGTTGC	CATTAGACTTTTCATGAGTTGCTTC	T	C	198	60.21	CaChr6	32082602	C2C2-YABBY
CaTFSNP296	acgttggatgGGCGATCCTAATTGATCACG	acgttggatgGGATCTTGGATTGGCTGCTA	GATGGAGAAGCCGGATC	G	A	84	60.44	CaChr6	32326867	WRKY
CaTFSNP297	acgttggatgTTCACCACCATCAAACAGCC	acgttggatgGGTGGTTTTTTTGGTGAATG	GGTGAATGAAGAGAGAAATTGTTG	G	T	101	50.6	CaChr6	37388155	OFP
CaTFSNP298	acgttggatgCCATTCCAAGGTCAAGGCTA	acgttggatgGCTGGTTGCCCAGTTAGAAA	GTACATCATGATTGTGTTTACCCTC	G	A	205	60.07	CaChr6	45709378	WRKY
CaTFSNP299	acgttggatgGAACCGGTTACTTATCAATGC	acgttggatgAGGTTCGATGTAGAACCTGC	GGTGGAGAACCTGCTAGAACCGTA	T	A	97	50.5	CaChr6	45713690	GRAS
CaTFSNP300	acgttggatgTCTCCAATGGAGGCAACATC	acgttggatgGCTGTTGAGTTGAGAGAAGC	AGAGAAGCTGAACAAGG	T	A	103	46.2	CaChr6	53173286	WRKY
CaTFSNP301	acgttggatgATTGGCAGCCATTGAGAAAT	acgttggatgAATGCTTCTTGGGTGAGTGG	AAAATTCGTGCCAAGCGCGG	T	C	94	59.53	CaChr6	53654154	bHLH
CaTFSNP302	acgttggatgTGGAAAATGGAAAACACCTTG	acgttggatgCCCAACCAACAGTACCAAGA	GCCTCATAATCAATTTAGAGACACTGA	G	A	213	59.82	CaChr6	54359260	GRAS
CaTFSNP303	acgttggatgGTTGGAATGAGCATGGGAAG	acgttggatgCCAATACCAAAACGTTGAGC	GCACTTAATGGAAATGGTTCAG	G	A	182	60.46	CaChr6	55333022	HB
CaTFSNP304	acgttggatgCAACATCTTCTCTTTGAAGC	acgttggatgAACACTCTGTTTTTGAAGGG	GAGGGGTAAAATCGAATAAGAAGGGT	C	A	99	45.9	CaChr6	55637657	ULT
CaTFSNP305	acgttggatgAAGCTTCTTCCAGCCTTTGG	acgttggatgCCATTGACCTGTTGAGCTTG	CTTCTTCCAGCCTTTGGA	T	C	117	60.87	CaChr6	57024461	MADS
CaTFSNP306	acgttggatgCCATTGTGGAGTTTGGGATT	acgttggatgTGGTACTGTGGGATCTGCTG	CAAGACCATGATAACACGACTCA	G	A	200	59.65	CaChr6	57532005	FAR1
CaTFSNP307	acgttggatgGCAAGGAAAAGAACCGTGAC	acgttggatgCTCCCATCCGAGATTTGAAG	CGATTAATGCAGCAAAAGAGTT	T	C	206	59.72	CaChr6	57720386	ABI3VP1
CaTFSNP308	acgttggatgGTCAAAACGATGGCAGGTG	acgttggatgCATGATGAATCCCCAAGGAC	AGACATTAATGGGTTTGGAAGTGT	G	A	212	60.1	CaChr6	58112367	MYB-related
CaTFSNP309	acgttggatgTTTGCCATGGAAGTGATTTTC	acgttggatgGGAAAAACCCAACTCTCTCCA	CACTAAGACTAAGAAGGATAATTTGTGC	A	T	211	59.93	CaChr6	58242882	mTERF
CaTFSNP310	acgttggatgTGTGTGATATTGAGGCATTGG	acgttggatgTTTTTACATGAAGAACTAGGTCCA	TTTTACATGAAGAACTAGGTCCAT	G	T	100	59.41	CaChr6	58555767	bZIP
CaTFSNP311	acgttggatgACAGCCACCGTAAGGTTTTG	acgttggatgCATTTGAACCCGGTTCAGAC	TCAGACCAGACGCAG	C	A	100	47.7	CaChr6	58810441	C2C2-GATA
CaTFSNP312	acgttggatgGCTTCTTCCCATTGCTAACG	acgttggatgTGAACTCTGAAACACACTCTTGC	CTTTAGCTTCCTTGGAAATCTT	T	C	110	59.85	CaChr7	82458	CCAAT
CaTFSNP313	acgttggatgTCTGGAGGACCAACACCAAT	acgttggatgCCGGTTGACTCGCTAGTTATG	TTTTGAGGAATACTTTAACCTGTG	A	C	119	60.36	CaChr7	217223	GRAS
CaTFSNP314	acgttggatgTGAGCTTTCCGTAGGTTGGT	acgttggatgATCAACGGTGGATGATGGAT	CGTTTGCAAGGTTGTGTAGTTAGT	G	A	211	59.73	CaChr7	888424	WRKY
CaTFSNP315	acgttggatgTTTAGAGGCGAGGTTGGTGT	acgttggatgACCACGACACATTCCATCAA	ATTCCGATGAAACCCCTCTT	G	A	209	59.73	CaChr7	1270983	bHLH
CaTFSNP316	acgttggatgAGCGTATTTCGGGGAAAAAG	acgttggatgCCTTTGGCTTCATCCCTACA	ACCCAACTACAATTCGGTCTTC	G	A	207	60.43	CaChr7	1799554	GNAT
CaTFSNP317	acgttggatgACACCTTTTCCGGTGAGTTG	acgttggatgGGTCAAATCCAAATCCCAAA	GGTCAAATCCAAATCCCAAAC	G	T	203	60.01	CaChr7	2712407	WRKY
CaTFSNP318	acgttggatgTAAACCGTTGATGGCGACTT	acgttggatgAGAAACGGCACGGTTTGATA	TTGGTGGTGGGTTTTTATTAG	G	A	109	60.5	CaChr7	3328637	Tify
CaTFSNP319	acgttggatgACCATGGAAACAGTGTAGGC	acgttggatgCGCGTGAGATTAAATTTTTCTTAGAT	CAACTTTAACTATCACAAGCTTTACA	T	C	96	57.51	CaChr7	3353684	ARF
CaTFSNP320	acgttggatgATCTTGGCTTCCGCAGAATC	acgttggatgAGGAGGCTGAATCACTCAAG	AAGTTCACACAAAAGAATCTTAT	C	T	96	47	CaChr7	3794570	SNF2
CaTFSNP321	acgttggatgCTCTCTTTCTCACTCATGCTCTT	acgttggatgTCAGAAATGAAAGATCGAGCA	CAGTCACTTCATTAATCATTCTCTT	T	C	120	57.12	CaChr7	4598134	C2C2-GATA
CaTFSNP322	acgttggatgCAGCATCTCTGCTTTGTCCA	acgttggatgCTGCCGGTTTTCCAAGTTAC	CAGCATGCAAATCCTTTACACA	T	C	201	60.14	CaChr7	4624953	ARR-B

SNP IDs*	1st Pre-PCR primer sequence	2nd Pre-PCR primer sequence	Unextended Primer sequence	SNP allele (ICC 4958)	SNP allele (ICC 17160)	Size (bp) of alleles amplified	Actual annealing temperature (0C)	Chromosomes	Physical positions (bp)	Transcription factors (TFs)
CaTFSNP323	acgttggatgCCACCTTCGATCCACAATCT	acgttggatgGGGTCGCAAGAGAGTATCCA	TCCCAAAAAATTACTCTCCACAA	T	C	211	59.93	CaChr7	5280205	mTERF
CaTFSNP324	acgttggatgAACCAAGTAAAGGTTTGG	acgttggatgAGAGAAGAAGGAGAAGGAT	AAAGAGAGAGAGAATATATAT	T	C	82	50.01	CaChr7	6812937	C2C2-YABBY
CaTFSNP325	acgttggatgTATCGTGGAATTCTGAAGCC	acgttggatgCTATTGAAATCGGATCTGAG	CCTCCATCCGCAAGATACTGATC	T	C	114	46.4	CaChr7	7863835	bHLH
CaTFSNP326	acgttggatgTGGTGGTGAAAACCAACAAC	acgttggatgACAACCACAAGCAGCACAAG	GCTGTTGATGGATGTTTGGA	G	A	191	59.29	CaChr7	8553174	zf-HD
CaTFSNP327	acgttggatgAAAGTTGATTGTGCAGTTTGACA	acgttggatgTCGAAGAGAATTCATGACCAAA	TTGCTAAAATTTTGTCATGTAGTTCAA	G	C	190	59.71	CaChr7	10016133	MYB-related
CaTFSNP328	acgttggatgGTTGCCGTGAAGAGGAAGAG	acgttggatgAAACCCAGTCCAAGTTGACG	TTTGATGTGAACTGTTTTAAAGA	G	T	119	59.99	CaChr7	10114849	AP2-EREBP
CaTFSNP329	acgttggatgTTGAGAAGCTCACAAAAGACTTG	acgttggatgTCGGCGTTTCACTTTCATAA	CAACAACAGTCTTGTTTTTCT	G	A	105	58.82	CaChr7	10251662	C2C2-GATA
CaTFSNP330	acgttggatgCAAGAAGTGATGGTGCAAGG	acgttggatgGCATGACTGCAAATCCTCCT	GACAAAATTAACAAAGCCCGAA	G	A	215	59.29	CaChr7	10485720	SRS
CaTFSNP331	acgttggatgGCCAAGGACTTGCCAACTAT	acgttggatgTGGTTTGCTGAGGAACTCAA	TTGAACACCATGATGAAGTG	T	C	99	59.2	CaChr7	10686471	ARR-B
CaTFSNP332	acgttggatgCTGAAGCAAACAGAGGTGGA	acgttggatgCACCACCACCACTAACACCA	ACCACCACTAACACCACCAAG	G	A	199	59.01	CaChr7	11192772	HB
CaTFSNP333	acgttggatgTGATTCTTTGGCTGAGAACG	acgttggatgCCGGTTGCAATTCATTCTTT	GGTTCGTATGCGGTTCG	A	T	92	59	CaChr7	11324293	bZIP
CaTFSNP334	acgttggatgTGAGAACTTTCCGATCACCG	acgttggatgTCGATGACGTCGGAAGAATC	GCCGACGGTGTGTTTGGAG	A	G	114	50.7	CaChr7	11546194	LOB
CaTFSNP335	acgttggatgGAGGTGGGTTATCCAGATTC	acgttggatgCTTCAAGCTTCCATCAGTTC	GTTCCATTTTTTGTTTGATCACT	A	G	86	48.9	CaChr7	14470793	FHA
CaTFSNP336	acgttggatgAATTCTTCCCAACTTGCCGC	acgttggatgAAACCAACAAGGCAGTGGGC	GGCTGCTTGGACTCG	G	A	102	49.8	CaChr7	17264124	MYB-related
CaTFSNP337	acgttggatgAATTGGTGGGTGGAAACCTT	acgttggatgTGCCCCTCAATTCTCAAAAG	GAATAACCCTTACCTCGATCCC	T	C	211	60.46	CaChr7	17317735	SET
CaTFSNP338	acgttggatgTTTGGCTGTCAAAAGCAAGAT	acgttggatgGCTGTGCAGGGTGTTCATTA	GAATGAGGGATCATATAGCATAAACC	G	A	203	59.87	CaChr7	17780711	bZIP
CaTFSNP339	acgttggatgGGTCAACCAGAAAAGATTCCA	acgttggatgCAGCAGCAAGTGCTCTCATC	CATCAATGATTCAAGAAATGAA	G	A	103	59.02	CaChr7	18389220	Jumonji
CaTFSNP340	acgttggatgCCTCACTTGTACAATCCAAC	acgttggatgGAGGGTGATAGAGGAGAATC	GTGGTGATGATGGTGT	C	T	102	46	CaChr7	20367403	bHLH
CaTFSNP341	acgttggatgCCAACAAAGAAAGCCTGAGAA	acgttggatgCAACAGGACTGCTTCAGTTTG	AAACATGTGGAGAGAGATTAACC	G	A	120	59.48	CaChr7	22826866	bHLH
CaTFSNP342	acgttggatgGGAAAAGAGGACCATAATGC	acgttggatgACAACACACACCACACACAG	AATTAAGCAGTCACGCA	C	A	109	46.9	CaChr7	27161185	Alfin-like
CaTFSNP343	acgttggatgATCCAACAAGTGCACCTATC	acgttggatgTGACTTGGTTTTCTCTAGGG	GGGATTTCTAAGGCAGTCTTTGG	C	T	116	47.6	CaChr7	33299885	Tify
CaTFSNP344	acgttggatgTTCCCAATTTCATTTACATTTCA	acgttggatgCGAACGATGATAGCGAGGAC	ACTAAACGTTACTCTCTTCTTCTTCTTCTT	G	C	99	58.4	CaChr7	39769428	C3H
CaTFSNP345	acgttggatgAGAGAGCACTCATGCCTATC	acgttggatgCCTCTTACTTTCCTGTTGCC	TGGCGGTCAGATCTTTTGA	T	A	104	52.1	CaChr7	42243517	RB
CaTFSNP346	acgttggatgTGTATGCTCGCTTGTTCTGC	acgttggatgACCATGCTCTCCAAAAGTCG	ACGGGAATTACAGCTTTCGAT	T	C	206	60.17	CaChr7	42245921	RB
CaTFSNP347	acgttggatgTGGTAGCGCAAATTCAAAGA	acgttggatgCCCGAGGGGACTACTAGGAA	GCGCAAATTCAAAGATGGG	T	C	211	59.44	CaChr7	42246393	RB
CaTFSNP348	acgttggatgCGGAGTGAGTCGGAATTTTC	acgttggatgTGACTCACTCGGAGTCAAAG	CTGAGGAATTACCAAAGGAGAACATTGT	G	T	96	53.8	CaChr7	43044483	CCAAT
CaTFSNP349	acgttggatgGTTCTTCAATTCAGCATAAG	acgttggatgCTGTGTTGGTGCCTATTTGG	TTGTTATGGAAGCAACTATTTGA	G	A	106	49.1	CaChr7	47237682	HB
CaTFSNP350	acgttggatgGCCTTGGCTTGTGTAATTCC	acgttggatgACCAAGTTGATGGAAAGCTG	GGGAAAGCTGGTACCC	T	C	101	45.8	CaChr8	156866	SNF2
CaTFSNP351	acgttggatgAATTTCCCTCAAGGTGGTGA	acgttggatgTAGTCCGCAAGAAGGACCAC	CAAGGTGGTGATAGTTTGGTTTT	G	A	234	59.38	CaChr8	498219	FHA
CaTFSNP352	acgttggatgCGTGGGATCAATTGAAGAGG	acgttggatgTGCAAGGCGGTTAATTTCTC	AGGACAGGAGAGAAATGTTGGA	G	A	200	60.45	CaChr8	510120	SNF2
CaTFSNP353	acgttggatgGCAAACGTGGCAATACCTCT	acgttggatgAGCAAAGAGGATGGCATCAA	TGGCAATACCTCTACCCCAATA	G	A	200	60.14	CaChr8	666619	GNAT
CaTFSNP354	acgttggatgTCAATGGTAAACCCGAAAGG	acgttggatgGTTCGTCACTTTGGCCATTT	GCAGATTCATACCTGATTGGAAA	G	A	208	59.79	CaChr8	891777	GNAT
CaTFSNP355	acgttggatgGTTGCCCTGACCATGTTCTT	acgttggatgTGGGTTCTTTTGGAAAGAGG	AAGAAACCCTTCTTCACCTTCTTC	T	C	203	59.97	CaChr8	1261904	C3H
CaTFSNP356	acgttggatgGGATAGGGAACAATTCACCG	acgttggatgTTGCCAAAGAACAACCTTCG	CGAACTCTGTTTCCTCTG	C	T	98	47.4	CaChr8	1434072	AP2-EREBP
CaTFSNP357	acgttggatgCCGGAATTGAGGCTACTAAGG	acgttggatgCGGCAATTAGCCTTCTTCAG	CTTGAATTTTCAAAGATAAACCTTCTTC	T	C	209	60.1	CaChr8	1753840	Jumonji
CaTFSNP358	acgttggatgGAATTGAAGATGTAAGGGAGGAAA	acgttggatgCATGTTGTTGCCACCAATTC	AGAAGAGCTGGTAAATTGCC	A	T	99	59.85	CaChr8	2011749	HB
CaTFSNP359	acgttggatgCGCTTTTGATTTTGTCACTCC	acgttggatgTGCGTATTGACGTGGCTTTA	CTTCTATCTCTTTATTTTTTTATTTCTGT	A	C	101	59.74	CaChr8	2580625	AP2-EREBP
CaTFSNP360	acgttggatgAGTGTTCCACCCATTTCGAC	acgttggatgGCAAAACCTGAAGACTGCAA	GATTTGCACTATCTAATCT	G	C	76	59.83	CaChr8	2664948	C2C2-GATA
CaTFSNP361	acgttggatgTGTGCGTGTCTATTTAGCATTG	acgttggatgTTGATGGAGGAATTAGTATGACAGA	TTGTCATAGAAAATGTCCTCAATAT	A	C	85	58.91	CaChr8	2704215	Tify
CaTFSNP362	acgttggatgGGTTTGGCATCCTACACAAG	acgttggatgCCCTTCATTCAGAAGAAGAG	GGTTTGATTTGATGTTGGTAAGGAGTA	C	T	98	51	CaChr8	2974401	ARR-B
CaTFSNP363	acgttggatgTCGTTATGGAGAAATTAAACACATC	acgttggatgTTTGGTTGGCATTGTTGAAA	CGTTATGGAGAAATTAAACACATC	G	T	112	58.56	CaChr8	3154021	bHLH
CaTFSNP364	acgttggatgAAACATCCTGTGCACCCTGT	acgttggatgACTGGGACCAACCATTTCAG	TATGAGGATGATGCCGATGA	T	C	195	60.43	CaChr8	3396641	BSD
CaTFSNP365	acgttggatgGAGAGAAAAGAAGGAATAAACAAATG	acgttggatgGCGGATGTGTTTGGAGAGAC	GAGAAAAGAAGGAATAAACAAATG	A	T	115	58.13	CaChr8	3456812	GNAT
CaTFSNP366	acgttggatgTGAAGCGTTAGCGGTTAATTG	acgttggatgCCGATCAACGACGATATCAA	CGGTTAATTGTGTTAACGCTTT	G	A	96	60.26	CaChr8	3521637	GRAS
CaTFSNP367	acgttggatgCCATTGAAGACAAGGGCGTA	acgttggatgCCTCCTCTTCCCAATTTTCC	AAACTCTAGCTTTAACTCACTTTCCATT	A	T	208	61.02	CaChr8	3682384	LOB
CaTFSNP368	acgttggatgGGAACCAAAAGGCTTCATCA	acgttggatgGGTGGCTCTGGTGATTTTGT	ATCCCACTCACAATCCGAA	G	T	204	60.05	CaChr8	3998501	AP2-EREBP
CaTFSNP369	acgttggatgTATGAGCCAGATAGAGCTCC	acgttggatgCTTCTTTGCACGACCACTTG	CCACTTGAAGGAGCA	C	T	116	45.2	CaChr8	4056195	SET
CaTFSNP370	acgttggatgGCCTCATCACGACTCAAACA	acgttggatgTGATGATGCACTTGAGCGTA	GAAAGCCGGCTTCATCTC	G	C	102	59.84	CaChr8	4176714	FHA
CaTFSNP371	acgttggatgTCATCATCAAATTCGCCTTG	acgttggatgCATATGCCAACGACTTCACG	GCGTCGTCACGCTGAGT	G	A	205	59.62	CaChr8	4421071	LOB
CaTFSNP372	acgttggatgCATTGATCTCTGTGGGGACA	acgttggatgACGACCAAGCAATTCAATCC	GTGGGGACATGTCTGAATC	G	A	101	59.47	CaChr8	4623320	HB
CaTFSNP373	acgttggatgGCATACTCTTCCTCAGACTC	acgttggatgCTATGGTGAGTTCTCAAGCG	CAAGAGGAGTTTAGATGTGTTCGTGT	A	T	93	50.6	CaChr8	5413114	SRS
CaTFSNP374	acgttggatgGCAGACTTGGAATGGCTCTC	acgttggatgGGTCTGAACCGGAGTTTTGA	GAGAATAAGCCCAAAAGCCC	G	T	198	59.96	CaChr8	5508790	C2C2-GATA
CaTFSNP375	acgttggatgCCCAAGTCTCCAAGTGGTGT	acgttggatgCAGTGGAGCCATTACAGCAA	TCATGCCCAATGTTGCTG	T	C	206	60	CaChr8	5564404	C3H
CaTFSNP376	acgttggatgAGTTACTCCTAAGCTTCTCC	acgttggatgGTCTGAAATCAATGATGACG	TGAAGATGATGATGAGTAAAGTTGGC	G	T	109	53.7	CaChr8	6618347	HMG
CaTFSNP377	acgttggatgAAGGAATTTTTCCAGCACCA	acgttggatgTGCCCTACTCTCTTCCCTCA	CTCCCTCTCAAGCTTTTCCAT	G	A	195	59.55	CaChr8	7520805	Trihelix
CaTFSNP378	acgttggatgTTGAGCTGCATGACGACAAT	acgttggatgTGCTAGAACATTCCCCTCCA	CCACAAAGTGATACTGTCACAC	A	T	108	60.42	CaChr8	7772710	E2F-DP
CaTFSNP379	acgttggatgGCCACCATCGTTGGATTTAG	acgttggatgAGGGACAGGAATCATTTGTG	AGGAACAAGGTGGAATTTGA	G	A	111	45.3	CaChr8	8362560	HB
CaTFSNP380	acgttggatgTCCAAGAGTGTTGCATTCAGA	acgttggatgGCTTCGTTGACTCGGTTTTT	TGATTTCTTGCACATATAGTATGG	T	C	99	59.43	CaChr8	11675561	Tify
CaTFSNP381	acgttggatgAAAACCCCTTCATCCGTACC	acgttggatgAACTGATGGAGATGTTTCTG	ATGTTTCTGATTTGTTTGGAA	G	A	108	46.9	CaChr8	12525828	MYB-related
CaTFSNP382	acgttggatgTTGGACCCAAGAAGTTTTGC	acgttggatgCAGTTGCACAAGAAGAGAAGGA	CTTCTTTAGCATCAAAACTTCCAAC	T	C	201	60.09	CaChr8	13906178	C2C2-CO-like
CaTFSNP383	acgttggatgAAAGACCGAACTTTCGCCTC	acgttggatgTGGTCGAATCGGCTTATAGG	GGGTTATATTAATTTCGGTGTTTC	A	G	111	46.1	CaChr8	14719923	SWI/SNF-SWI3
CaTFSNP384	acgttggatgATCTCAGCCATTGGATCTTC	acgttggatgGATACGAGCTAATCCAACGG	CCCAATCCAACGGTCCACG	T	A	86	49.5	CaChr8	16090020	C3H

*CaTFSNP *Cicer arietinum* Transcription Factor gene-derived SNP

two informative TF gene-derived SNPs in combination with seven microsatellite markers discriminated all the 94 cultivated and wild *Cicer* accessions from each other
