# Supplementary material for: Natural Allelic Diversity, Genetic Structure and Linkage Disequilibrium Pattern in Wild Chickpea
Source: PLoS One. 2014 Sep 15;9(9):e107484. doi: 10.1371/journal.pone.0107484 (PMC4164632; doi:10.1371/journal.pone.0107484)
Supplement: Table S4 — Polymorphic potential (represented by diverse statistical measures) detected by 478 genic and genomic microsatellite markers and 380 TF gene-derived SNP markers among 94 cultivated and wild Cicer accessions. (PDF) [file pone.0107484.s011.pdf]

Table S4: Polymorphic potential (represented by diverse statistical measures) detected by 478 genic and genomic microsatellite markers and 380 TF gene-derived SNP markers among 94 cultivated and wild *Cicer* accessions

Species/accessions used	Types of markers used	Number (%) of markers showing polymorphism	Number and range of alleles amplified	PIC (polymorphism information content)	Gene diversity	Heterozygosity	Major allele frequency
***C. arietinum* (12 accessions)**	Microsatellites	116 (23.5)	1134 & 1-4 (2.3088)	0.1767-0.6786 (0.2872)	0.1799-0.7528 (0.3058)	0.0000-0.0833 (0.000)	0.4167-0.9570 (0.9142)
	SNPs	42 (10.9)	730 & 1-2 (1.9099)	0.1767-0.3859 (0.2164)	0.1799-0.4457 (0.2187)	0.0000-0.0833 (0.0055)	0.7778-1 (0.9889)
	Total (Microsatellites + SNPs)	158 (18)	1864 & 1-4 (2.1250)	0.1911-0.6786 (0.2467)	0.2128-0.7528 (0.2559)	0.0000-0.000 (0.000)	0.4167-1 (0.9570)
***C. reticulatum* (16 accessions)**	Microsatellites	456 (92.5)	1873 & 1-8 (3.7941)	0.2103-0.8674 (0.5786)	0.2172-0.8969 (0.6372)	0.0000-0.0625 (0.0009)	0.25-1 (0.6634)
	SNPs	335 (87.2)	741 & 1-2 (1.9352)	0.1587-0.475 (0.3851)	0.1605-0.6 (0.4251)	0.0625-0.25 (0.0565)	0.5-1 (0.8386)
	Total (Microsatellites + SNPs)	791 (90.2)	2614 & 1-8 (3.2453)	0.1587-0.8674 (0.4679)	0.1605-0.8969 (0.5158)	0.0625-0.25 (0.0327)	0.25-1 (0.7637)
***C. echinospermum* (8 accessions)**	Microsatellites	348 (70.6)	1479 & 1-5 (3.0588)	0.2948-0.7675 (0.4530)	0.3188-0.8188 (0.4925)	0.0000-0.000 (0.000)	0.375-1 (0.7897)
	SNPs	84 (21.9)	614 & 1-2 (1.6198)	0.2103-0.4750 (0.2504)	0.2172-0.6 (0.2608)	0.1250-0.75 (0.0181)	0.5-1 (0.9576)
	Total (Microsatellites + SNPs)	432 (49.3)	2093 & 1-5 (2.5786)	0.2103-0.7675 (0.3371)	0.2172-0.8188 (0.3599)	0.1250-0.7500 (0.0103)	0.3750-1 (0.8858)
***C. judaicum* (22 accessions)**	Microsatellites	428 (86.8)	1804 & 1-7 (3.6618)	0.1830-0.8251 (0.5889)	0.1868-0.8619 (0.6573)	0.0476-0.07140 (0.0018)	0.3158-1 (0.6125)
	SNPs	198 (51.6)	691 & 1-2 (1.8055)	0.1454-0.4750 (0.2858)	0.1465-0.5 (0.2985)	0.0476-0.619 (0.0599)	0.5-1 (0.9314)
	Total (Microsatellites + SNPs)	626 (71.4)	2495 & 1-7 (3.000)	0.1454-0.8251 (0.4155)	0.1465-0.8619 (0.4520)	0.0476-0.619 (0.0349)	0.3158-1 (0.7950)

Species/accessions used	Types of markers used	Number (%) of markers showing polymorphism	Number of alleles amplified*	PIC (polymorphism information content)*	Gene diversity*	Heterozygosity	Major allele frequency*
***C. bijugum* (19 accessions)**	Microsatellites	276 (56)	1538 & 1-6 (3.1296)	0.1948-0.7797 (0.4367)	0.1997-0.8258 (0.4761)	0.0556 (0.001)	0.3684-1 (0.7939)
	SNPs	122 (31.8)	668 & 1-2 (1.7429)	0.1499-0.4750 (0.2799)	0.1512-0.5 (0.2867)	0.0526-0.6111 (0.0315)	0.5-1 (0.9270)
	Total (Microsatellites + SNPs)	398 (45.4)	2206 & 1-6 (2.5723)	0.1499-0.7797 (0.3503)	0.1512-0.8258 (0.3727)	0.0526-0.6111 (0.0181)	0.3684-1 (0.8650)
***C. pinnatifidum* (16 accessions)**	Microsatellites	362 (73.4)	1578 & 1-6 (3.2059)	0.2103-0.7816 (0.4874)	0.2172-0.8266 (0.5405)	0.0000-0.0000 (0.0000)	0.3750-1 (0.7299)
	SNPs	72 (18.7)	637 & 1-2 (1.6648)	0.1587-0.3733 (0.2628)	0.1605-0.5 (0.2725)	0.0625-0.5 (0.0447)	0.5417-1 (0.9433)
	Total (Microsatellites + SNPs)	434 (49.5)	2215 & 1-6 (2.6101)	0.1587-0.7816 (0.3588)	0.1605-0.8266 (0.3865)	0.0625-0.5 (0.0253)	0.3750-1 (0.8521)
***C. microphyllum* (1 accession)**	Microsatellites	NA	NA	NA	NA	NA	NA
	SNPs	NA	NA	NA	NA	NA	NA
	Total (Microsatellites + SNPs)	NA	NA	NA	NA	NA	NA
**Total (94 accessions**)	Microsatellites	478 (96.4)	2943 & 1-13 (5.9706)	0.1801-0.9777 (0.7534)	0.1822-0.9878 (0.8011)	0.0110-0.0132 (0.0007)	0.2065-1 (0.5140)
	SNPs	380 (98.9)	760 & 1-2 (1.9780)	0.1213-0.4735 (0.4108)	0.1215-0.5970 (0.4533)	0.0109-0.3152 (0.0386)	0.5385-1 (0.8313)
	Total (Microsatellites + SNPs)	858 (97.5)	3703 &1-13 (4.2579)	0.1213-0.9777 (0.5573)	0.315-0.9878 (0.6020)	0.0108-0.3152 (0.0225)	0.2065-1 (0.6956)

Values mentioned in the parentheses indicate the average estimates of their range obtained for each polymorphism statistics among 94 *Cicer* accessions
