# Supplementary material for: Natural Allelic Diversity, Genetic Structure and Linkage Disequilibrium Pattern in Wild Chickpea
Source: PLoS One. 2014 Sep 15;9(9):e107484. doi: 10.1371/journal.pone.0107484 (PMC4164632; doi:10.1371/journal.pone.0107484)
Supplement: Table S5 — Molecular diversity (represented by different diversity statistical measures) detected by 478 genic and genomic microsatellite markers and 380 TF gene-derived SNP markers among 94 cultivated and wild accessions within and between Cicer species. (PDF) [file pone.0107484.s012.pdf]

Table S5: Molecular diversity (represented by different diversity statistical measures) detected by 478 genic and genomic microsatellite markers and 380 TF gene-derived SNP markers among 94 cultivated and wild accessions within and between *Cicer* species

Within species	Genetic distance of microsatellite markers			Genetic distance of SNP markers			Genetic distance (microsatellite + SNP) markers		
	Minimum	Maximum	Mean	Minimum	Maximum	Mean	Minimum	Maximum	Mean
C. arietinum	0.1303 (ICCX810800-ICCV96970)	0.409 (ICC4958-IC296132), (ICCX810800-ICC4958)	0.3057	0.1033 (ICCV2-IC296131), (ICC95334-IC296131), (ICCV96329-IC296131)	0.2403 (ICCX810800-IC296131)	0.2171	0.1131 (ICCV96329-BG2024)	0.3057 (ICCX810800-ICC4958)	0.2585
C. reticulatum	0.2719 (ILWC229-ILWC219)	0.8418 (ILWC290-ILWC257)	0.6629	0.1178 (ILWC233-ILWC229)	0.6419 (ILWC237-ICC17160)	0.4102	0.2149 (ILWC229-ILWC219)	0.674 (ILWC237-ICC17160)	0.519
C. echinospermum	0.1597 (ILWC288-ICC17159)	0.7224 (ILWC35-IG135418)	0.4932	0.1034 (ILWC245-IG135418)	0.3333 (ILWC239-ICC17159)	0.2621	0.1859 (ILWC288-ICC17159)	0.455 (IG135418-ICC17159)	0.3798
C. judaicum	0.1455 (ILWC211-ILWC20)	0.9188 (ILWC45-ILWC30)	0.6784	0.1036 (ILWC278-ILWC273)	0.7556 (ILWC283-ILWC31)	0.2798	0.1289 (ILWC211-ILWC20)	0.8486 (ILWC38-ILWC283)	0.4634
C. bijugum	0.1444 (ILWC227-ILWC209)	0.74 (ILWC32-ILWC228)	0.4908	0.1444 (IG136792-ILWC209)	0.74 (IG136792-ILWC240)	0.4908	0.1336 (ILWC240-ILWC8)	0.5295 (ILWC32-ILWC42)	0.3523
C. pinnatifidum	0.1625 (ILWC248-ILWC226)	0.7323 (ILWC9-ILWC250), (ILWC29-ILWC289)	0.563	0.1037 (ILWC289-ILWC236)	0.92 (ILWC49-ILWC250)	0.2308	0.1504 (ILWC248-ILWC226)	0.5036 (ILWC49-ILWC250)	0.3927

Between species	Genetic distance (microsatellite + SNP) markers		
	Minimum	Maximum	Average
C. arietinum - *C. reticulatum*	0.1131 (ICCV96329-BG2024)	0.879 (ILWC36-ICCV2), (ILWC36-ICC4958)	0.6834
C. arietinum - *C. echinospermum*	0.1131 (ICCV96329-BG2024)	0.8796 (ICCV2-ILWC245), (ILWC45-PhuleG515)	0.6625
C. arietinum - *C. judaicum*	0.1131 (ICCV96329-BG2024)	0.9106 (ILWC283-ICCV96329)	0.6979
C. arietinum - *C. bijugum*	0.1131 (ICCV96329-BG2024)	0.8117 (ILWC42-ICCV2)	0.6418
C. arietinum - *C. pinnatifidum*	0.1131 (ICCV96329-BG2024)	0.8307 (ILWC250-ICCV2)	0.6651
C. arietinum - *C. microphyllum*	0.1131 (ICCV96329-BG2024)	0.7828 (*microphyllum*-ICCV96970)	0.4362
C. reticulatum - *C. echinospermum*	0.1859 (ILWC288-ICC17159)	0.7546 (ILWC245-ICC17160)	0.6468
C. reticulatum - *C. judaicum*	0.1286 (ILWC211-ILWC20)	0.9441 (ILWC283-ICC17160)	0.6767
C. reticulatum - *C. bijugum*	0.1336 (ILWC8-ILWC240)	0.7878 (ILWC285-ICC17160)	0.6387
C. reticulatum - *C. pinnatifidum*	0.1504 (ILWC248-ILWC226)	0.7965 (IG136820-ICC17160)	0.6724
C. reticulatum - *C. microphyllum*	0.2149 (ILWC229-ILWC219)	0.8602 (*microphyllum*-ILWC36)	0.6543
C. echinospermum - *C. judaicum*	0.1286 (ILWC211-ILWC20)	0.8486 (ILWC38-ILWC283)	0.61
C. echinospermum - *C. bijugum*	0.1336 (ILWC8-ILWC240)	0.6265 (ILWC32-IG135418)	0.5443
C. echinospermum - *C. pinnatifidum*	0.1504 (ILWC248-ILWC226)	0.6803 (ILWC246-ILWC250)	0.5863
C. echinospermum - *C. microphyllum*	0.1859 (ILWC288-ICC17159)	0.8658 (*microphyllum*-ILWC288)	0.5803
C. judaicum - *C. bijugum*	0.1286 (ILWC211-ILWC20)	0.8933 (ILWC32-ILWC283)	0.5862
C. judaicum - *C. pinnatifidum*	0.1286 (ILWC20-ILWC211)	0.8857 (ILWC22-ILWC283)	0.6151
C. judaicum - *C. microphyllum*	0.1286 (ILWC211-ILWC20)	0.9289 (*microphyllum*-ILWC283)	0.6076
C. bijugum - *C. pinnatifidum*	0.1336 (ILWC8-ILWC240)	0.6313 (ILWC49-ILWC228)	0.6473
C. bijugum - *C. microphyllum*	0.1336 (ILWC8-ILWC240)	0.8782 (ILWC277-*microphyllum*)	0.6018
C. pinnatifidum - *C. microphyllum*	0.1504 (ILWC248-ILWC226)	0.8192 (*microphyllum*-ILWC49)	0.6375
