# Supplementary material for: Natural Allelic Diversity, Genetic Structure and Linkage Disequilibrium Pattern in Wild Chickpea
Source: PLoS One. 2014 Sep 15;9(9):e107484. doi: 10.1371/journal.pone.0107484 (PMC4164632; doi:10.1371/journal.pone.0107484)
Supplement: Table S6 — Pair-wise estimates of genetic divergence (FST) and genetic distance among six model-based population groups. (PDF) [file pone.0107484.s013.pdf]

Table S6: Pair-wise estimates of genetic divergence (F_{ST}) and genetic distance among six model-based population groups

Populations	POP I	POP II	POP III	POP IV	POP V	POP VI
**POP I**	-	0.53	0.46	0.67	0.65	0.89
**POP II**	0.54	-	0.23	0.39	0.49	0.42
**POP III**	0.61	0.31	-	0.48	0.54	0.75
**POP IV**	0.68	0.52	0.47	-	0.79	0.69
**POP V**	0.78	0.61	0.58	0.65	-	0.83
**POP VI**	0.86	0.58	0.49	0.59	0.72	-

Pair-wise F_{ST} estimates are mentioned above the diagonal and genetic distance provided below the diagonal
