# Supplementary material for: Natural Allelic Diversity, Genetic Structure and Linkage Disequilibrium Pattern in Wild Chickpea
Source: PLoS One. 2014 Sep 15;9(9):e107484. doi: 10.1371/journal.pone.0107484 (PMC4164632; doi:10.1371/journal.pone.0107484)
Supplement: Figure S6 — Unrooted phylogenetic tree depicting the genetic relationships among 94 cultivated and wild accessions belonging to seven Cicer species based on Nei's genetic distance using 478 microsatellite markers (including 334 genic and 144 genomic microsatellite markers). Molecular classification clearly differentiated 94 accessions into six different clusters, which corresponds to their species and gene pools of origination with deviations. (PDF) [file pone.0107484.s006.pdf]

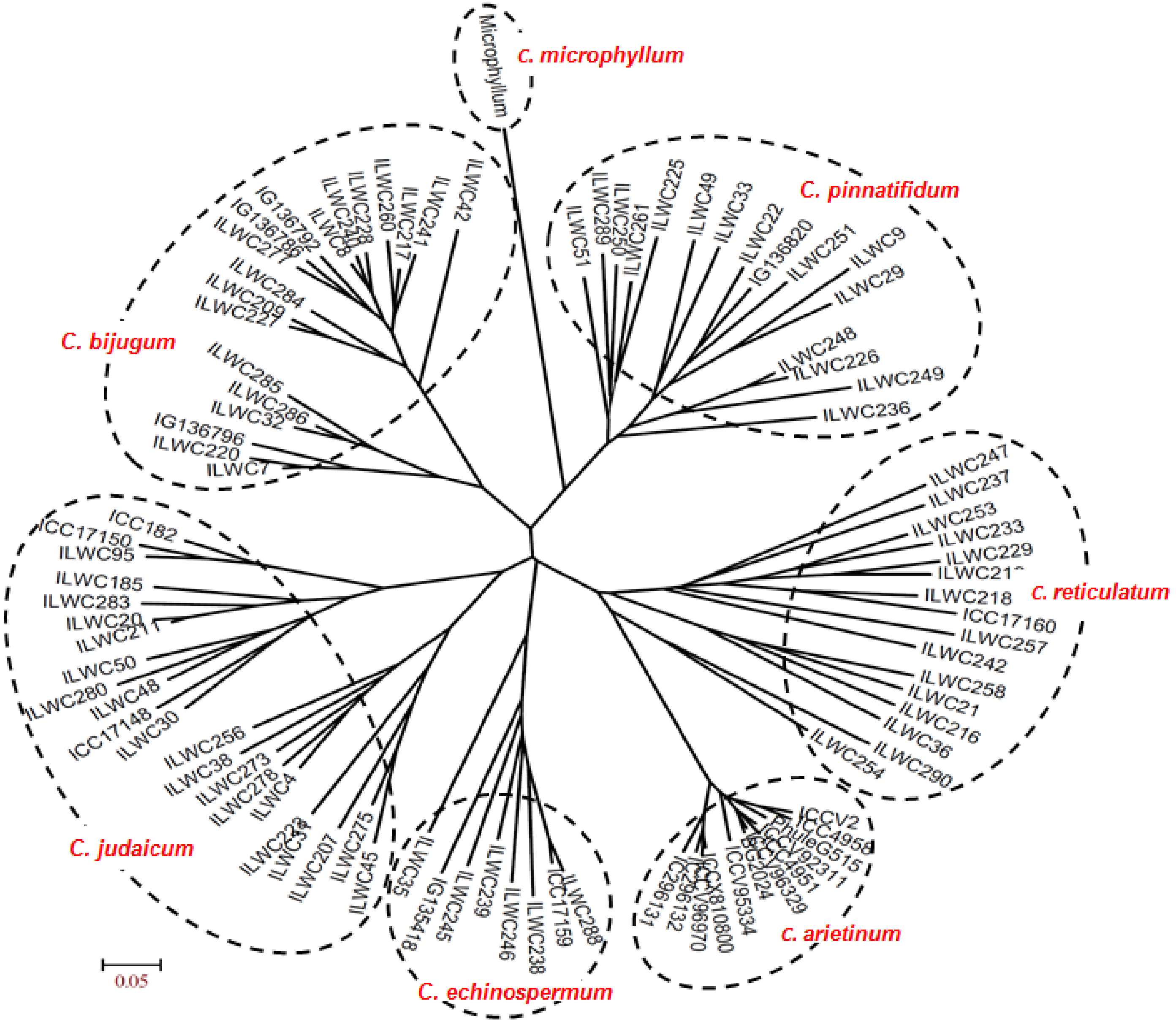

Figure S6: Unrooted phylogenetic tree depicting the genetic relationships among 94 cultivated and wild accessions belonging to seven *Cicer* species based on Nei’s genetic distance using 478 microsatellite markers (including 334 genic and 144 genomic microsatellite markers). Molecular classification clearly differentiated 94 accessions into six different clusters, which corresponds to their species and gene pools of origination with deviations.
