# Supplementary material for: Natural Allelic Diversity, Genetic Structure and Linkage Disequilibrium Pattern in Wild Chickpea
Source: PLoS One. 2014 Sep 15;9(9):e107484. doi: 10.1371/journal.pone.0107484 (PMC4164632; doi:10.1371/journal.pone.0107484)
Supplement: Table S7 — LD estimates in six model-based individual population groups and entire population as well as across eight chromosomes. (PDF) [file pone.0107484.s014.pdf]

Table S7: LD estimates in six model-based individual population groups and entire population as well as across eight chromosomes

Population groups	Global				Linked			Unlinked		
	Number of marker-pairs used	[a]Number of significant marker-pairs in LD	[b]Significant LD (%)	Extent of LD (r^2)	Number of significant marker-pairs in LD	Significant LD (%)	Extent of LD (r^2)	Number of significant marker-pairs in LD	Significant LD (%)	Extent of LD (r^2)
**POP I**	384117	949	2.47	0.25	179383	46.7	0.30	8182	2.13	0.22
**POP II**	384123	30768	80.1	0.20	337260	87.8	0.28	264661	68.9	0.18
**POP III**	384117	6876	1.79	0.27	158640	41.3	0.33	6261	1.63	0.25
**POP IV**	384108	14673	3.82	0.25	213948	55.7	0.27	13636	3.55	0.23
**POP V**	384122	6148	1.61	0.23	149423	38.9	0.28	5839	1.52	0.21
**POP VI**	384098	5531	1.44	0.19	128673	33.5	0.25	5070	1.32	0.17
**Total population groups**	384126	64572	16.81	0.22	163638	42.6	0.30	54277	14.13	0.20

Chromosomes	Linked			
	Number of marker-pairs used	Number of significant marker-pairs in LD	Significant LD (%)	Extent of LD (r^2)
**CaChr01**	5229	4983	95.3	0.26
**CaChr02**	4679	4464	95.4	0.28
**CaChr03**	7064	5658	80.1	0.31
**CaChr04**	5229	5198	99.4	0.28
**CaChr05**	7064	6054	85.7	0.30
**CaChr06**	6422	4373	68.1	0.27
**CaChr07**	5810	5595	96.3	0.40
**CaChr08**	4679	3977	85.0	0.29
**Total chromosomes**	5089	4361	85.6	0.31

[a]Percentage of microsatellite and SNP marker locus-pairs in significant ($P < 0.001$) LD

[b]Significant threshold ($P < 0.001$) at which pair-wise LD estimates is significant statistically
